# Thiophosphate photochemistry enables prebiotic access to sugars and terpenoid precursors

**DOI:** 10.1038/s41557-023-01251-9

**Published:** 2023-07-13

**Authors:** Dougal J. Ritson, John D. Sutherland

**Affiliations:** MRC – Laboratory of Molecular Biology, Cambridge Biomedical Campus, Cambridge, UK

**Keywords:** Origin of life, Organic chemistry

## Abstract

Over the past few years, evidence has accrued that demonstrates that terrestrial photochemical reactions could have provided numerous (proto)biomolecules with implications for the origin of life. This chemistry simply relies on UV light, inorganic sulfur species and hydrogen cyanide. Recently, we reported that, under the same conditions, reduced phosphorus species, such as those delivered by meteorites, can be oxidized to orthophosphate, generating thiophosphate in the process. Here we describe an investigation of the properties of thiophosphate as well as additional possible means for its formation on primitive Earth. We show that several reported prebiotic reactions, including the photoreduction of thioamides, carbonyl groups and cyanohydrins, can be markedly improved, and that tetroses and pentoses can be accessed from hydrogen cyanide through a Kiliani–Fischer-type process without progressing to higher sugars. We also demonstrate that thiophosphate allows photochemical reductive aminations, and that thiophosphate chemistry allows a plausible prebiotic synthesis of the C_5_ moieties used in extant terpene and terpenoid biosynthesis, namely dimethylallyl alcohol and isopentenyl alcohol.

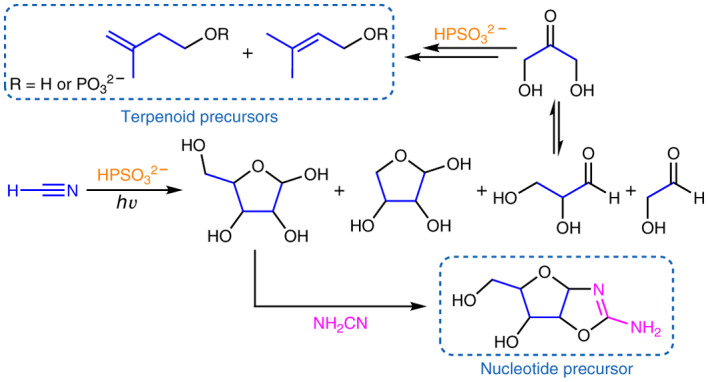

## Abstract (web summary)

The streamlined synthesis of multiple (proto)biomolecules from common starting materials is a key goal of prebiotic chemistry. Now, a one-pot synthesis of ribo-aminooxazoline (a precursor for prebiotic nucleotide synthesis) from HCN has been achieved. Additionally, the two moieties used in extant terpenoid biosynthesis have been accessed, with all carbon atoms also originating from HCN.

## Main

When contemplating the chemistry that gave rise to life, one of the fundamental questions to be addressed is that concerning the set of molecules that comprised the basis from which life could emerge. As this question cannot be answered by inference from biology alone, chemical experiments are required to identify reaction pathways that could have led from simple, environmentally available feedstock molecules to (proto)biomolecules. For productive coupling of the various precursors, it is reasonable to assume that the prebiotic synthesis of the basis set of molecules occurred in reasonably close proximity on primitive Earth, rather than in disparate and distanced environments, and consequently a common type of chemistry would be expected to give rise to numerous (proto)biomolecules. Where the chemistry was confined to, at least initially, must have been defined by geology and geochemistry, so all the chemical steps must comport with a geochemical scenario and the boundaries it imposes. Once this preliminary identification has been made, refinement of the prebiotic pathway or geochemical scenario can be informed and refined by its counterpart. For example, cyanamide (NH_2_CN) is an important prebiotic reagent, and the thermal conversion of Ca_2_[Fe(CN)_6_] to CaNCN with ensuing hydrolysis has been suggested as a source of NH_2_CN (ref. ^1^). However, under CO_2_-rich atmospheres, CaCO_3_ would be expected to precipitate rather than Ca_2_[Fe(CN)_6_] (ref. ^2^). Thus, if Ca_2_[Fe(CN)_6_] is required, a reduced atmosphere must have been present, which is the expected outcome from the impact of a large, reduced meteorite^3^. Cycling between geochemistry and prebiotic chemistry in this way should aid the improvement and plausibility of reaction pathways and the discovery of new reactions and reagents, in effect, acting as a type of triangulation^4^.

Recent reports from this laboratory have described the prebiotic synthesis of purine and pyrimidine nucleosides, precursors to amino acids and acyl glycerol phosphates, the components of the Krebs cycle, and a means of harnessing and supplying chemical energy to potentially drive this inanimate collection of molecules towards life^1,5–8^. The photochemical reduction of nitrile groups and thioamides (which can be derived from nitriles) to aldehydes constitutes a key reaction in these syntheses, and is repeated multiple times (Supplementary Fig. 1)^1^. The resulting aldehydes can be employed for further prebiotic reactions, such as the Strecker synthesis of amino acids. Importantly, there is a systems chemistry aspect to this network, which would have allowed flexibility and access to alternative products from common starting materials, depending on the conditions at a particular time or in a particular location on primitive Earth (Supplementary Discussion 1).

The geochemical scenario we have proposed that supports this chemistry has been described in detail several times^1,9–11^, but broadly speaking is envisaged to be land-based, occurring in an impact or post-impact environment, with prebiotic chemistry taking place in small streams (or possibly pools) that occasionally mix. The reagents and reactions required for the whole prebiotic network (Supplementary Fig. 1) are derived from and conform to this scenario, and primarily involve ultraviolet (UV) light, cyanide, ferrous iron and inorganic sulfur species such as HS^−^. For the prebiotic scheme to work most efficiently, some separation of the chemistries is desirable, so an interconnected system of small streams or flowing water is invoked that could allow mixing of reactants at various stages, for example, at a confluence^9–11^. During the course of our studies, we found that phosphite (HPO_3_^2−^) and hypophosphite (H_2_PO_2_^−^)—anoxic corrosion products of reduced Ni/Fe-P mineral species found in reduced meteorites^12,13^—could be oxidized to orthophosphate (PO_4_^3−^) by UV light and HS^−^ (ref. ^14^), thus providing one solution to the long-standing ‘phosphate problem’ and also complying with our geochemical and prebiotic model. We observed that thiophosphate (PSO_3_^3−^) was formed as an inevitable intermediate during this oxidation chemistry, which was noteworthy as we had previously reported PSO_3_^3−^ to be an efficient reagent for the formation of thioamides from nitriles and for phosphorylation reactions^15^. As the means of production of PSO_3_^3−^ matched our geochemical model, a full evaluation of its potential in the context of our prebiotic reaction network (Supplementary Fig. 1)^1^ was warranted^4^. Additionally, further assessment of the prebiotic availability and stability of PSO_3_^3−^ was made, as well as its in situ production and use (Supplementary Discussion 2).

## Results and discussion

### Photochemical reduction of nitriles

**Fig. 1:**
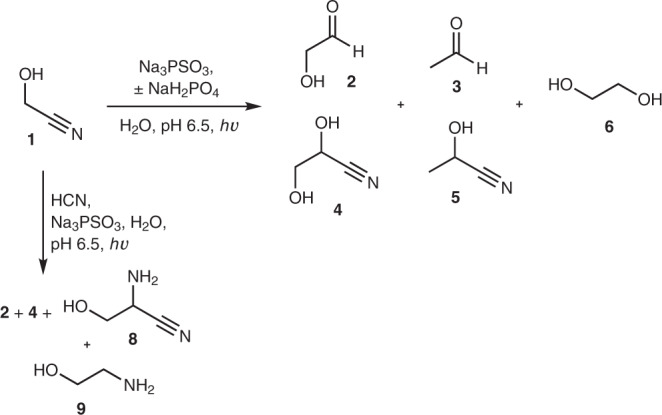
Photochemical reduction of glycolonitrile 1 or 1 and HCN by PSO_3_^3−^ yields multiple products of prebiotic interest. The major products of the reduction of **1** (horizontal reaction arrow) in the absence of phosphate are glycolaldehyde **2** and glyceronitrile **4**, with lesser amounts of acetaldehyde **3**, lactonitrile **5** and ethylene glycol **6**. The reaction is more efficient in the presence of phosphate, with an increased yield of **6**. In the presence of HCN (vertical reaction arrow), serine nitrile **8** and ethanolamine **9** are observed.

Initially, we wondered whether PSO_3_^3−^ possessed similar photochemical properties to HS^−^, which could mean HS^−^ and PSO_3_^3−^ were interchangeable in our prebiotic syntheses (Supplementary Fig. 1). This seemed an attractive possibility, as PSO_3_^3−^ is not volatile and can be formed at geologically relevant concentrations of H_2_S/HS^−^ (ref. ^14^). As a representative example, we irradiated glycolonitrile **1** (20 mM) with low-pressure Hg lamps (principal emission at 254–256 nm) in the presence of PSO_3_^3−^ (20 mM) at pH 6.5. Reduction of **1** was efficient, the major products being glycolaldehyde **2** (∼19%) and glyceronitrile **4** (∼9%), with lesser amounts of acetaldehyde **3**, EtOH and lactonitrile **5** also being formed (Fig. 1, horizontal reaction arrow; Supplementary Figs. 2–4 and Supplementary Table 1). Under identical conditions using NaSH as the reductant, **2** and **4** were produced in ∼6% and ∼5% yields, respectively, and only a trace amount of **3** was present (Supplementary Fig. 2 and Supplementary Table 1). Increasing the amount of PSO_3_^3−^ (1.5 equiv.) gave slightly improved yields after 1 h of irradiation (Supplementary Fig. 2 and Supplementary Table 1). Irradiating the reaction for longer did not really affect the yield of **2**, as α-deoxygenation of **2** became competitive with the reduction of **1**, giving increased yields of **3** and subsequent overreduction of **3** to EtOH (Supplementary Figs. 2 and 5 and Supplementary Table 1).

During the reaction the pH increased (pH ∼ 9.2 after 1 h of irradiation), so we repeated the reduction of **1** with the inclusion of phosphate (PO_4_^3−^, 20 mM, 1 equiv.) as a pH buffer (pH 6.5), which is consistent with the geochemical scenario. Interestingly, reduction turned out to be much more efficient in the presence of phosphate, with glycolaldehyde **2** and glyceronitrile **4** being formed in ∼34% and ∼4% yields, respectively, after 1 h, in addition to increased amounts of ethylene glycol **6** (Supplementary Fig. 6 and Supplementary Table 1). Clearly, PSO_3_^3−^ is a far more effective reductant than HS^−^ alone and is at least as efficient as the CuCN/HS^−^ system we have previously reported^1,16^.

**Fig. 2:**
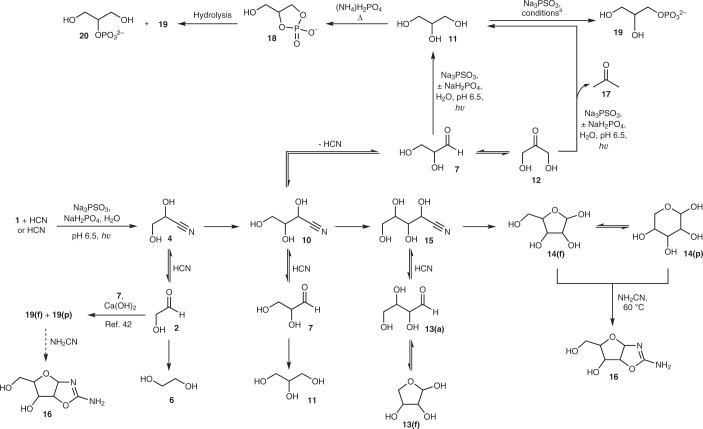
Photochemical reductive homologation of HCN or glycolonitrile 1 + HCN by PSO_3_^3−^ yields various higher sugars, depending on slight differences in the starting conditions. Unless stated, reactions were carried out at ambient temperature. For convenience, aldehydes are depicted as the carbonyl compounds, but would also exist as hydrates in water. ^a^(±)CH_2_CHCN, formamide, 70 °C; formamide, CH_2_CHCN; Fe(CN)_6_^3−^, formamide; *hν*, formamide (Supplementary Table 7).

We then attempted the in situ generation and homologation of glycolaldehyde **2** to glyceraldehyde **7** starting from **1** (20 mM) and HCN (20 mM). After 1 h of irradiation, the crude ^1^H NMR spectrum indicated that **2**, its cyanohydrin **4** and lactonitrile **5** had been formed in ∼4%, ∼24% and ∼3% yields, respectively (Fig. 1, vertical reaction arrow; Supplementary Fig. 7 and Supplementary Tables 2.1 and 2.2). Serine nitrile **8** (∼16%) was also present, suggesting that the nitrogen contained in HCN could be directly fixed into amino acids without the need for the addition of NH_3_. Additionally, a small amount of glycolaldehyde imine had been reduced to ethanolamine **9**. Repeating the reaction in the presence of PO_4_^3−^ (60 mM) at pH 6.5, we detected **7** (∼5%) and its cyanohydrin **10** (∼13%), **2** (∼6%), **4** (∼9%), glycerol **11** (∼6%) and **6** (∼6%) after 1 h of irradiation (Fig. 2, Supplementary Fig. 8 and Supplementary Tables 2.1 and 2.2). Evaporation of hydrogen cyanide (HCN) from the crude reaction mixture gave **2** and **7** in ∼13% and ∼14% overall yields, respectively, and left **6** and **11** unchanged (Fig. 2 and Supplementary Fig. 9). This solution was then left for two weeks at room temperature, after which **7** had partially equilibrated to dihydroxyacetone **12** (giving a ratio of ∼1:1 of **7**:**12**, although the end point for equilibration will favour **12** (ref. ^17^); Fig. 2 and Supplementary Fig. 9), thus allowing the prebiotic synthesis of valine and leucine as previously described^1^. Although HCN could have been potentially concentrated through the intermediacy of ferrocyanide^1,9,10^, and can undergo reductive homologation to **7** under the same conditions (Supplementary Fig. 10 and Supplementary Tables 2.1 and 2.2), glycolonitrile **1** may be an equally attractive starting material given its surprisingly high boiling point (102–104 °C at 6 mmHg (refs. ^18,19^)), which may have allowed its concentration in groundwater to some degree (Supplementary Fig. 11).

As the Kiliani–Fischer-like homologation of **1** to glyceraldehyde **7** using thiophosphate had proven so efficient, we repeated the reaction to see if the reduction products of glyceraldehyde cyanohydrin **10** were formed. The tetroses **13** (threose **13-t** and erythrose **13-e**) were present in the crude reaction mixture (∼19%), in addition to **2** (∼8%) and **7** (∼8%; Fig. 2, Supplementary Figs. 12 and 13 and Supplementary Tables 3.1, 3.2 and 4). Little, if any, diastereoselectivity was observed, with **13**-**t**:**13**-**e** of ∼0.9:1 (Supplementary Fig. 13). The total yield of identifiable products was ∼72%, comprising ∼4% C_1_, ∼27% C_2_, ∼22% C_3_ and ∼19 % C_4_ compounds (Supplementary Table 4).

In an attempt to make higher sugars, we increased the amount of PSO_3_^3−^ (100 mM) and HCN (45 mM) in the starting mixture and lowered the amount of glycolonitrile **1** (7.5 mM). After 2.75 h of irradiation, the total yield of identifiable products was ∼60%, comprising ∼1% C_1_, ∼8% C_2_, ∼11% C_3_, ∼11% C_4_ and ∼29% C_5_ compounds (Supplementary Figs. 14 and 15 and Supplementary Tables 3.1, 3.2 and 5). Although the presence of a small amount of **1** improved the efficiency of the synthesis of pentoses **14**, its inclusion was not vital, with the Kiliani–Fischer-like process forming **14** in ∼19% yield starting from HCN alone (Supplementary Fig. 16 and Supplementary Tables 3.1 and 3.2). This constitutes an alternative one-pot, prebiotically plausible synthesis of the tetroses **13** and pentoses **14** from a C_1_ feedstock molecule to that reported by Butlerow in 1861 (ref. ^20^). If it is assumed that, at each step of HCN homologation, imine hydrolysis and cyanohydrin formation proceed quantitatively (which is clearly not the case given the presence of **6**, **9** and **11**), the photochemical reduction of nitriles proceeds at an average of ∼65% yield. Indeed, the similarity between a crude reaction mixture resulting from reduction of HCN, or **1** and HCN, and a roughly equimolar mixture of **2**, **7**, **13**-**t**, **14**, **6** and **11**, is notable (Supplementary Fig. 17). Interestingly, the hexoses were not observed in ^1^H NMR spectra (Supplementary Fig. 18), even though the concentration of **14** was comparable to that of the C_2_, C_3_ and C_4_ sugars, and must be ascribed to the fact that the pentoses **14** exist almost exclusively in furanosyl **14(f)** and pyranosyl **14(p)** forms (in aqueous solution at circumneutral pH), with only traces (∼0.01–0.04%) of the carbonyl forms being present (Fig. 2 and Supplementary Fig. 19)^21^. Accordingly, a regulatory mechanism exists, and, by the time the pentoses **14** are being formed, the concentration of HCN is too low to force the equilibria in favour of pentose cyanohydrins and so further homologation of **14** is inhibited.

We then wondered if the transient protection of the carbonyls of **14**, afforded by the cyclic hemiacetals **14(f)** and **14(p)**, could have assisted the accumulation of **14** on early Earth. The crude reaction mixture resulting from the reduction of **1** and HCN by PSO_3_^3−^ was thus evaporated to the point of dryness and the solid dissolved in D_2_O. ^1^H NMR spectroscopy revealed that the C_2_, C_3_ and C_4_ sugars had decomposed (potentially via polymerization), but the pentoses **14** were still present (<30% decomposition of **14** taking place; Supplementary Fig. 20).

Competition experiments were then performed to examine the stability of **14** to photoreduction and photochemistry, and, in both the presence and absence of PSO_3_^3−^, **14**-**x** displayed excellent resistance to UV-promoted decomposition relative to **13**-**t** and **2** (chosen as representative examples), with the partly ring-closed tetrose **13**-**t** (∼90% **13(f)**)^22^ being approximately twice as stable as **2** (Supplementary Figs. 21 and 22 and Supplementary Table 6). Consequently, the photochemical reduction of **1** and HCN was repeated and the irradiation extended to 5 h. After this time, **2** and **7** were effectively absent from the crude reaction mixture and **13** had undergone partial decomposition, yet the concentration of **14** had increased relative to the 2.5 h time point (Supplementary Fig. 23). Although this presents a second potential mechanism by which the pentoses **14** could have been selected, it is not clear whether this enrichment is required, as the selective crystallization of *ribo-*aminooxazoline **16**-**r** from a mixture of sugars has already been demonstrated (the aminooxazolines being formed by reaction of the sugars with cyanamide)^23^, and the elaboration of **16**-**r** into purine and pyrimidine nucleosides has recently been reported^5^.

To connect to these previous studies, **1** and HCN were subjected to photoreduction using PSO_3_^3−^, then excess HCN was evaporated from the solution and NH_2_CN added. After heating for 2 h at 60 °C, conversion of **14** to **16** was observed (Fig. 2 and Supplementary Fig. 24; CaCN_2_ was found to work equally efficiently as NH_2_CN (ref. ^1^), Supplementary Fig. 25). The procedure was then repeated using HCN alone as the carbon feedstock, and *ribo-*aminooxazoline **16**-**r** was formed in ∼1% overall yield (Supplementary Fig. 26). Thus, telescoped (without work-up/purification/isolation of intermediates, but with addition of a reagent at a later point), prebiotically plausible syntheses of **16**-**r** from feedstock molecules thought to have been available on early Earth have been achieved, in 12 steps from glycolonitrile **1** or 15 steps from HCN.

### Photochemical reduction of carbonyl groups

Glycerol **11**, a key component of all cell membranes, had been formed in several of the previous reactions, and probably resulted from the reduction of glyceraldehyde **7**. We therefore assumed that **11** could be formed from the reduction of dihydroxyacetone **12** or glyceraldehyde **7**, and consequently attempted the photoreduction of **12** (15 mM) by 2 equiv. of PSO_3_^3−^. This yielded **11** in ∼51% yield after 0.5 h—almost doubling the yield previously reported, and produced approximately ten times faster, while requiring less than half of the amount of reductant (Supplementary Fig. 27)^1^. Reduction of glyceraldehyde **7** under the same conditions gave ∼63% of **11** after 45-min irradiation (Supplementary Fig. 28). The reductions of both **12** and **7** to **11** by PSO_3_^3−^ were accompanied by α-deoxygenation side reactions leading to acetone **17**, and its 1,2-reduction product isopropanol, and smaller amounts of propan-1,2-diol, from **12**, and propan-1,3-diol from **7** (Supplementary Figs. 27 and 28). As we had found previously (Supplementary Fig. 6), inclusion of PO_4_^3−^ increased the amount of 1,2-reduction, forming **11** in ∼64% yield from **12** after a 0.5-h reaction, or ∼71% yield starting from **7** after a 45-min reaction (Supplementary Fig. 29). Evaporation of water also removed the bulk of the side products and left glycerol **11** in a highly pure and concentrated form (Supplementary Fig. 30). The one-pot synthesis of **11** could be favoured by increasing the amount of reductant (PSO_3_^3−^, 75 mM) and subjecting glycolonitrile **1** (10 mM) and HCN (10 mM) to irradiation for 2.5 h. Glycerol **11** was formed in ∼27% yield, alongside ethylene glycol **6** (∼32% yield) and minor amounts of the α-deoxygenated products mentioned above (Supplementary Fig. 31).

The ultimate fate of PSO_3_^3−^ is to be converted into PO_4_^3−^, so the two components required to make glycerol phosphates **18**, **19** and **20** under standard prebiotic phosphorylating conditions are formed in the same location. However, given that PSO_3_^3−^ itself is an effective phosphorylating agent^15^, we considered reactions of **11** with PSO_3_^3−^. Heating **11** with PSO_3_^3−^ and acrylonitrile at 70 °C in formamide for 3 h (ref. ^15^), or in the absence of acrylonitrile for 10 h, gave **19** and **20**, although we found that phosphorylation also occurred at room temperature in the presence of acrylonitrile (Fig. 2, Supplementary Figs. 32–35 and Supplementary Table 7). Low-temperature phosphorylations were also made possible by activation of PSO_3_^3−^ with ferricyanide or by photolysis in formamide (Supplementary Figs. 36 and 37 and Supplementary Table 7). Glycerol-1-phosphate **19** and glycerol-2-phosphate **20** were obtained in 14–30% yield and 4–9% yield, respectively, depending on the conditions used (Supplementary Figs. 32–37 and Supplementary Table 7). Furthermore, as low-temperature phosphorylations were possible, cyclic phosphates such as **18**, generated under more conventional conditions^1,24^, could be avoided if desired.

### Reductive aminations and in situ azole synthesis

The production of ethanolamine **9** (Fig. 1 and Supplementary Fig. 7), alongside glycolaldehyde **2**, interested us, as it has been found that **9** is an effective catalyst for the polymerization of nucleoside-2′,3′-cyclic phosphates^25^, and it has also been shown that **2** is a precursor for the prebiotic synthesis of *ribo-*nucleoside-2′,3′-cyclic phosphates via 2-aminooxazole **21** (refs. ^26,27^). Initially, we irradiated glycolaldehyde **2** (25 mM) in the presence of NH_3_/NH_4_ (150 mM) and PSO_3_^3−^ (100 mM) at pH 9.2 and observed ∼14% of ethanolamine **9** after 6 h of irradiation, in addition to EtOH (∼16%) and ethylene glycol **6** (∼11%; Supplementary Fig. 38 and Supplementary Table 8). Such high concentrations of ammonia on primitive Earth may have occurred after dissolution of Mg_3_N_2_ (refs. ^1,9^), for example, but would probably only have been short-lived given the volatility of NH_3_, and so we repeated the reaction at pH 7.0 in the presence of phosphate buffer when the majority of ammonia is protonated. Gratifyingly, **9** was still formed, albeit in reduced yield (∼6% after 6 h), in addition to EtOH (∼12%), but the most dramatic change was the yield of **6**, ∼82% after 6 h of reaction (Supplementary Fig. 38 and Supplementary Table 8). Unsurprisingly, increasing the concentration of NH_3_/NH_4_ increased the yield of **9**.

**Fig. 3:**
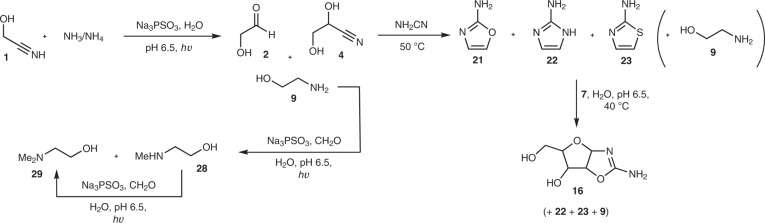
Prebiotic photochemical reductive aminations and telescoped synthesis of azoles 21, 22 and 23. Irradiation of glycolonitrile **1** and NH_4_Cl in the presence of PSO_3_^3−^ generates glycolaldehyde **2** (and its cyanohydrin **4**) and ethanolamine **9**. Addition of cyanamide results in the generation of azoles **21**, **22** and **23**. Irradiation of **9** and formaldehyde in the presence of PSO_3_^3−^ affords *N-*methyl ethanolamine **28** and *N*,*N*-dimethyl ethanolamine **29**.

We then attempted the telescoped synthesis of ethanolamine **9** and 2-aminooxazole **21** starting from glycolonitrile **1** (25 mM) and NH_4_Cl (75 mM) at neutral pH. After irradiating the starting mixture for 1.5 h, **2** (and its cyanohydrin **4**) and **9** were present in ∼32% and ∼1% yield, respectively (Fig. 3). Cyanamide (50 mM) was added to the crude reaction, and the solution was heated to 50 °C for 20 h, after which time 2-aminooxazole **21** could be seen in the ^1^H NMR spectrum in ∼8% yield (based on **1**, Fig. 3; Supplementary Fig. 39 and Supplementary Table 9). In addition to **21**, we determined that 2-aminoimidazole **22** and 2-aminothiazole **23** were also present and had been formed in ∼2% and ∼9% yield, respectively (ethanolamine **9** was unchanged after reaction with cyanamide, Fig. 3; Supplementary Fig. 39 and Supplementary Table 9). Omitting ammonia from the reaction yielded **21** and **23** in ∼7% and ∼8% yield (3 equiv. PSO_3_^3−^) or ∼16% and ∼2% yield (1 equiv. PSO_3_^3−^), respectively (Supplementary Fig. 40 and Supplementary Table 9). It would seem unlikely that mercaptoacetaldehyde would form under the reaction conditions, which may give some indication of the mechanism(s) that lead to azole formation from **2** and NH_2_CN (Supplementary Fig. 41).

Ethanolamine **9**, 2-aminooxazole **21**, 2-aminoimidazole **22** and 2-aminothiazole **23** could thus have been localized on early Earth. This may have implications for the prebiotic synthesis of (oligo)nucleotides, as dihydroxyacetone **12** (the thermodynamically preferred triose isomer) can be converted to glyceraldehyde **7** by **23** (ref. ^17^)—the triose isomer required for reaction with **21** to yield *ribo-*aminooxazoline **16**-**r** (ref. ^26^). This in turn, can be converted to *ribo-*nucleoside-2′,3′-cyclic phosphates^27^, and **9** is a catalyst for the polymerization of *ribo-*nucleoside-2′,3′-cyclic phosphates, producing short oligonucleotides^25^. Finally, nucleotides activated by **22** have been suggested as labile surrogates for nucleotide triphosphates, allowing both the non-enzymatic copying of oligonucleotides^28^ and ribozyme-catalysed RNA ligation^29^. Although the full ramifications of this result warrant more detailed investigation, it is beyond the scope of the current paper. We do note, however, that the reaction of **7** (50 mM) with 2-aminooxazole **21** (50 mM) to give the pentose aminooxazolines **16** in the presence of **9** (6 mM), **22** (19 mM) and **23** (60 mM, constituting the ratio in which **9**, **21**, **22** and **23** were formed; Supplementary Fig. 39) was still possible, giving **16** in ∼30% yield (cf. ∼40% yield in the absence of **9**, **21**, **22** and **23**; Fig. 3 and Supplementary Fig. 42). Thus, the synthesis of short oligonucleotides via *ribo-*nucleoside-2′,3′-cyclic phosphates is potentially still viable from this mixture.

Although nucleotide-5′-phosphoro-2-aminoimidazolides are good substrates for the abiotic copying of oligomeric nucleotides^28^, a source of chemical activation is still required. We recently showed that MeNH_2_ **24** can be converted to methyl isonitrile (MeNC) **25** under prebiotic conditions using ferrocyanide salts (nitroprusside), and that the intermediate isonitrile complex **26** is stable and can be concentrated by evaporation (Supplementary Fig. 43)^7^. UV light causes **26** to release **25**, which, in the presence of an aldehyde or under mildly acidic conditions, can activate 5′-nucleotide monophosphates (Supplementary Fig. 43)^7,8,30^. The activated nucleotides can be intercepted in a highly efficient manner by imidazoles, such as **22**, which form nucleotide-5′-phosphoro-2-aminoimidazolides in up to 76% yield (Supplementary Fig. 43)^7,8,30^. Consequently, we attempted the reductive amination of formaldehyde **27** (25 mM) with NH_4_Cl (150 mM) using PSO_3_^3−^ (75 mM) and UV light at either pH 9.2 or pH 7.0 in the presence of phosphate. We were pleased to observe MeNH_2_ **24** was formed in ∼11% yield after 2-h reaction at pH 9.2 and in ∼26% yield at pH 7.0 (Supplementary Fig. 44 and Supplementary Table 10). Halving the concentration of NH_4_Cl returned ∼10% of **24** at either pH 7.0 or 9.2 (Supplementary Table 10).

Given the success of the reductive methylation of ammonia using **27** and PSO_3_^3−^, we next considered the methylation of ethanolamine **9**. Irradiation of **9** (30 mM) and **27** (90 mM) in the presence of PSO_3_^3−^ (120 mM) gave *N-*methyl ethanolamine **28** in ∼46% yield and *N*,*N*-dimethyl ethanolamine **29** in ∼3% yield after 4 h of reaction (Supplementary Fig. 45). Addition of more reductant (40 mM) followed by two cycles of addition of **27** (20 mM) then irradiation resulted in further methylation of the amines, giving **28** in ∼56% yield and **29** in ∼25% yield with only ∼19% of **9** remaining (Supplementary Fig. 45), a process that could presumably be repeated further. Phosphorylation of **9**, **28** and **29** (50 mM each) was achieved using PSO_3_^3−^ (50 mM) and Fe(CN_6_)^3−^ (100 mM) in formamide, leading to *O*-phosphorylethanolamine **30**, *N*-methyl ethanolamine phosphate **31** and *N*,*N*-dimethyl ethanolamine phosphate **32** in ∼16%, ∼12% and ∼26% yield, respectively (Supplementary Figs. 46 and 47). These products are reminiscent of the intermediates used by phosphatidylethanolamine *N*-methyltransferase in bacteria^31^, and so a facile transition to the biosynthesis of primitive versions of phosphatidylcholine can be imagined.

### Prebiotic synthesis of terpene precursors

**Fig. 4:**
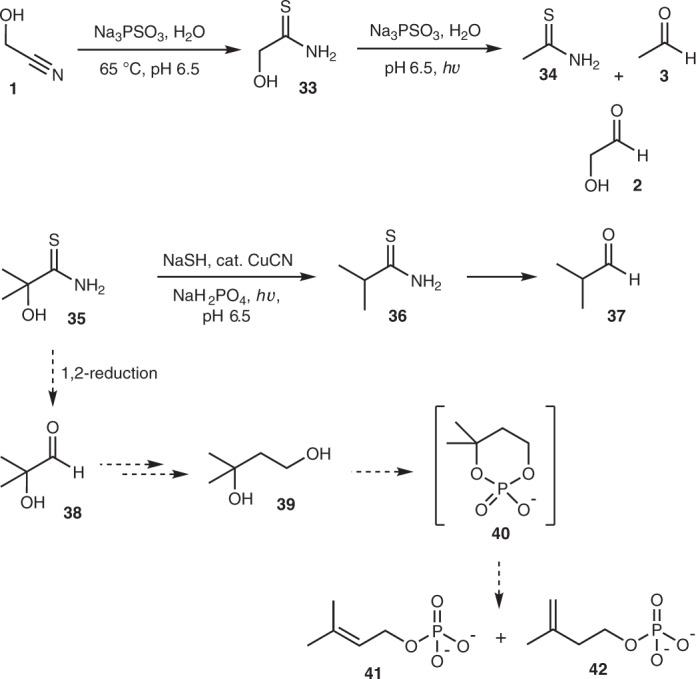
The unexpected synthesis of glycolaldehyde 2 from photochemical reduction of α-hydroxythioacetamide 33 by thiophosphate, and a possible route to potential isoprenoid precursors 41 and 42. The synthesis of glycolaldehyde **2** is shown at the top, and the possible route to potential isoprenoid precursors **41** and **42** is shown by dotted arrows. Unless stated, reactions were carried out at ambient temperature. For convenience, unidirectional reaction arrows are used and aldehydes are depicted as carbonyl compounds, although they would also exist as hydrates in water.

We then considered thioamides, functional groups that are integral to our protometabolic network (Supplementary Fig. 1)^1^. We had already shown that cyanohydrins can be cleanly converted to α-hydroxythioamides by incubation with PSO_3_^3−^ (ref. ^15^), and it now appeared that the products of thiolysis could be reduced by the same reagent. As a representative example, glycolonitrile **1** (20 mM) was reacted with PSO_3_^3−^ (80 mM) at 65 °C and pH 6.5 for 20 h, which gave α-hydroxythioacetamide **33** in ∼97% yield (Fig. 4 and Supplementary Fig. 48). Addition of a second portion of PSO_3_^3−^ (80 mM) followed by irradiation at 254 nm for 4 h gave the expected reduction products thioacetamide **34** (∼23%) and acetaldehyde **3** (∼17%), and unexpectedly glycolaldehyde **2** (∼14%; Fig. 4 and Supplementary Fig. 48). Previously, when an α-hydroxythioamide, such as 2,2-dimethyl-2-hydroxythioacetamide **35**, was subjected to HS^−^/CuCN photoreduction, clean α-deoxygenation occurred to furnish the corresponding thioamide (that is, isobutyryl thioamide **36**), and ensuing reduction then afforded the corresponding aldehyde (that is, isobutyraldehyde **37**), and α-hydroxyaldehydes were not observed (Fig. 4)^1^. This unexpected mode of reactivity of thiophosphate presented an intriguing possibility. If the reduction of **35** yielded some 2-hydroxy-2-methylpropanal **38**, homologation (via cyanide addition, thiolysis and reduction) could lead to 3-methyl-1,3-butanediol **39** (Fig. 4). We expected phosphorylation of **39** under heating conditions to temporarily form the cyclic phosphate **40**, which should be primed for elimination of the phosphate dianion at the tertiary centre, thereby affording dimethylallyl phosphate **41** and isopentenyl phosphate **42**. These structures are analogous to the biosynthetic precursors of terpenes (dimethylallyl pyrophosphate and isopentenyl pyrophosphate), which are generally used to make secondary metabolites. Archaea, however, are absolutely dependent on linear isoprenoids for cell-membrane formation, constituting the hydrophobic moiety of their phospholipids.

**Fig. 5:**
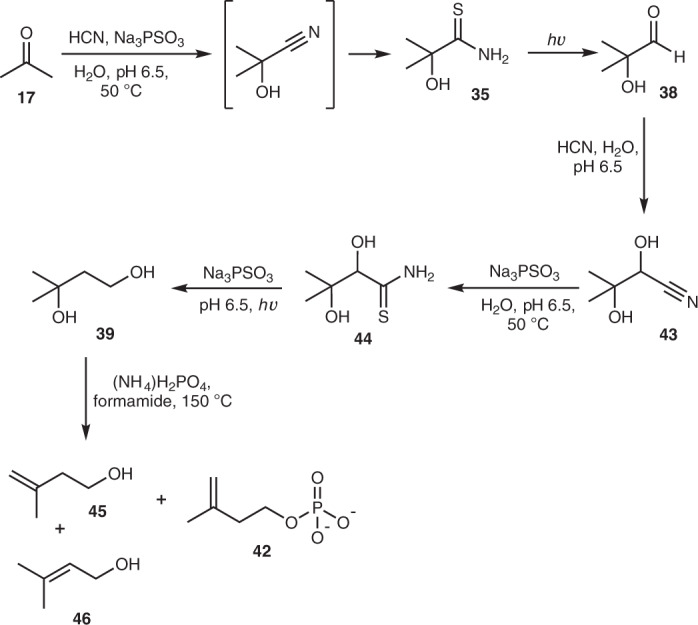
Prebiotic route to potential isoprenoid precursors. Reactions were carried out at ambient temperature unless stated otherwise. For convenience, unidirectional reaction arrows are used and aldehydes are depicted as carbonyl compounds, although they would also exist as hydrates in water.

Acetone **17** (50 mM), a by-product from glycerol **11** synthesis (Fig. 2, Supplementary Fig. 27 and ref. ^1^), was incubated with HCN (100 mM) and PSO_3_^3−^ (250 mM) at 50 °C for 4 h, which afforded ∼29% of **35** (Fig. 5 and Supplementary Fig. 49). Gentle sparging with N_2_ left **35** as the sole product (Supplementary Fig. 49). The remaining solution was then irradiated for 4 h, after which time all of **35** had been consumed (Supplementary Fig. 49). Addition of HCN (30 mM) resulted in the formation of cyanohydrin **43** in ∼27% yield starting from **35**, or ∼8% overall yield from starting from acetone **17** (four steps, Fig. 5 and Supplementary Fig. 49). Although some α-deoxygenation took place, none of the resulting thioamide **36** or its secondary reduction product (isobutyraldehyde **37**) were observed in the ^1^H NMR spectrum; only the fully reduced product, isobutanol, was present (∼20% yield; Supplementary Fig. 49).

We investigated the next sequence of reactions starting from a prepared sample of **43** (90 mM). Thiolysis of **43** by PSO_3_^3−^ (545 mM) proceeded more slowly than some cyanohydrins, but α-hydroxythioamide **44** was obtained in good yield (∼64%) after two days (Supplementary Fig. 50 and Supplementary Table 11). Although lower concentrations of PSO_3_^3−^ could be used for the reaction, thiolysis was slower. Using PSO_3_^3−^ (200 mM) under analogous conditions, ∼41% of **44** had formed after three days of reaction (Supplementary Table 11). Addition of further PSO_3_^3−^, followed by UV irradiation, fortuitously gave the α-deoxygenated, fully reduced compound **39** as the major product (∼40% yield starting from **38**, Fig. 5 and Supplementary Fig. 50; 3-methyl-1,2,3-butanetriol was present, but only in ∼16% yield, Supplementary Fig. 50). Although the steps in this synthesis are high-yielding, sequential addition of cyanide would probably be required to form **39** most efficiently, and this could potentially have been achieved by the confluence of a cyanide-rich stream with the reaction stream^9–11^.

Recognizing that forcing conditions would be needed to eliminate H_2_O from 3-methyl-1,3-butanediol **39**, we dissolved **39** (100 mM) in formamide with ammonium phosphate (300 mM), and heated the reaction at 150 °C. After 22 h, isopentenyl alcohol **45** (∼48% yield), isopentenyl phosphate **42** (∼6% yield) and dimethylallyl alcohol **46** (∼3% yield) could be observed by ^1^H NMR spectroscopy (Fig. 5 and Supplementary Figs. 51 and 52). For laboratory convenience we heated the reactions to 150 °C, but the reaction gave comparable results when run for longer periods of time at lower temperatures (Supplementary Fig. 53 and Supplementary Table 12), and on the geologic timescale, even lower temperatures may have sufficed (although not explored, metals ions and acid catalysis may also lead to elimination of water from **39**). It was not possible to determine whether the elimination step occurs via cyclic phosphate **40**, although we did note the presence of **40** in the reaction mixture. As thiophosphate can phosphorylate alcohols under much milder conditions, we mixed **46** (50 mM) with PSO_3_^3−^ (100 mM) in formamide and activated PSO_3_^3−^ (either with ferricyanide or UV light), which gave dimethylallyl phosphate **41** in ∼10% yield (Supplementary Figs. 54 and 55).

## Conclusions

Evaluation of the first prebiotic synthesis of activated pyrimidine ribonucleotides^26^, in terms of a geological/geochemical scenario that could satisfy the requirements of that synthetic route, led us to consider meteoritic impacts^1^. While potentially providing a localized, abundant source of phosphorus species^12,13,32^, HCN would also be generated by those same impacts^33,34^. This suggests that cyanometallates would be present in the same location. We then showed that these compounds are effective catalysts for the photochemical reduction of HCN and nitriles using inorganic sulfur species as the stoichiometric reductant^1,9,16,35,36^. Returning full circle, we reassessed the processing of meteoritie-derived phosphorus species under photochemical conditions in the presence of HS^−^/H_2_S and found that hypophosphite and phosphite could be oxidized to phosphate, during which a new reagent was formed, PSO_3_^3−^ (ref. ^14^). In a further iteration of this process, we examined PSO_3_^3−^ in the context of our cyanosulfidic network, which led to marked improvements over existing prebiotic pathways and allowed entirely different ones. Most notably, we have demonstrated a one-pot, prebiotic synthesis of C_2_–C_5_ sugars from a C_1_ feedstock and prebiotic access to isopentenyl alcohol **45** and dimethylallyl alcohol **46**, or phosphates thereof—the biological precursors of terpenes.

Although the mixture of C_2_–C_5_ sugars can be enriched in pentoses **14**, several reported prebiotic routes to ribonucleotides requiring (d)-ribose^37–39^ will be confounded by the lack of stereoselectivity in the current synthesis of **14**. *Ribo-*aminooxazoline **16**-**r** offers a potential solution to this problem given its diastereoselective, and even enantioselective, crystallization, which can occur under appropriate conditions^23,40,41^. Although **16**-**r** can be easily accessed from the C_2_–C_5_ sugar mixture (Fig. 2 and Supplementary Figs. 24–26), it could also be derived after aldol reaction of glycolaldehyde **2** and glyceraldehyde **7** (Fig. 2)^42^, which can be formed in almost equimolar amounts from HCN or HCN and **1** using thiophosphate as the reductant (Supplementary Figs. 8–10). A third avenue for the synthesis of **16**-**r** also exists; this proceeds through 2-aminooxazole **21** by reaction with **7** (Fig. 3 and Supplementary Fig. 42)^26^, possibly requiring 2-aminothiazole **23** (Supplementary Figs. 39 and 40) to separate **2** and **7** (ref. ^17^). Studies can now be undertaken to determine which of these routes is the most promising for a telescoped synthesis of (deoxy)ribonucleosides from feedstock molecules.

## Methods

### General experimental

All reactions were run at least twice, and the NMR spectra shown in the Supplementary Information are representative examples. Reagents and solvents were bought from Sigma-Aldrich, Alfa Aesar and Santa Cruz Biotechnology and were used without further purification, apart from thiophosphate, which had variable amounts of impurities and H_2_O present (Supplementary Information, page 1). Reagents were weighed using a Sartorius AX124 M-Pact analytical balance and small volumes were measured using a Gilson Pipetman system. Photochemical reactions were carried out using a Rayonet RPR-200 photochemical reactor chamber, with cooling fans switched on (the internal temperature of the unit when operational was ~40 °C) and fitted with low-pressure RPR-2537A mercury lamps purchased from Rayonet (principal emission at 254 nm). Hellman QS Spectrosil 10.0-mm quartz cuvettes with four UV-transparent windows were used for photochemical reactions. A Mettler Toledo SevenMulti pH/mV module fitted with a Thermo Scientific Orion 8103BN pH probe was used to measure pH, and deoxygenation of solvents and HCl/NaOH solutions was achieved by sparging with argon for 20–30 min before use. Although deoxygenation of HCl and NaOH solutions, used to adjust the pH of the reactions, may have altered the concentrations of these solutions, it was not deemed to be important, as adjustment of the pH of the reaction was our only consideration. Although using solvents that had not been deoxygenated was not anticipated to have a substantial effect on the outcome of the reactions, we wished to ensure that the reaction of O_2_ with any sulfur species was kept to a minimum, while also comporting with the expected anoxic environment of early Earth. The removal of dissolved O_2_ from aqueous media by sparging with an inert gas has been shown to be effective, although trace amounts of dissolved O_2_ may remain^43^. Rigorous exclusion of O_2_ from the solutions after deoxygenation was not possible, particularly when checking/adjusting the pH where the solution would typically be exposed to the atmosphere for ∼45 s. For comparison, a thiolysis and reduction reaction was run in ‘oxygenated’ solvents that were not deoxygenated before use, using thiophosphate (Supplementary Figs. 75 and 76). ^1^H, ^31^P and ^13^C NMR spectra were acquired using a Bruker Ultrashield 400 Plus instrument (at 400.1, 162.0 and 100.6 MHz, respectively); alternatively, ^1^H and ^31^P NMR spectra were recorded using a Bruker Ascend 400 instrument (at 400.2 and 162.0 MHz, respectively) using solvent suppression to collect ^1^H NMR data if reactions were run in a D_2_O/H_2_O mixture. If the spectra were unsatisfactory, a small amount of D_2_O was added to the NMR sample and the spectrum was reacquired. Yields were determined by the relative integration of signals in the ^1^H or ^31^P NMR spectra or by the addition of a standard of known volume and concentration, and relative integration to this signal. For quantitative integration of phosphorus NMR signals, we used a Bruker Avance-II 500 spectrometer with broadband cryogenic probe at a ^31^P frequency of 202.4 MHz. Quantitative integration of phosphorus NMR signals was achieved by determining the relaxation time (*T*_1_) for the nucleus, which was slowest to relax (thiophosphate, 8.4 s). ^31^P quantitative NMR (qNMR) spectra were acquired with a 30° pulse flip angle, >7 × *T*_1_ relaxation delay (giving >99% relaxation of ^31^P nuclei), 160-ppm spectral width, 128,000 acquisition data points and the spectrum offset close to the midpoint frequency of peaks being integrated. Spectra were processed and quantified using TopSpin version 3.2 software. Coupling constants (*J*) are given in hertz and the notations d, t and q represent the multiplicities doublet, triplet and quartet. Chemical shifts (*δ*) are given in ppm. Mass spectra were recorded with an Agilent Technologies 6130 Quadrupole LCMS using positive and negative electron spray ionization.

## Online content

Any methods, additional references, Nature Portfolio reporting summaries, source data, extended data, supplementary information, acknowledgements, peer review information; details of author contributions and competing interests; and statements of data and code availability are available at 10.1038/s41557-023-01251-9.

### Supplementary information

Supplementary Information Supplementary Figs. 1–76, Tables 1–12, Discussions 1 and 2 and experimental procedures.

## Data Availability

All data associated with this study are available in the published Article and its Supplementary Information.
