# Supplementary material for: Thiophosphate photochemistry enables prebiotic access to sugars and terpenoid precursors
Source: Nat Chem. Author manuscript; Available in PMC 2023 Oct 5. (PMC10533393; doi:10.1038/s41557-023-01251-9)
Supplement: TOC alt text description [file EMS176196-supplement-TOC_alt_text_description.docx]

Hydrogen cyanide is on the left of a reaction arrow proceeding to the right. Under UV irradiation in the presence of thiophosphate, this is shown to be converted to pentoses, tetroses, glyceraldehyde and glycolaldehyde. Glyceraldehyde is shown to be in equilibrium with dihydroxyacetone which can then be converted, after several steps using thiophosphate, to dimethyl allylalcohol and isopentenyl alcohol, or their monophosphates, which are terpenoid precursors in extant biology. The pentoses are depicted to undergo reaction with cyanamide, giving the pentose aminooxazolines, one of which is a precursor to nucleotides.
