# Supplementary material for: Thiophosphate photochemistry enables prebiotic access to sugars and terpenoid precursors
Source: Nat Chem. Author manuscript; Available in PMC 2023 Oct 5. (PMC10533393; doi:10.1038/s41557-023-01251-9)
Supplement: TOC summary [file EMS176196-supplement-TOC_summary.docx]

The streamlined synthesis of multiple (proto)biomolecules from common starting materials is a key goal of prebiotic chemistry. A one-pot synthesis of *ribo-*aminooxazoline (a precursor for prebiotic nucleotide synthesis) from HCN has now been achieved. Additionally, the two moieties used in extant terpenoid biosynthesis have been accessed, with all carbon atoms also originating from HCN.
