# Supplementary material for: Thiophosphate photochemistry enables prebiotic access to sugars and terpenoid precursors
Source: Nat Chem. 2023 Jul 13;15(10):1470–7. doi: 10.1038/s41557-023-01251-9 (PMC10533393; doi:10.1038/s41557-023-01251-9)
Supplement: Supplementary file 1 [file 41557_2023_1251_MOESM1_ESM.pdf]

nature chemistry

Article

https://doi.org/10.1038/s41557-023-01251-9

Thiophosphate photochemistry enables prebiotic access to sugars and terpenoid precursors

In the format provided by the authors and unedited

Table of contents

General Procedures	1
Supplementary Figs. 1 – 62, Supplementary Tables and experimental details	2 – 80
Synthetic procedures to make standards	81 – 82
NMR Spectra of standards (Supplementary Figs. 63 – 74)	83 – 90
Control experiments in ‘oxygenated’ solvents (Supplementary Figs. 75 – 76)	91 – 92
Supplementary Discussion 1 and 2	93 – 96
References	97 – 98

Supplementary Tables

Supplementary Table 1	5
Supplementary Table 2.1 & 2.2	11
Supplementary Table 3.1 & 3.2	19
Supplementary Table 4	20
Supplementary Table 5	23
Supplementary Table 6	31
Supplementary Table 7	43
Supplementary Table 8	50
Supplementary Table 9	52
Supplementary Table 10	58
Supplementary Table 11	67
Supplementary Table 12	70

Determining H_2O content and impurities of commercial $Na_3PSO_3.xH_2O$

Typically, > 90% is given as the purity of the reagent and the molecular formula is stated as $Na_3PO_3S.xH_2O$. We found that there were *ca.* 10% impurities (identified as PO_4^{3-} and pyrophosphate ($P_2O_7^{4-}$)) but the H_2O content was highly variable depending upon batch and manufacturer, and this had to be factored into stoichiometric calculations – something not taken into consideration previously.[15]

A ^{31}P NMR spectrum of the material was acquired to determine the purity of the reagent.

An Eppendorf tube was weighed and a sample of $Na_3PSO_3.xH_2O$ weighed accurately into it. The sample was dissolved in thoroughly degassed H_2O, frozen and lyophilised then re-weighed. The procedure was repeated with 2 other samples and the average taken to obtain the percentage of removable H_2O contained within the batch.

General procedure 1

As the second and third pK_a of H_3PSO_3 are 5.4 and 10.1, respectively,[44] we assumed the dibasic form would be most prebiotically plausible. Hence, for formamide reactions, we dissolved the required amount (taking into account the H_2O content, *vide supra*) of commercial tribasic reagent in degassed H_2O and adjusted the pH to 7.0 – 7.3 with degassed 1 M HCl. The sample was then frozen in liquid N_2 and lyophilised.

Supplementary Figs. 1 – 62 and experimental details

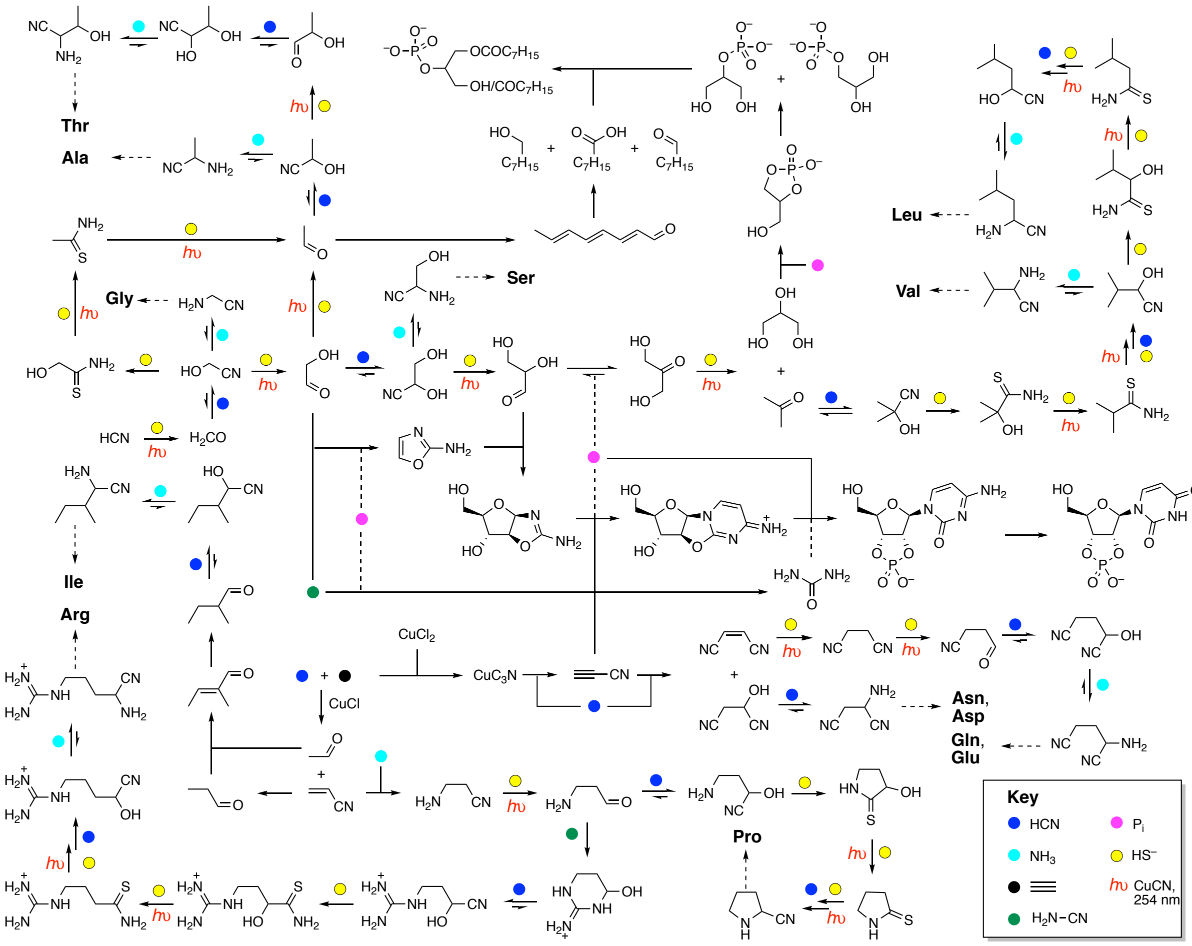

Supplementary Fig. 1 The photoreductive cyanosulfidic protometabolic network previously described.[1] Here, it is depicted in a different format, and the key shows the reagents required to access all intermediates and products of the network. It is clear from the scheme that the same reagents are used repeatedly, and that a fundamental step is the photoreduction of nitrile bonds or their thioamide derivatives (thioamides are integral to our protometabolic network as their formation allows for perturbation of cyanohydrin equilibria, and α-functionalised thioamides can be reduced to thioamides and thence to aldehydes (under one electron reducing conditions)). Any reagent which can affect any step in the network, therefore, is of potential significance.

Procedure 1

In an Eppendorf tube, $Na_3PSO_3.xH_2O$ (the purity and water content were predetermined and accounted for, 1 equiv. or 1.5 equiv.) was dissolved with degassed 10% D_2O in H_2O (1 mL) and the pH was adjusted to 6.5 with degassed HCl. The volume was made up to 2 mL with degassed 10% D_2O in H_2O, glycolonitrile **1** (0.040 mmol, 4.0 μL) was added and the solution was transferred to a quartz cuvette and sealed. The reaction was irradiated for the desired amount of time after which it was analysed by ^1H NMR spectroscopy.

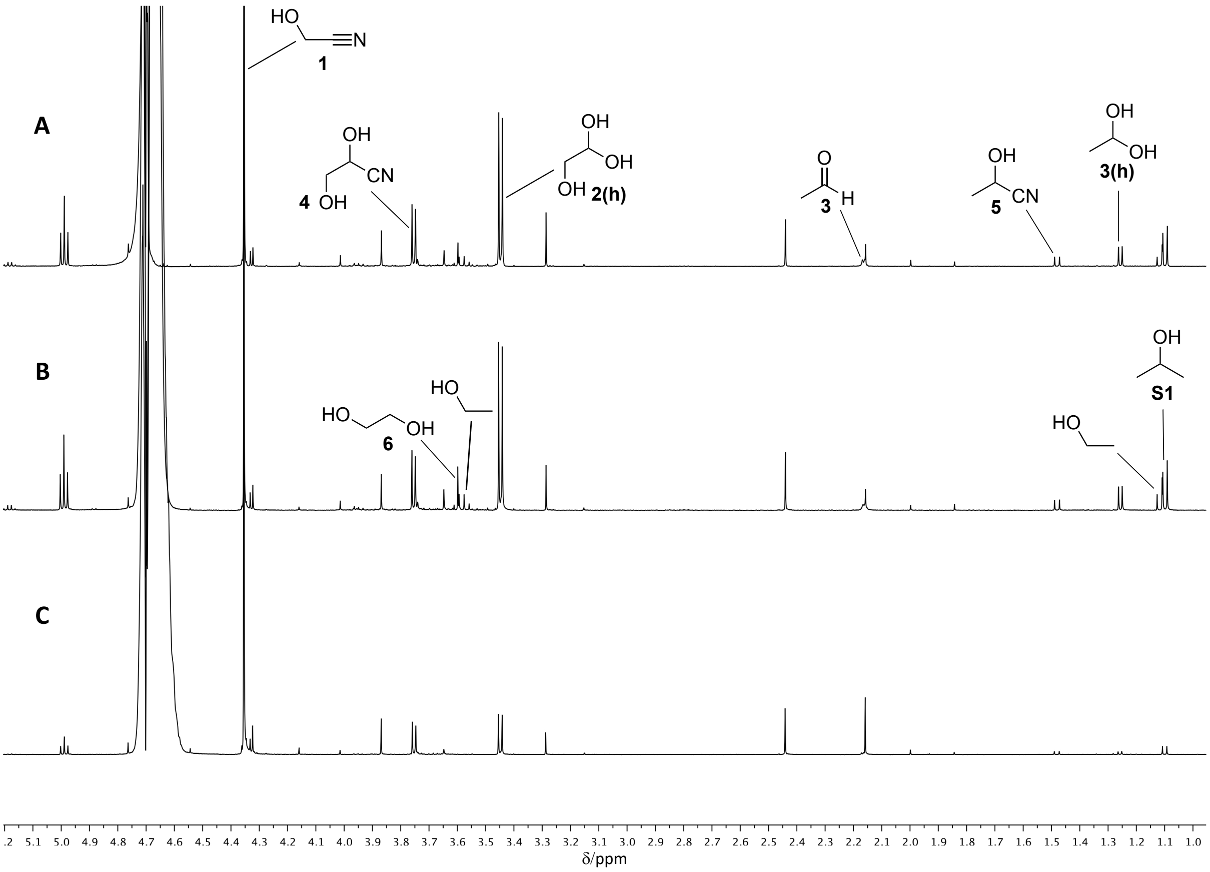

Supplementary Fig. 2 Photochemic al reduction of glycolo nitrile **1** using thiophosphate. A – ^1H NMR Spectrum of the reaction according to Procedure 1 (1 equiv. Na_3PSO_3) after 1 h irradiation; B – ^1H NMR Spectrum of the reaction according to Procedure 1 (1.5 equiv. Na_3PSO_3) after 1 h irradiation; C – As spectrum A, but NaSH used in place of Na_3PSO_3. The notation **X(h)** signifies the hydrate of an aldehyde, **X**. There is a similar amont of acetaldehyde **3** and lactonitrile **5** in spectrums A and B, but spectrum C is almost devoid of these compounds. In spectrums A and B a small amount of EtOH (annotated in spectrum B) has started to form (~ 2% yield in both spectra), but there is a signal almost superimposed on the EtOH triplet at 1.10 ppm which corresponds to isopropanol **S1** (d, J = 6.2, 6H). We later identified that this came from the reduction of acetone, an impurity in several commercial sources of **1**, see Supplementary Fig. 3. A small signal for the residual acetone can be seen in spectrums A and

B, just to the right of acetaldehyde **3** (at 2.15 ppm), and clearly in spectrum C where reduction to **S1** has not yet taken place. The signals at 3.30 ppm and 2.44 ppm are due to MeOH and thioacetamide, respectively (although thioacetamide can be further reduced to acetaldehyde **3** the reduction of thioamides takes place much more slowly than cyanohydrins (see Supplementary Fig. 48), hence we believe the production of **3** comes mainly from deoxygenation of **2**, see also Supplementary Fig. 4). Yields estimated by addition of disodium fumarate (50 mM solution, 50 μL into 450 μL of crude reaction) as a standard and relative integration of ^{1}H NMR signals.

Supplementary Table 1 Yields of products resulting from photoreduction of glycolonitrile **1**

Entry	Spectrum	Glycolonitrile **1**	Reductant	PO_4^{3-}	Irradiation time	Yield					
						Glycolaldehyde **2**	Glyceronitrile **4**	Acetaldehyde[a] **3**	Lactonitrile **5**	Ethylene glycol **6**	EtOH
1	Supp. Fig. 2, Spectrum A	20 mM	PSO_3^{3-} (20 mM)	–	1 h	~19%	~9%	~3%	trace	trace	~2%
2	Supp. Fig. 2, Spectrum C	20 mM	HS^- (20 mM)	–	1 h	~6%	~5%	trace	trace	–	–
3	Supp. Fig. 2, spectrum B	20 mM	PSO_3^{3-} (30 mM)	–	1 h	~21%	~8%	~4%	trace	trace	~2%
4	Supp. Fig. 5, Spectrum A	20 mM	PSO_3^{3-} (20 mM)	–	2 h	~20%	~8%	~5%	trace	trace	~3%
5	Supp. Fig. 5, Spectrum B	20 mM	PSO_3^{3-} (30 mM)	–	2 h	~22%	~7%	~6%	trace	~2%	~6%
6	Supp. Fig. 6, Spectrum B	20 mM	PSO_3^{3-} (20 mM)	20 mM	1 h	~34%	~4%	~4%	trace	~6%	~4%

trace - a yield of 1% or less

[a] - yield of **3** constitutes carbonyl + hydrate

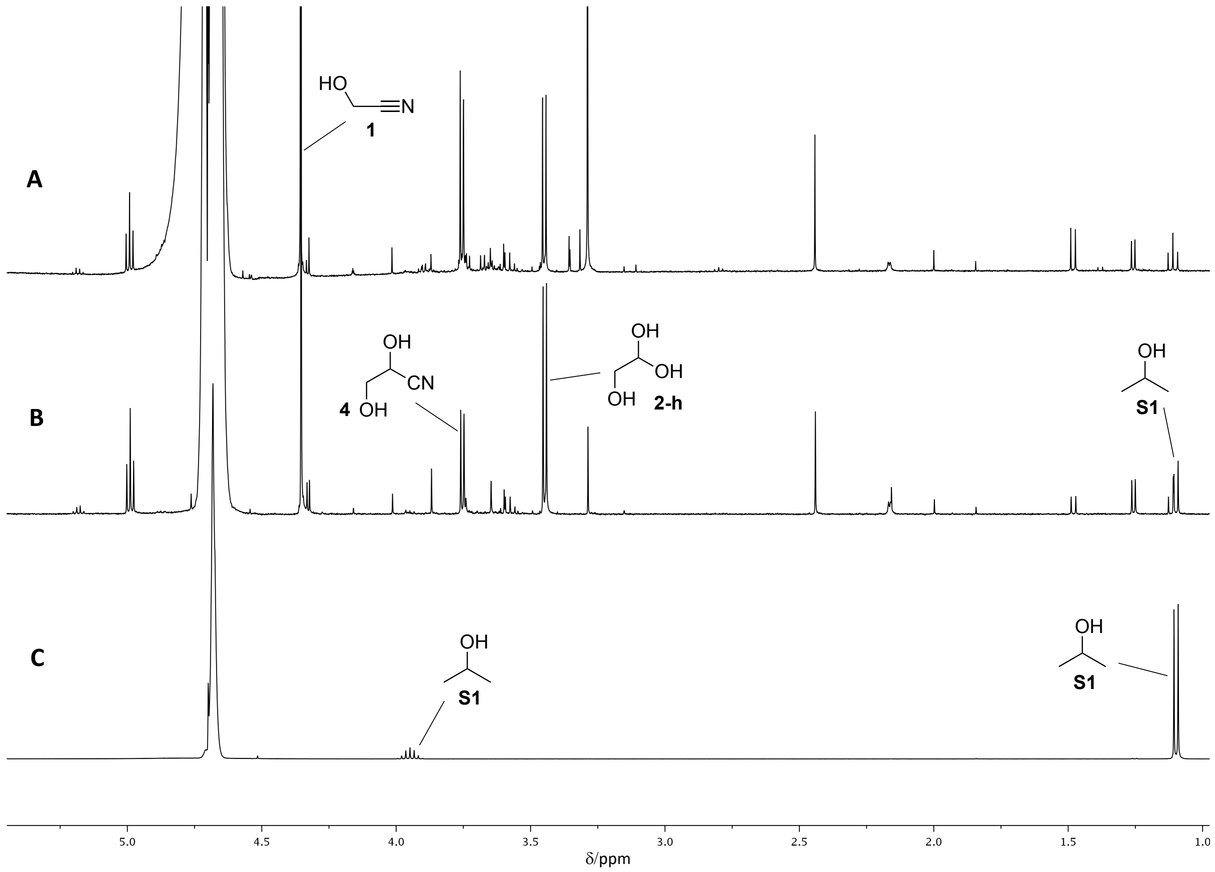

Supplementary Fig. 3 Photochemical reduction of commercial glycolonitrile **1** or **1** formed *in situ*. A – Formaldehyde ~ 37% (0.040 mmol, 3.0 μL) and KCN (0.043 mmol, 2.8 mg) were dissolved in degassed 10% D_2O in H_2O (1 mL) and Na_3PSO_3 (1 equiv.) was added. The pH was adjusted to 6.5, volume made up to 2 mL with degassed 10% D_2O in H_2O then the solution was irradiated for 1 h before a ^1H NMR spectrum (spectrum A) of the crude reaction was acquired; B – ^1H NMR Spectrum of the reaction according to Procedure 1 (1 equiv. Na_3PSO_3) after 1 h irradiation; C – Acetone (0.020 mmol, 1.5 μL) and Na_3PSO_3 (0.040 mol, 8 mg) were dissolved in degassed 10% D_2O in H_2O (1 mL) and Na_3PSO_3 (1 equiv.) was added. The pH was adjusted to 6.5 and the volume made up to 2 mL with degassed 10% D_2O in H_2O before being irradiated for 1 h. A ^1H NMR spectrum of the crude reaction (spectrum C) was then acquired. In the reduction of **1** which was made 'in house' (spectrum A) no isopropanol **S1** was observed after 1 h reaction (large singlet at 3.30 ppm is due to MeOH in commercial formaldehyde) whereas in the reduction of the commercial sample which contained acetone (spectrum B) the characteristic doublet of **S1** (1.10 ppm) is present. The photochemical reduction of acetone (spectrum C) using Na_3PSO_3 is quantitative, giving **S1** as the sole product.

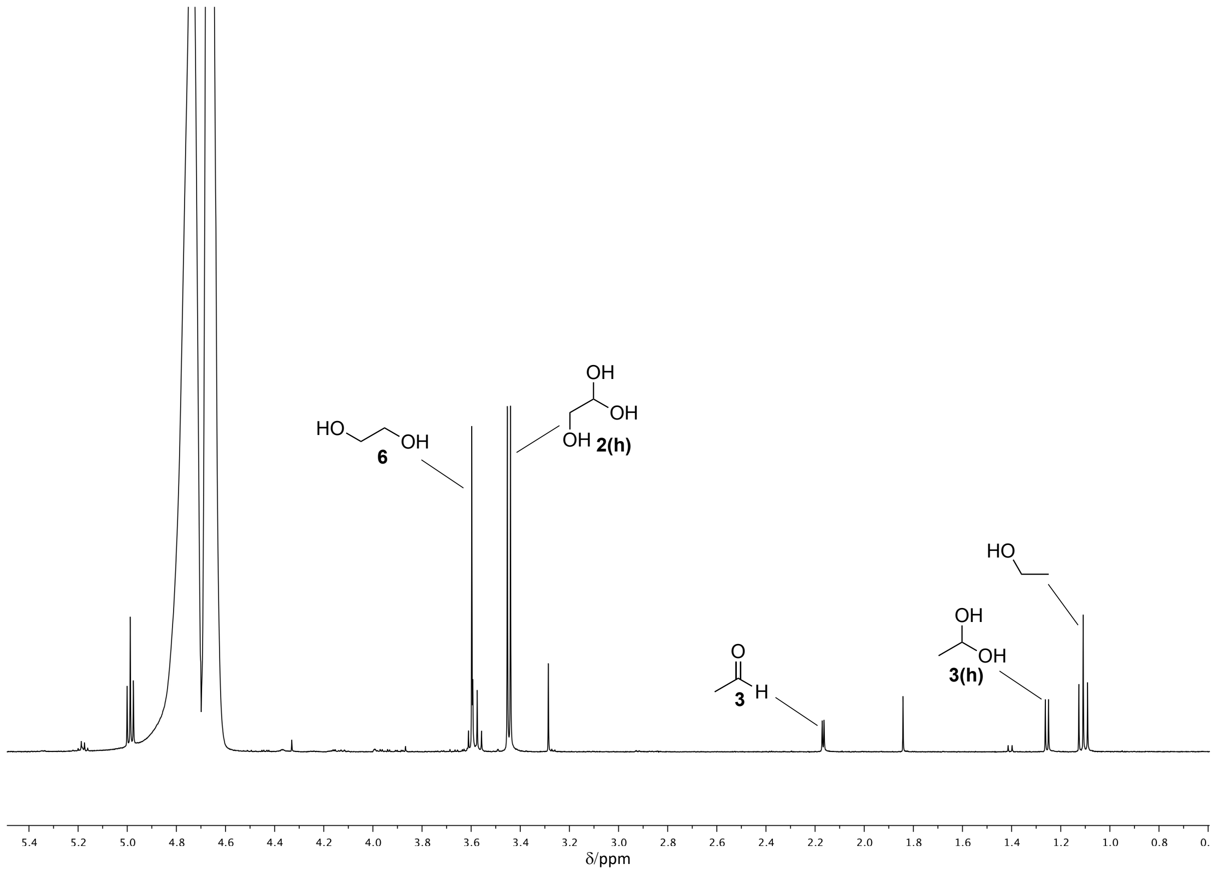

Supplementary Fig. 4 Photochemical reduction of glycolaldehyde **2** with thiophosphate gives acetaldehyde **3**. A solution of Na_3PSO_3 and **2** in degassed 10% D_2O in H_2O was adjusted to pH 7.0 and the volume adjusted using degassed 10% D_2O in H_2O to give the appropriate concentrations of Na_3PSO_3 (10 mM) and **2** (15 mM). The solution was transferred to a quartz cuvette and irradiated for 30 min, the above ^{1}H NMR spectrum was then acquired from the crude reaction mixture.

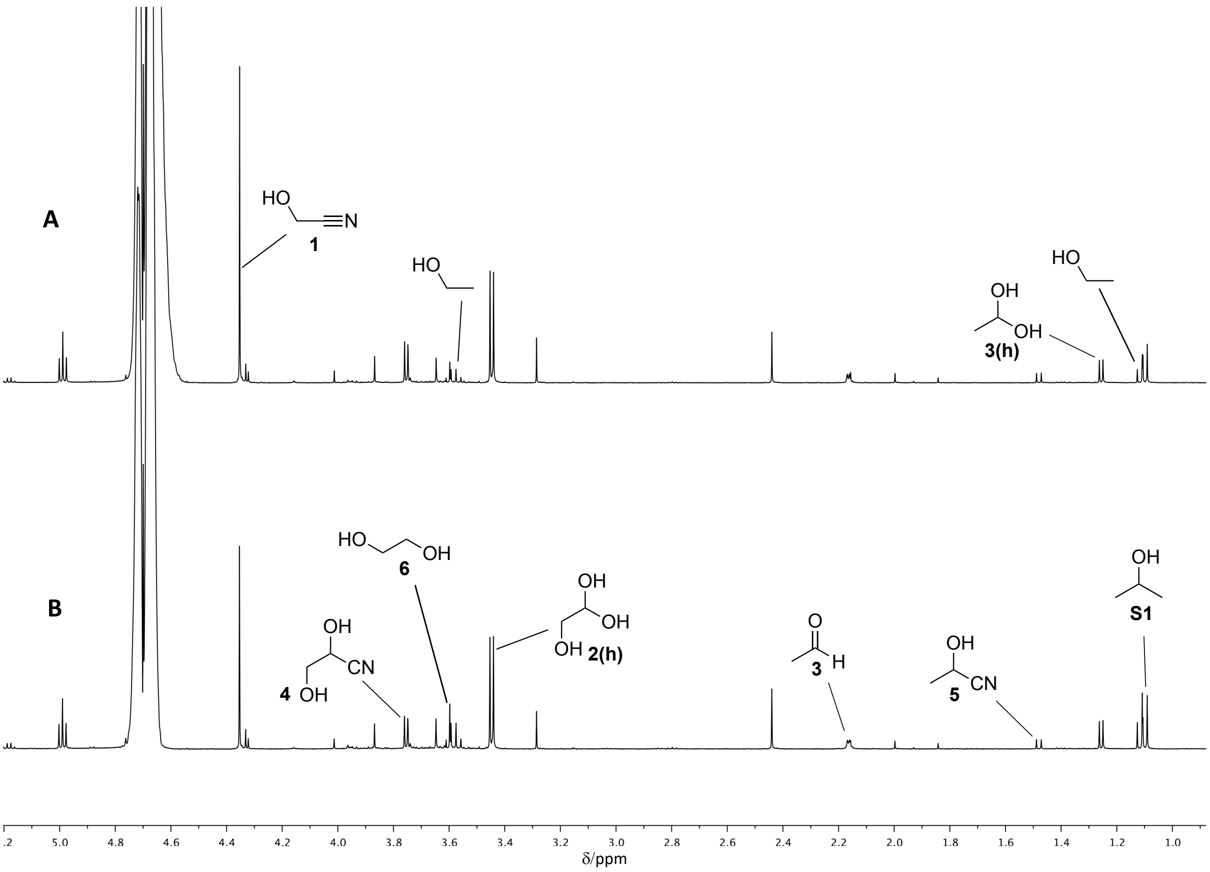

Supplementary Fig. 5 Extended photochemical reduction of glycolonitrile **1** using thiophosphate. A – ^{1}H NMR Spectrum of the reaction according to Procedure 1 (1 equiv. Na_3PSO_3) after 2 h irradiation *cf.* Supplementary Fig. 2, spectrum A. In this spectrum, glycolaldehyde **2** was present in ~ 20% yield, glyceronitrile **4** in ~ 8% yield, acetaldehyde **3** (hydrate and aldehyde) in ~ 5% yield, lactonitrile **5** in ~ 1% yield, ethylene glycol **6** in ~ 1% yield and EtOH in ~ 3% yield; B – ^{1}H NMR Spectrum of the reaction according to Procedure 1 (1.5 equiv. Na_3PSO_3) after 2 h irradiation, *cf.* Supplementary Fig. 2, spectrum B. In this spectrum **2** had been produced in ~ 22% yield, **4** in ~ 7% yield, **3** in ~ 6% yield, **5** in ~ 1% yield, **6** in ~ 2% yield and EtOH in ~ 6% yield (*cf.* ~ 2% yield of EtOH after 1 h irradiation, see main text, Supplementary Fig. 2 and Supplementary Table 1). The signals at 3.30 ppm and 2.44 ppm are due to MeOH and thioacetamide, respectively (although thioacetamide can be further reduced to acetaldehyde **3** the reduction of thioamides takes place much more slowly than cyanohydrins (see Supplementary Fig. 48), hence we believe the production of **3** comes mainly from deoxygenation of **2**, see Supplementary Fig. 4). Yields estimated by addition of disodium fumarate (50 mM solution, 50 µL into 450 µL of crude reaction) as a standard and relative integration of ^{1}H NMR signals.

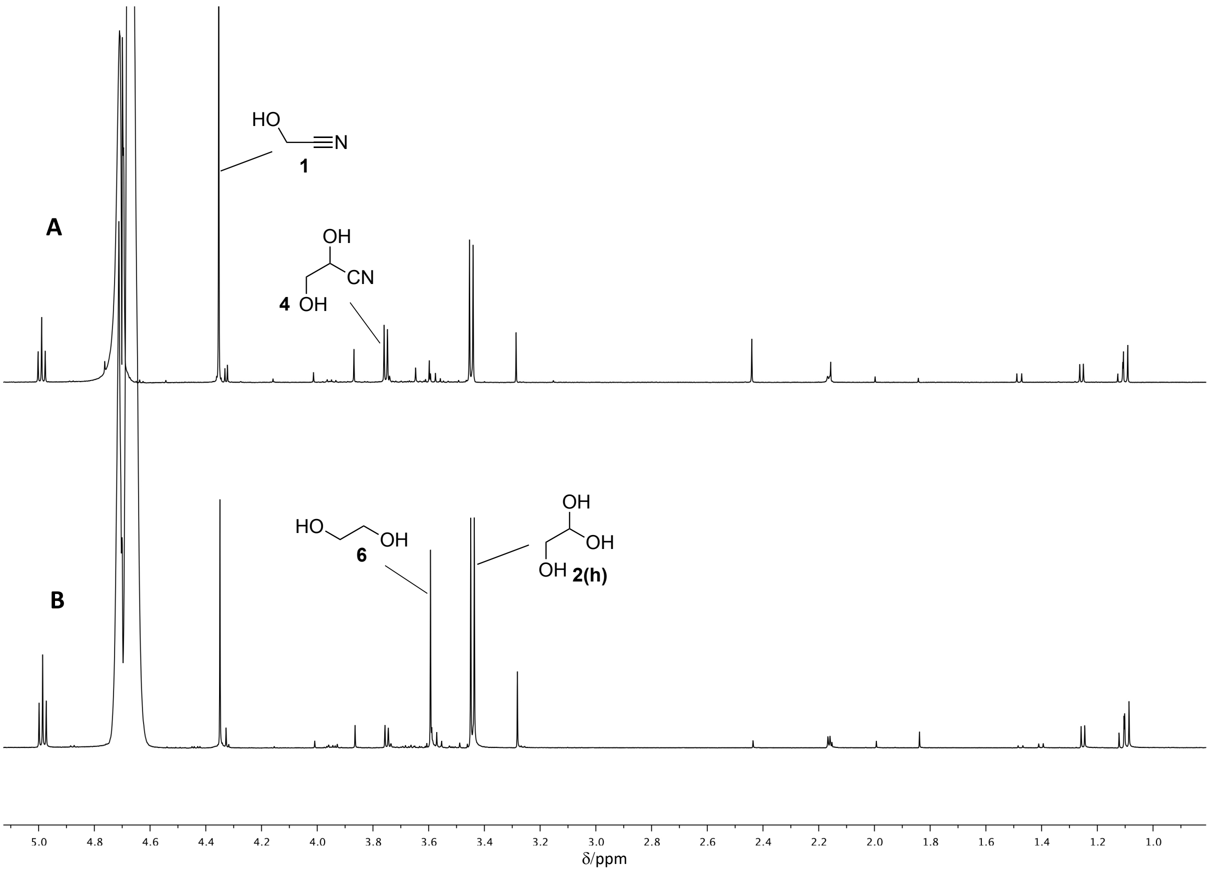

Supplementary Fig. 6 Reduction of glycolonitrile **1** in the presence and absence of PO_4^{3-}. A – As Supplementary Fig. 2, Spectrum A (^{1}H NMR Spectrum of the reaction mixture after 1 h irradiation following Procedure 1); B – As Spectrum A but with the inclusion of NaH_2PO_4 (20 mM). Interestingly, in the presence of phosphate buffer (spectrum B) formation of thioacetamide (singlet at 2.42 ppm) was suppressed. Yields estimated by addition of disodium fumarate (50 mM solution, 50 μL into 450 μL of crude reaction) as a standard and relative integration of ^{1}H NMR signals – see Supplementary Table 1.

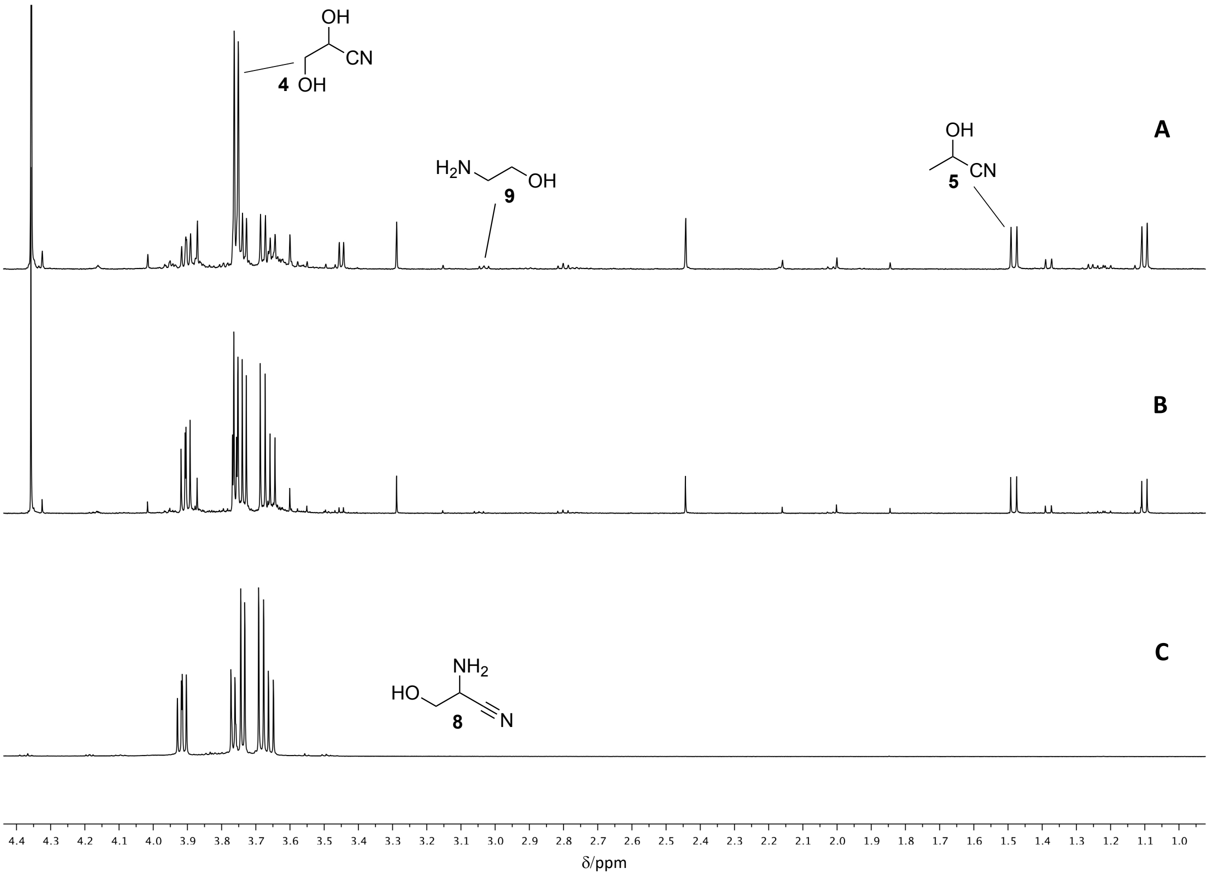

Supplementary Fig. 7 Attempted one pot synthesis of glyceraldehyde **7** from glycolonitrile **1**. A – ^{1}H NMR Spectrum of the crude reaction mixture after 1 h reaction according to Procedure 1 using 2 equivalents of Na_3PSO_3 and with the addition of KCN (0.040 mmol, 2.6 mg, 1 equiv.), initial pH of the reaction was 6.5. Yields estimated by addition of disodium fumarate (50 mM solution, 50 μL into 450 μL of crude reaction) as a standard and relative integration of ^{1}H NMR signals (see Supplementary Tables 2.1 and 2.2); B – As spectrum A, spiked with serine nitrile **8**, made as follows: NH_4Cl (54 mg, 1.00 mmol) was dissolved in H_2O (0.7 mL) then glycolaldehyde **2** (6 mg, 0.100 mmol) and KCN (8 mg, 0.120 mmol) were added. The pH was adjusted to 9.1 and D_2O (0.1 mL) added and volume adjusted to 1.0 mL with H_2O. The solution was left at room temperature for 4 days; C – ^{1}H NMR Spectrum of **8**.

Supplementary Table 2.1 Photoreductive homologation of glycolonitrile **1** and HCN or HCN alone.

Entry	Spectrum	Glycolonitrile **1**	PSO_3^{3-}	KCN	PO_4^{3-}	Irradiation time	See Table 2.2
+	Supp. Fig. 7, Spectrum A	20 mM	40 mM	20 mM	–	1 h	Entry 1
2	Supp. Fig. 8, Spectrum A	15 mM	55 mM	20 mM	60 mM	1 h	Entry 2
3	Supp. Fig. 10, Spectrum A	–	20 mM	50 mM	50 mM	1.25 h	Entry 3

Supplementary Table 2.2

	Yields of products[a]							
Entry	Glycolaldehyde **2**	Glyceronitrile **4**	Lactonitrile **5**	Serine nitrile **8**	Glyceraldehyde **7**	Glyceraldehyde cyanohydrin **10**	Glycerol **11**	Ethylene glycol **6**
1	~4%	~24%	~3%	~16%	–	–	–	–
2	~6%	~9%	~4%	–	~5%	~13%	~6%	~6%
3	–	~6%	trace	~3%	–	~5%	trace	trace

[a] - yields based on carbon feedstock(s)
trace - a yield of 1% or less

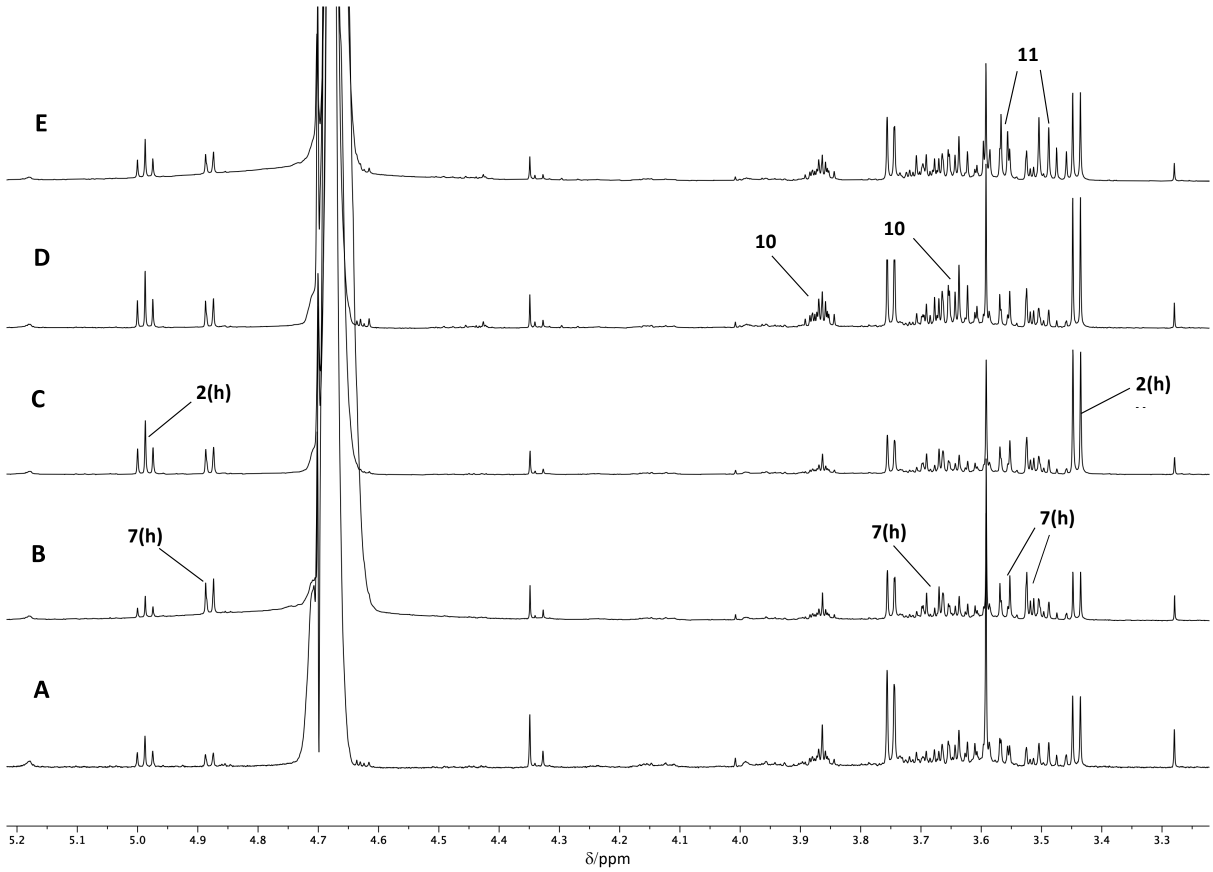

Supplementary Fig. 8 One pot synthesis of glyceraldehyde **7** from glycolonitrile **1** and HCN. A – A solution of KCN (0.040 mmol, 2.6 mg), $NaH_2PO_4.2H_2O$ (0.120 mmol, 19 mg) and Na_3PSO_3 (the purity and water content were predetermined and accounted for, 0.110 mmol) were dissolved in degassed 10% D_2O in H_2O (1 mL) and pH adjusted to 6.5. Glycolonitrile **1** (0.030 mmol, 3.0 μL) was added, the volume made up to 2 mL with degassed 10% D_2O in H_2O then the solution irradiated. After 1 h a ^{1}H NMR spectrum of the crude reaction mixture (spectrum A) was acquired; B – As spectrum A, spiked with a commercial sample of **7**; C – As spectrum B, spiked with a commercial sample of glycolaldehyde **2**; D – As spectrum C, spiked with an authentic sample of glyceraldehyde cyanohydrin **10**; E – As spectrum D, spiked with a commercial sample of glycerol **11**. Yields estimated by addition of disodium fumarate (50 mM solution, 50 μL into 450 μL of crude reaction) as a standard and relative integration of ^{1}H NMR signals. Yields based on maximum product concentration possible from HCN + **1**, due to the fact that HCN can also be reduced and homologated to **1**, thus the maximum concentration of C_2 and C_3 compounds are 25 mM and 16.7 mM, respectively.

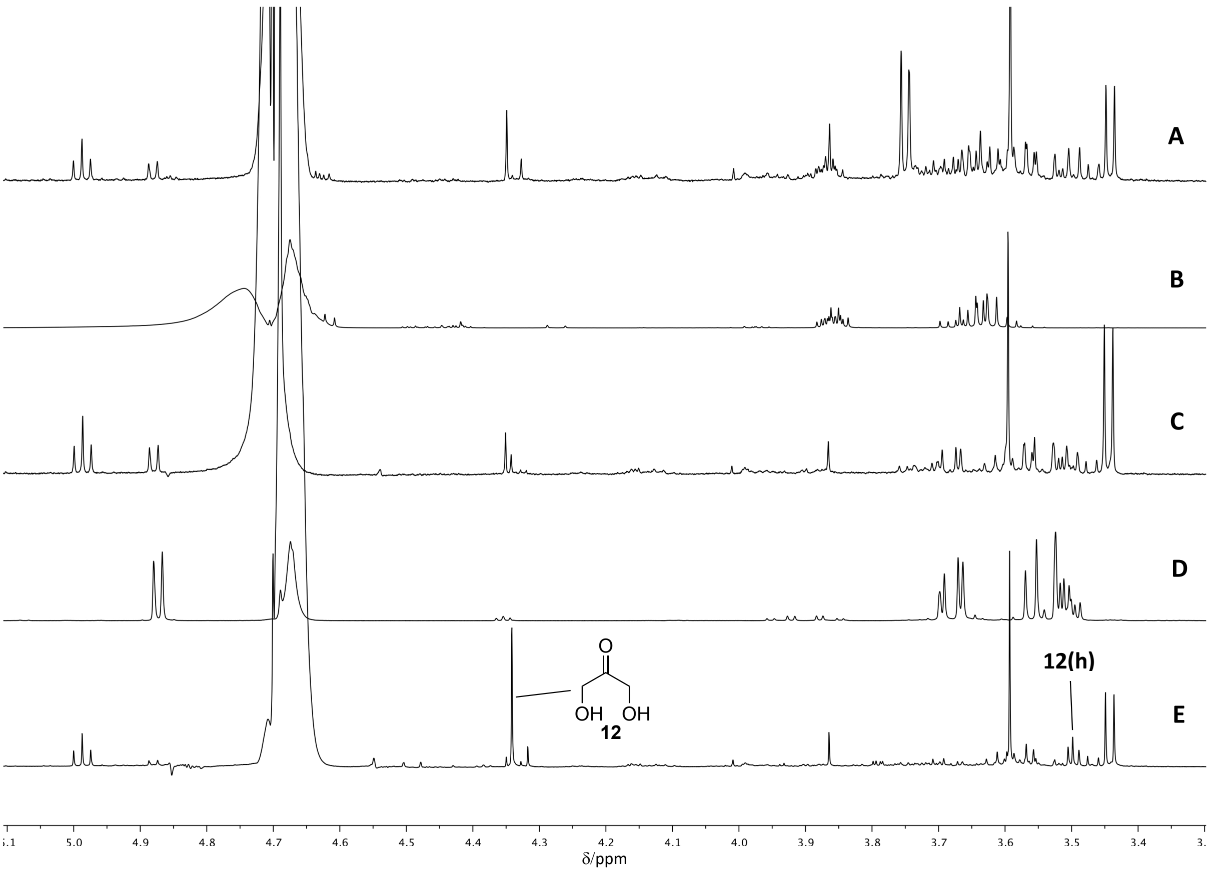

Supplementary Fig. 9 One pot synthesis of glyceraldehyde **7** from glycolonitrile **1** and HCN and conversion to dihydroxyacetone **12**. A – As Supplementary Fig. 8, spectrum A; B – ^{1}H NMR Spectrum of an authentic sample of glyceraldehyde cyanohydrin **10**; C – To a portion (1.5 mL) of the reaction mixture from which spectrum A was taken, was added H_2O (4.5 mL) and N_2 bubbled gently through the solution for 48 h (final volume ~ 2 mL). A ^{1}H NMR Spectrum (spectrum C) was then acquired; D – ^{1}H NMR Spectrum of a commercial sample of **7**; E – As spectrum D after being left for 2 weeks at room temperature. Although there appears to be far more dihydroxyacetone **12** than **7** in spectrum E, the signal at 4.88 ppm (doublet) from **7** is attenuated by the NMR experiment which suppresses the H_2O/HOD signal. The yield was calculated by addition of an external standard and relative integration of the ^{1}H NMR signals (in the case of **7(h)**, the signals at 3.67 ppm gave clear integration). Yields based on maximum product concentration possible from HCN + **1**, due to the fact that HCN can also be reduced and homologated to **1**, thus the maximum concentration of C_2 and C_3 compounds are 25 mM and 16.7 mM, respectively.

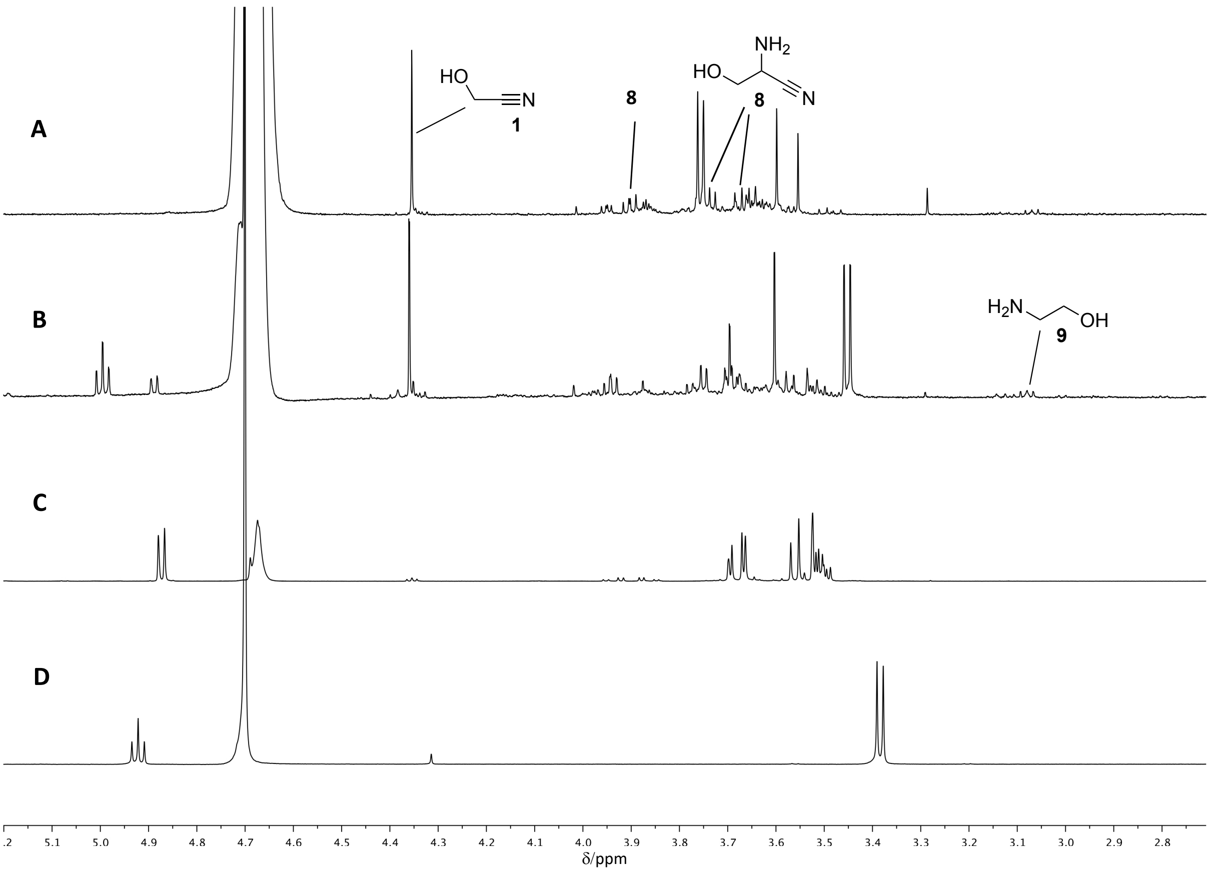

Supplementary Fig. 10 One pot synthesis of glyceraldehyde **7** from HCN. A solution of KCN (7 mg, 0.100 mmol), $Na_3PSO_3.xH_2O$ (the purity and water content were predetermined and accounted for, 0.040 mmol) and $NaH_2PO_4.2H_2O$ (16 mg, 0.100 mmol) in degassed 10% D_2O in H_2O (2 mL) at pH 6.5 were irradiated for 1.25 h and a ^{1}H NMR spectrum of the crude reaction was acquired. To assist identification of **7**, the remaining 1.55 mL of the crude reaction was adjusted to pH 7.0, diluted to 8 mL with H_2O and N_2 bubbled gently through the solution for 4 d (final volume ~ 1 mL) before the crude reaction examined by ^{1}H NMR spectroscopy (spectrum B). C – A ^{1}H NMR spectrum of a commercial sample of **7**; D – A ^{1}H NMR spectrum of a commercial sample of glycolaldehyde **2**. In spectrum A there was ~ 6% of glyceronitrile **4**, ~ 3% of serine nitrile **8**, ~ 5% of glyceraldehyde cyanohydrin **10**, based on HCN. The maximum possible yield of **2** or **7** is equivalent to half, or one third, the molarity of the initial HCN concentration, respectively. Yields estimated by addition of disodium fumarate (50 mM solution, 50 μL into 450 μL of crude reaction) as a standard and relative integration of ^{1}H NMR signals.

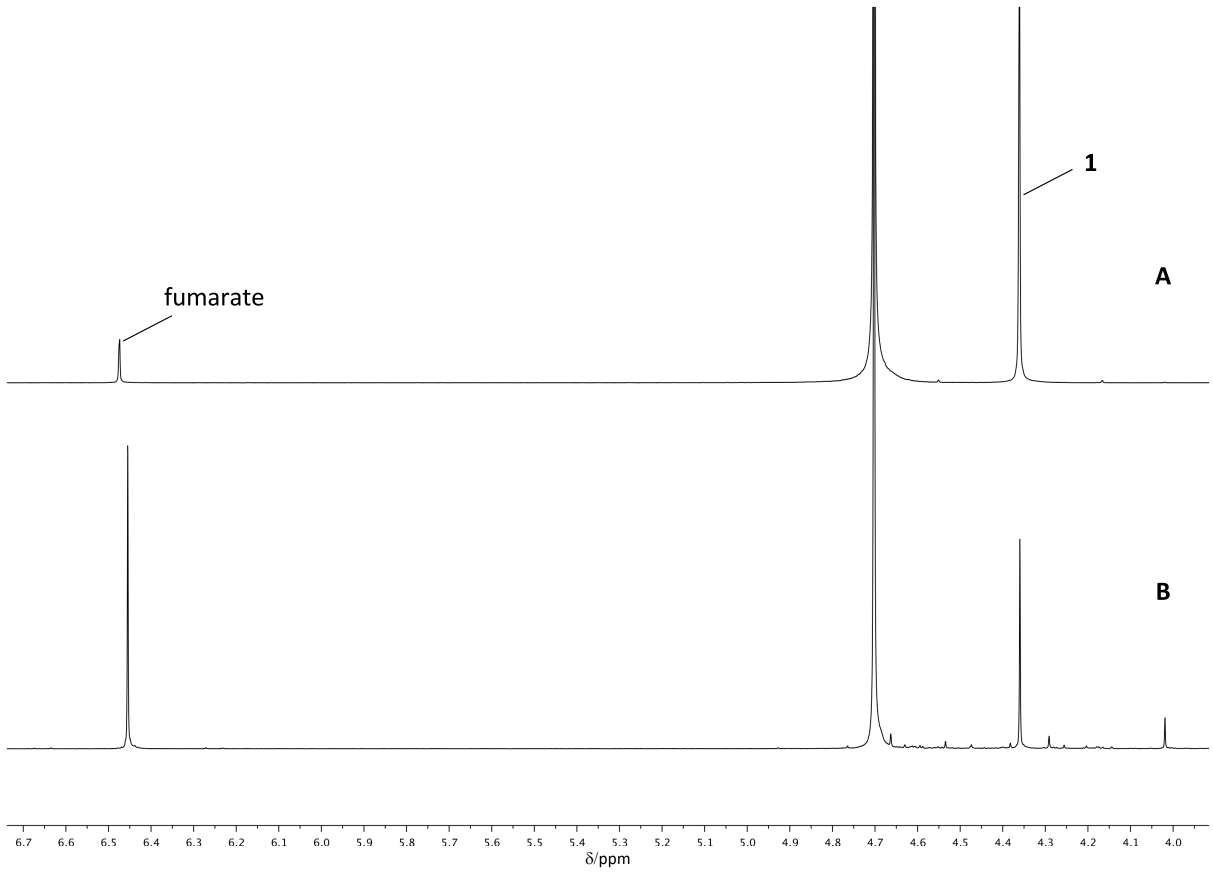

Supplementary Fig. 11 Retention of glycolonitrile **1** after evaporation. To a solution of **1** (55% wt., 20.0 μL, 0.100 mmol) in D_2O was added disodium fumarate solution (50 mM, 200 μL) and the pD was altered to ~ 7, then a ^1H NMR spectrum (spectrum A) was acquired without solvent suppression. The volume was adjusted to 1 mL then N_2 was bubbled through the solution at ambient temperature overnight until a solid residue remained. The residue was dissolved in D_2O and a ^1H NMR spectrum of the crude mixture (spectrum B) was acquired. In spectrum A, integration of fumarate:**1** is 1:7.7. In spectrum B, integration of fumarate:**1** is 1:0.8, ~ 10% of **1** remains. Also see Supplementary Fig. 10, spectrum B, where ~ 90% of H_2O had been evaporated, but **1** remained in approximately the same concentration as before evaporation of H_2O.

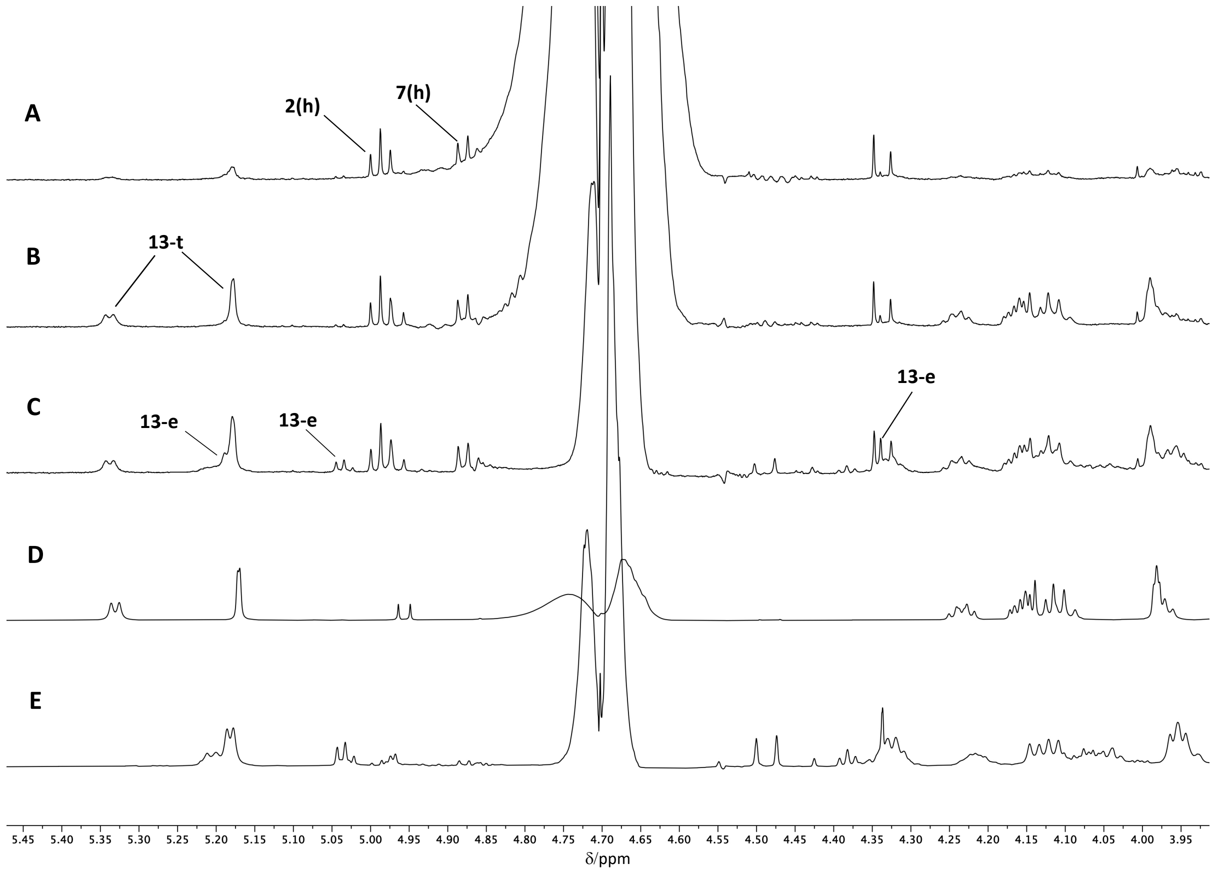

Supplementary Fig. 12 One pot synthesis of the tetroses **13** from glycolonitrile **1** and HCN. A – A solution of KCN (0.040 mmol, 2.6 mg), $NaH_2PO_4.2H_2O$ (0.120 mmol, 19 mg) and Na_3PSO_3 (the purity and water content were predetermined and accounted for, 0.110 mmol) were dissolved in degassed 10% D_2O in H_2O (1 mL) and pH adjusted to 6.5. Glycolonitrile **1** (0.030 mmol, 3.0 μL) was added, the volume made up to 2 mL with degassed 10% D_2O in H_2O then the solution irradiated. After 1 h, a ^1H NMR spectrum of the crude reaction mixture (spectrum A) was acquired; B – As spectrum A, spiked with a commercial sample of threose **13-t**; C – As spectrum B, spiked with a commercial sample of erythrose **13-e**; D – ^1H NMR Spectrum of a commercial sample of **13-t** in phosphate buffer at pH 7.0; E – ^1H NMR Spectrum of a commercial sample of **13-e** in phosphate buffer at pH 7.0 (a pure commercial sample of **13-e** could not be obtained, hence the extraneous signals observed in spectrum E). To quantify the C_2 – C_4 sugar products, the reaction was repeated and oxime derivatives made to assist integration, see Supplementary Fig. 13.

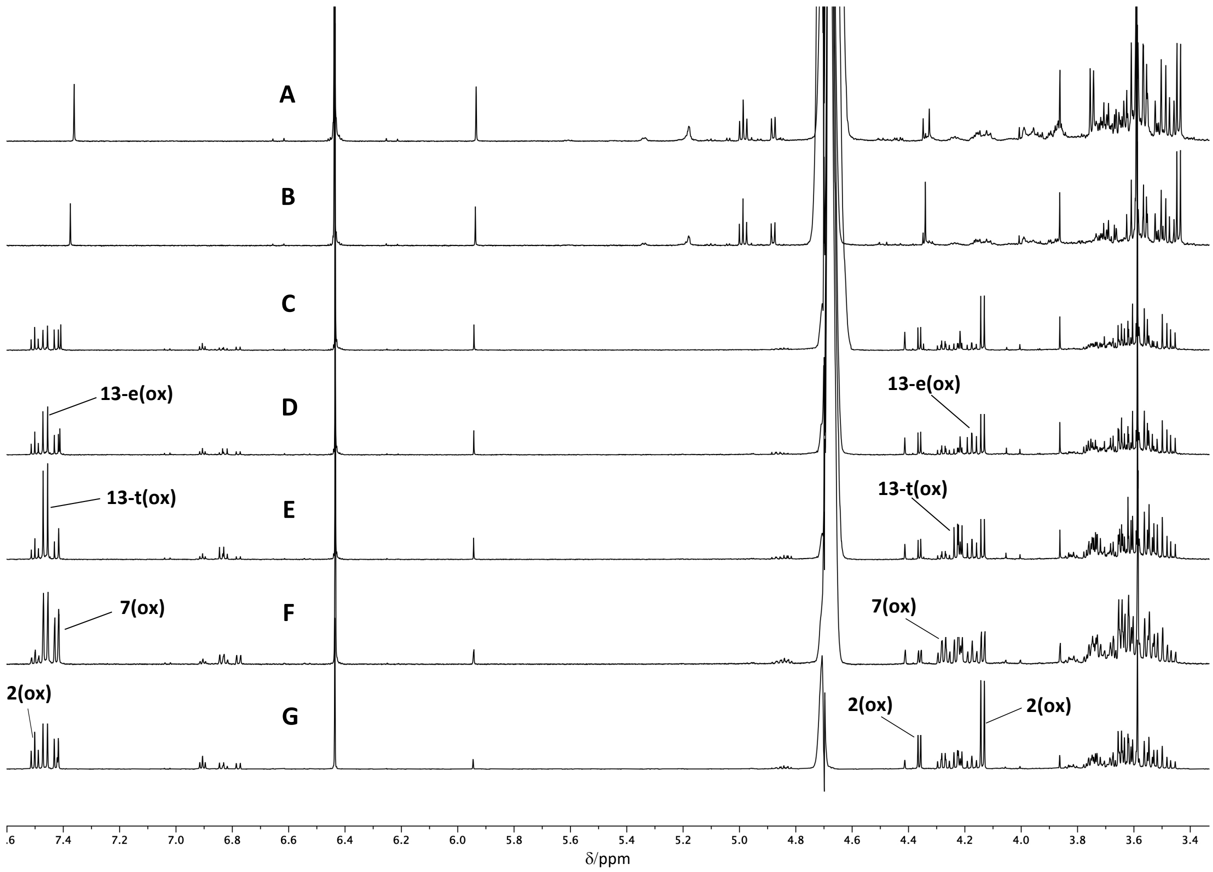

Supplementary Fig. 13 Identification and quantitation of the C_2, C_3 and C_4 sugars made from glycolonitrile **1** and HCN in one pot. A – A solution of KCN (0.040 mmol, 2.6 mg), $NaH_2PO_4.2H_2O$ (0.120 mmol, 19 mg) and Na_3PSO_3 (the purity and water content were predetermined and accounted for, 0.110 mmol) were dissolved in degassed 10% D_2O in H_2O (1 mL) and pH adjusted to 6.5. Glycolonitrile **1** (0.030 mmol, 3.0 μL) was added, the volume made up to 2 mL with degassed 10% D_2O in H_2O then the solution irradiated. After 1 h a ^1H NMR spectrum of the crude reaction mixture (spectrum A) was acquired; B – To a portion (900 μL) of the crude reaction mixture was added fumarate solution (pH 7, 50 mM, 100 μL) and H_2O (8 mL), then N_2 was bubbled through the solution for 3 d and spectrum B obtained. This was done in order to remove HCN from the system and allow clean oxime formation; C – To the NMR sample from B was added NH_2OH solution (2 M, pH 6.2, 100 μL) and spectrum C obtained; D – As spectrum C, after the addition of an authentic standard of erythrose oxime **13-e(ox)**; E – As spectrum D, after the addition of an authentic standard of threose oxime **13-t(ox)**; F – As spectrum E, after the addition of an authentic standard of glyceraldehyde oxime **7(ox)**; G – As spectrum F, after the addition of an authentic standard of glycolaldehyde oxime **2(ox)**. The singlet at 6.4 ppm in all spectra is due to fumarate, added as an external standard. Assuming no loss of sugars during the removal of HCN, the yields of sugars in spectrum A was ~ 8% glycolaldehyde **2**, ~ 8% glyceraldehyde **7** and ~ 19% tetroses **13**. Although there is a higher concentration of **2**, and **7** and **13** are present in similar concentrations, the maximum concentration of each product varies due to the fact that HCN can also be reduced and homologated to **1**, thus the maximum concentration of **2**, **7** and **13**, is 25 mM, 16.7 mM and

10 mM, respectively. Erythrose **13-e** and threose **13-t** were formed in 0.9:1 ratio, as determined by the integration of the C2-H signals (4.15 – 4.24 ppm). In spectrum A, there was ~ 4% C_1 (MeOH + formate), ~ 27% C_2 (glycolaldehyde **2**, glyceronitrile **4**, ethanolamine **9**, ethylene glycol **6**, acetaldehyde **3** and its addition products **3(h)**, **3(t)** and **5**, MeCN, acetate and EtOH), ~ 22% C_3 (glyceraldehyde **7** (~ 8%, this was calculated from Supplementary Fig. 13, spectrum C, as the C2-H signal of glyceraldehyde cyanohydrin **10** did not give a clear integration), glyceraldehyde cyanohydrin **10**, glycerol **11** and 1,3-dihydroxypropane) and ~ 19% C_4 compounds (tetroses **13** and their cyanohydrins **15**. By comparison to Spectrum C, which gave good resolution of the NMR spectroscopy signals and a yield of ~ 19% of **13**, there was ~ 4% of the tetrose cyanohydrins **15** in spectrum A *i.e.* ~ 15% of **13**. The reduced tetroses (erythritol and threitol), if present, could not be observed in the crude reaction mixture by routine ^{1}H NMR spectroscopy).

Supplementary Table 3.1 Kiliani-Fischer-like homologation to higher sugars from glycolonitrile **1** and HCN or HCN alone

Entry	Spectrum	Glycolonitrile **1**	PSO_3^{3-}	KCN	PO_4^{3-}	Irradiation time	See Table 3.2
1	Supp. Fig. 13, Spectrum A	15 mM	55 mM	20 mM	60	1 h	Entry 1
2	Supp. Fig. 15, Spectrum A	7.5 mM	100 mM	45 mM	75 mM	2.75 h	Entry 2
3	Supp. Fig. 16, Spectrum A	–	100 mM	55 mM	75 mM	3 h	Entry 3

Supplementary Table 3.2

	Yields of products[a]								
Entry	Glycolaldehyde **2**	Glyceraldehyde **7**	Tetroses **13**	Pentoses **14**	Glycerol **11**	Ethylene glycol **6**	EtOH	1,3-Dihydroxypropane	Ethanolamine **9**
1[b]	~8%	~8%	~19%[c]	–	~12%	~8%	~3%	~4%	~2%
2[d]	~2%	~2%	~11%	~29%[e]	~7%	~4%	~3%	~3%	~2%
3[f]	~1%	~2%	~5%	~19%	~3%	~2%	trace	~2%	trace

[a] - yields based on carbon feedstock(s)

[b] - yields actually derived from Supplementary Fig. 13, Spectrum C *i.e.* after oxime formation, except EtOH, the yield of which came from Supplementary Fig. 13, Spectrum A

[c] - diastereomeric ratio of threose **13-t**:erythrose **13-e** ~ 0.9:1.0

[d] - yields actually derived from Supplementary Fig. 15, Spectrum B *i.e.* after removal of HCN to simplify quantification, except EtOH, the yield of which came from Supplementary Fig. 15, Spectrum A

[e] - diastereomeric ratio of ribose **14-r**, lyxose **14-l**, arabinose **14-a** and xylose **14-x** ~ 1:1.2:1.2:1

[f] - yields actually derived from Supplementary Fig. 16, Spectrum H *i.e.* after removal of HCN to simplify quantification, except EtOH and **9**, the yield of which came from Supplementary Fig. 16, Spectrum A

trace - yield of 1% or less

Supplementary Table 4 Summary of products.

Compound class	Products	Combined yield
C_1	MeOH<br>Formate	~4%
C_2	Glycolaldehyde **2**<br>Glyceronitrile **4**<br>Ethanolamine **9**<br>Ethylene glycol **6**<br>Acetaldehyde **3**<br>Acetaldehyde hydrate **3(h)**<br>Acetaldehyde thiohydrate **3(t)**<br>Lactonitrile **5**<br>MeCN<br>EtOH<br>AcO^-	~27%
C_3	Glyceraldehyde **7**<br>Glyceraldehyde cyanohydrin **10**<br>Glycerol **11**<br>1,3-Dihydroxypropane	~22%
C_4	Tetroses **13**<br>Tetrose cyanohydrins **15**	~19%

Summary of the products identified in each compound class (C_1-C_4) in Supplementary Fig. 13, spectrum A, and the total yield for all compounds identified in each class. Also see Supplementary Fig. 13, caption. Cyanohydrins were counted in the same class of compounds as the parent aldehyde *e.g.* glyceronitrile **4** was counted as a C_2 compound. This was to avoid misrepresentation of the overall yields for each stage of reduction *i.e.* an aldehyde belonging to the class of compounds C_n is converted to C_{n+1} when cyanide adds *in situ*, artificially depleting the yield of C_n and inflating the yield of C_{n+1} compounds. Glycolonitrile **1** was not included in the products, as it was not possible to know how much was synthesised *in situ* from HCN. Yields based on **1** and HCN.

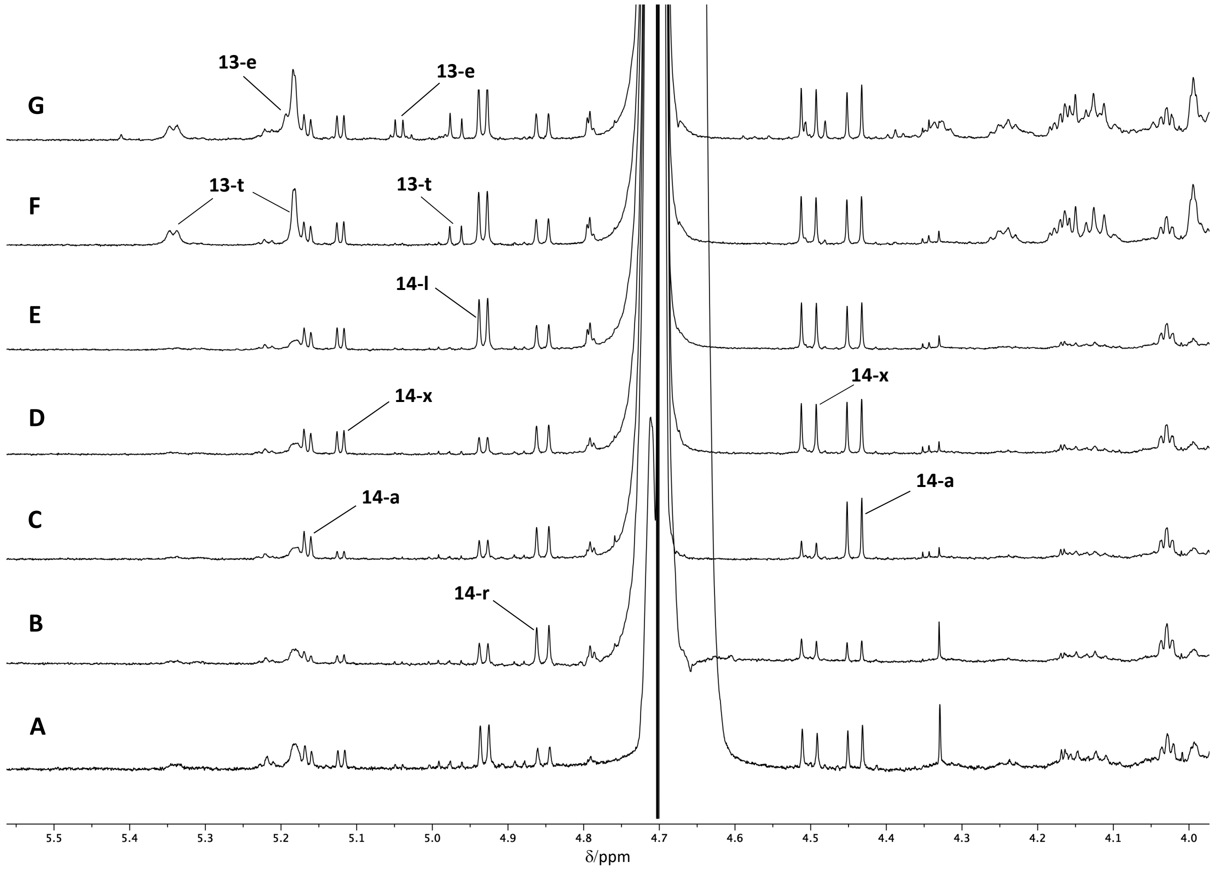

Supplementary Fig. 14 Identification of C_4 and C_5 sugars made from glycolonitrile **1** and HCN in one pot. A – A solution of KCN (0.090 mmol, 6 mg), $NaH_2PO_4.2H_2O$ (0.150 mmol, 23 mg) and Na_3PSO_3 (the purity and water content were predetermined and accounted for, 0.200 mmol) were dissolved in degassed 10% D_2O in H_2O (1 mL) and pH adjusted to 6.5. Glycolonitrile **1** (0.015 mmol, 3.0 μL) was added, the volume made up to 2 mL with degassed 10% D_2O in H_2O then the solution was irradiated. After 2.5 h, a ^1H NMR spectrum of the crude reaction mixture (spectrum A) was acquired; B – As spectrum A, spiked with a commercial sample of ribose **14-r**; C – As spectrum B, spiked with a commercial sample of arabinose **14-a**; D – As spectrum C, spiked with a commercial sample of xylose **14-x**; E – As spectrum D, spiked with a commercial sample of lyxose **14-a**; F – As spectrum E, spiked with a commercial sample of threose **13-t**; G – As spectrum F, spiked with a commercial sample of erythrose **13-e**.

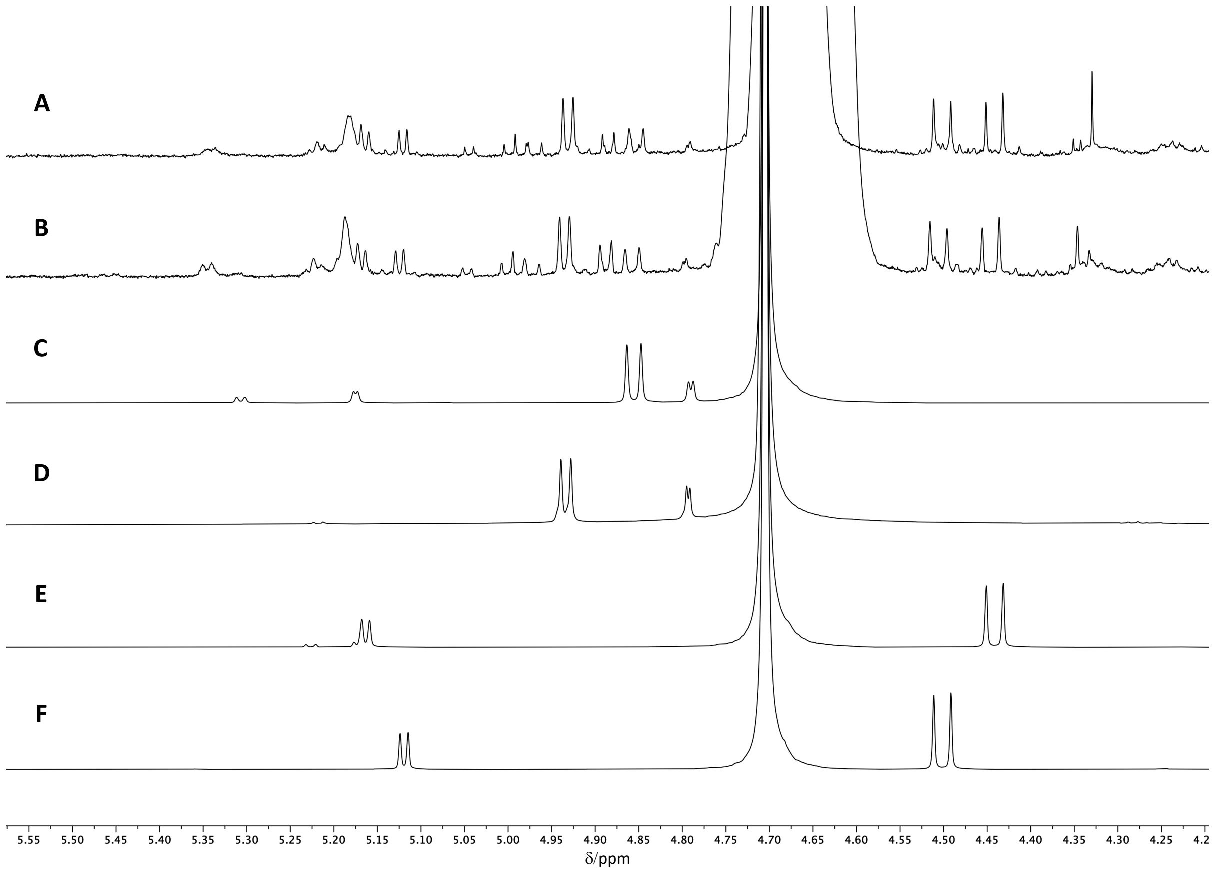

Supplementary Fig. 15 Quantification of C_5 sugars made from glycolonitrile **1** and HCN in one pot. A – A solution of KCN (0.090 mmol, 6 mg), $NaH_2PO_4.2H_2O$ (0.150 mmol, 23 mg) and Na_3PSO_3 (the purity and water content were predetermined and accounted for, 0.200 mmol) were dissolved in degassed 10% D_2O in H_2O (1 mL) and pH adjusted to 6.5. Glycolonitrile **1** (0.015 mmol, 3.0 μL) was added, the volume made up to 2 mL with degassed 10% D_2O in H_2O then the solution irradiated. After 2.75 h a ^1H NMR spectrum of the crude reaction mixture (spectrum A) was acquired with fumarate added as a standard; B – To an aliquot (900 μL) of the crude reaction mixture was added fumarate (100 μL, 50 mM), as a standard, and H_2O (7 mL), then excess HCN was removed by bubbling N_2 through the solution for 2 d, after which the volume was ~ 0.5 mL. A ^1H NMR spectrum of the solution (spectrum B) was then acquired. The yields of **2**, **7**, **13** and **14** in spectrum B were ~ 2%, 2%, 11% and 29%, respectively. Ribose **14-r**, lyxose **14-l**, arabinose **14-a** and xylose **14-x** were present in a ratio of ~ 1:1.2:1.2:1. Due to signals from **14-r** and **14-l** (~ 4.78 ppm) being in close proximity to the H_2O/HOD peak, and that the solvent suppression NMR experiment supressed these signals significantly, coupled with signal overlap between **14-r** and the tetroses **13** (5.17 ppm), the yields of **14-r** and **14-l** were calculated using the peak corresponding to the major cyclic form of each sugar and its relative abundance to the minor forms of each sugar, as observed and measured for the pure sugars in phosphate buffer (pD 7, Supplementary Fig. 15, spectra C and D). The calculated integration for the peak of **14-r** at 5.17 ppm could then be deducted from the yield of **13**. An added complication in the calculation of these yields comes from the fact that HCN can be reduced under the reaction conditions, and after hydrolysis and addition of further HCN,

generates the starting material, glycolonitrile **1**. Thus, the maximum possible concentration of **2**, **7**, **13** and **14** would be 30 mM, 20 mM, 15 mM and 12 mM, respectively, and consequently the yields of each product were calculated from these values. In spectrum A, there was ~ 1% C_1 (MeOH + formate), ~ 8% C_2 (glycolaldehyde **2**, ethanolamine **9**, ethylene glycol **6**, MeCN, acetate and EtOH), ~ 11% C_3 (glyceraldehyde **6**, glycerol **11** and 1,3-dihydroxypropane), ~ 11% C_4 (tetroses **13**) and ~ 29% C_5 compounds (pentoses **14**). Glycolaldehyde **2** and glyceraldehyde **7** yields were calculated from spectrum B, as integration of their cyanohydrins in spectrum A was not possible. As solvent suppression was used to acquire the ^{1}H NMR spectra, these yields are thought to be conservative due to some attenuation of the signals most proximal to the HOD/H_2O peak; C – ^{1}H NMR spectrum of an authentic sample of ribose and NaD_2PO_4 in D_2O at pD 7.0; D – ^{1}H NMR spectrum of an authentic sample of lyxose and NaD_2PO_4 in D_2O at pD 7.0; E – ^{1}H NMR spectrum of an authentic sample of arabinose and NaD_2PO_4 in D_2O at pD 7.0; F – ^{1}H NMR spectrum of an authentic sample of xylose and NaD_2PO_4 in D_2O at pD 7.0.

Supplementary Table 5 Summary of products.

Compound class	Products	Combined yield
C_1	MeOH<br>Formate	~1%
C_2	Glycolaldehyde **2**<br>Glyceronitrile **4**<br>Ethanolamine **9**<br>Ethylene glycol **6**<br>MeCN<br>EtOH<br>AcO^-	~8%
C_3	Glyceraldehyde **7**<br>Glyceraldehyde cyanohydrin **10**<br>Glycerol **11**<br>1,3-Dihydroxypropane	~11%
C_4	Tetroses **13**	~11%
C_5	Pentoses **14**	~29%

Summary of the products identified in each compound class (C_1-C_5) in Supplementary Fig. 15, spectrum A, and the total yield for all compounds identified in each class. Also see Supplementary Fig. 15, caption. Cyanohydrins were counted in the same class of compounds as the parent aldehyde *e.g.* glyceronitrile **4** was counted as a C_2 compound. This was to avoid misrepresentation of the overall yields for each stage of reduction *i.e.* an aldehyde belonging to the class of compounds C_n is converted to C_{n+1} when cyanide adds *in situ*, artificially depleting the yield of C_n and inflating the yield of C_{n+1} compounds. Glycolonitrile **1** was not included in the products, as it was not possible to know how much was synthesised *in situ* from HCN. Yields based on **1** and HCN.

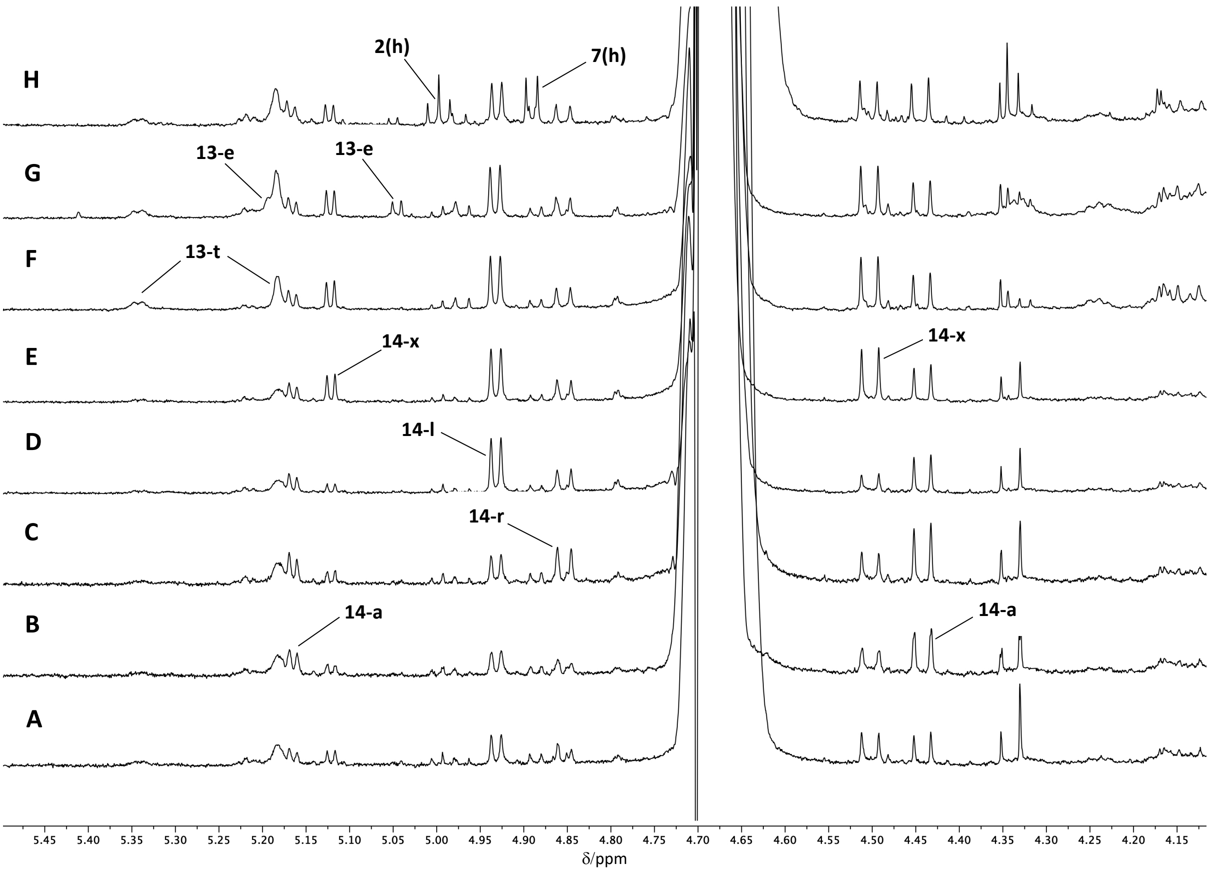

Supplementary Fig. 16 Synthesis of C_2, C_3, C_4 and C_5 sugars from HCN. A solution of KCN (0.110 mmol, 7 mg), $NaH_2PO_4.2H_2O$ (0.150 mmol, 23 mg) and Na_3PSO_3 (the purity and water content were predetermined and accounted for, 0.200 mmol) were dissolved in degassed 10% D_2O in H_2O (1 mL) and pH adjusted to 6.5. The volume was made up to 2 mL with degassed 10% D_2O in H_2O, then the solution irradiated. After 3 h, a ^1H NMR spectrum of the crude reaction mixture (spectrum A) was acquired; B – As spectrum A, spiked with commercial arabinose **14-a**; C – As spectrum B, spiked with commercial ribose **14-r**; D – As spectrum C, spiked with commercial lyxose **14-l**; E – As spectrum D, spiked with commercial xylose **14-x**; F – As spectrum E, spiked with commercial threose **13-t**; G – As spectrum F, spiked with commercial erythrose **13-e**; H – From the crude reaction mixture, a portion (900 μL) was removed and fumarate solution (pH 7, 50 mM, 100 μL) and H_2O (8 mL) were added. N_2 Was bubbled through the solution for 2 d to remove HCN, then spectrum H was obtained. Yields of the sugars were then determined. In spectrum H, there was ~ 1% glycolaldehyde **2**, ~ 2% glyceraldehyde **7**, ~ 5% tetroses **13**, ~ 19% pentoses **14** (**14-r**:**14-a**:**14-l**:**14-x**, 1:1.3:1.3:1.1). Due to signals from **14-r** and **14-d** (~ 4.78 ppm) being in very close proximity to the H_2O/HOD peak, and that the solvent suppression NMR experiment supressed these signals significantly, coupled with signal overlap between **14-r** and the tetroses **13** (5.17 ppm), the yields of **14-r** and **14-l** were calculated using the peak corresponding to the major cyclic form of each sugar and its relative abundance to the minor forms of each sugar, as observed and measured for the pure sugars in phosphate buffer (pD 7, Supplementary Fig. 15, spectra C and D). The calculated integration for the peak of **14-r** at 5.17 ppm could then be deducted from the yield

of **13**. As solvent suppression was used to acquire the ^{1}H NMR spectra, these yields are thought to be conservative due to some attenuation of the signals most proximal to the HOD/H_2O peak. Although the signals in the spectra look comparable in intensity, five cyanide molecules are required to form the pentoses **14**, thus the maximum yield is one fifth the initial concentration of HCN. The same considerations are taken for the other sugars. In spectrum H, there was ~ 3% C_1 (formate), ~ 9% C_2 (glycolaldehyde **2**, ethanolamine **9**, ethylene glycol **6**, thioacetamide, MeCN, lactonitrile **5**, acetaldehyde hydrate **3(h)**, acetate and EtOH (MeCN, lactonitrile **5**, acetaldehyde hydrate **3(h)** and EtOH calculated from spectrum A), ~ 7% C_3 (glyceraldehyde **7**, glycerol **11** and 1,3-dihydroxypropane), ~ 6% C_4 (tetroses **13**) and ~ 19% C_5 compounds (pentoses **14**).

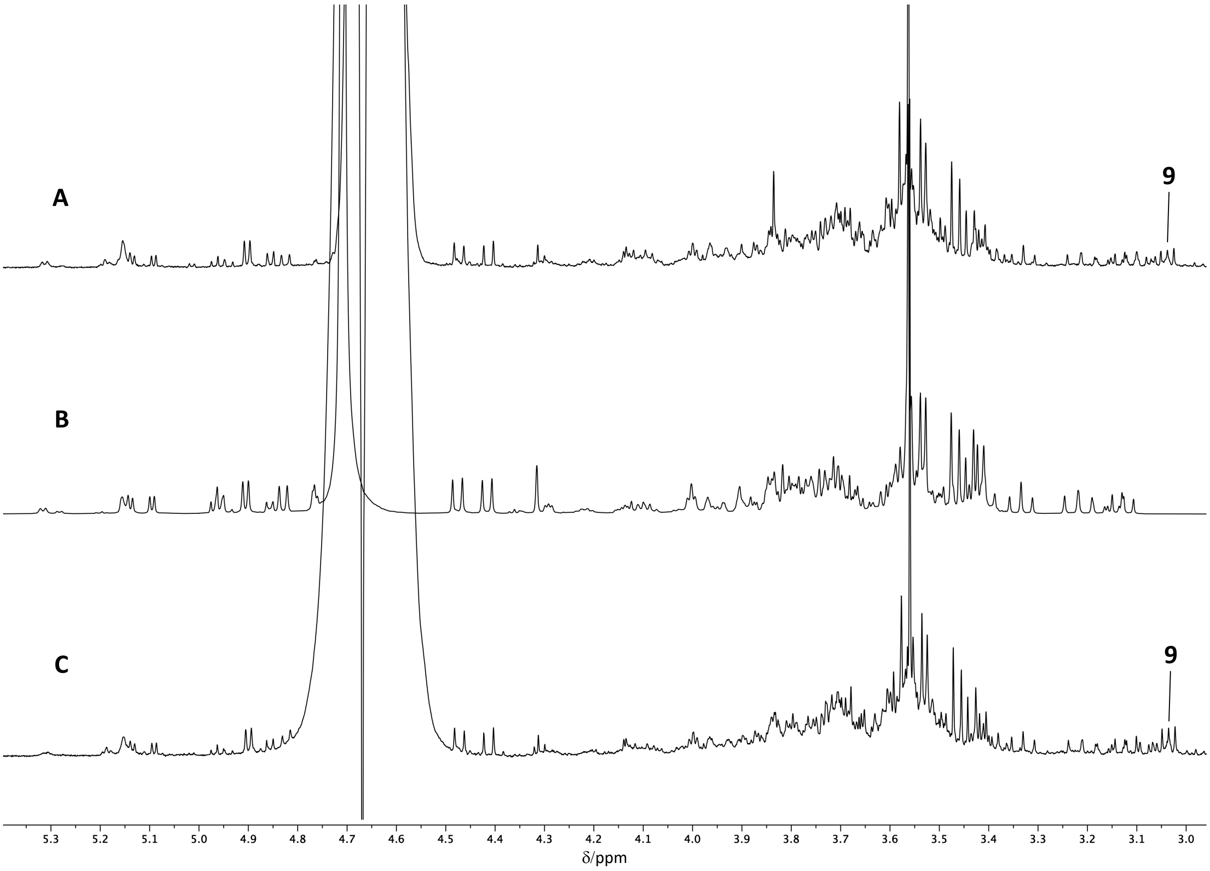

Supplementary Fig. 17 A – A solution of KCN (0.090 mmol, 6 mg), $NaH_2PO_4.2H_2O$ (0.150 mmol, 23 mg) and Na_3PSO_3 (the purity and water content were predetermined and accounted for, 0.200 mmol) were dissolved in degassed 10% D_2O in H_2O (1 mL) and pH adjusted to 6.5. Glycolonitrile **1** (0.015 mmol, 3.0 μL) was added, the volume made up to 2 mL with degassed 10% D_2O in H_2O then the solution irradiated. After 2 h 40 min, a portion (1 mL) was removed, diluted with H_2O (7 mL) then N_2 bubbled through for 2 d, after which the volume was ~ 1 mL. An aliquot was removed, D_2O added and a ^1H NMR spectrum (spectrum A) acquired; B – A ^1H NMR spectrum of a roughly equimolar mixture of glycolaldehyde **2**, glyceraldehyde **7**, threose **13-t** (erythrose **13-e** was not included due to the high concentration of impurities present in commercial samples, 30 - 40%), the pentoses **14**, ethylene glycol **6** and glycerol **11** in NaD_2PO_4 buffer in pure D_2O at pD 7; C – A solution of KCN (0.110 mmol, 7 mg), $NaH_2PO_4.2H_2O$ (0.150 mmol, 23 mg) and Na_3PSO_3 (the purity and water content were predetermined and accounted for, 0.200 mmol) were dissolved in degassed 10% D_2O in H_2O (1 mL) and pH adjusted to 6.5. The volume was made up to 2 mL with degassed 10% D_2O in H_2O, then the solution irradiated. After 3 h, a portion (1 mL) was removed, diluted with H_2O (7 mL) then N_2 bubbled through for 2 d, after which the volume was ~ 1 mL. An aliquot was removed, D_2O added and a ^1H NMR spectrum (spectrum C). Disregarding relative intensities, the majority of signals are accounted for by the mixture represented by spectrum B. Ethanolamine **9** was identified subsequently, the corresponding triplet is not clearly visible but is found at 3.63 ppm.

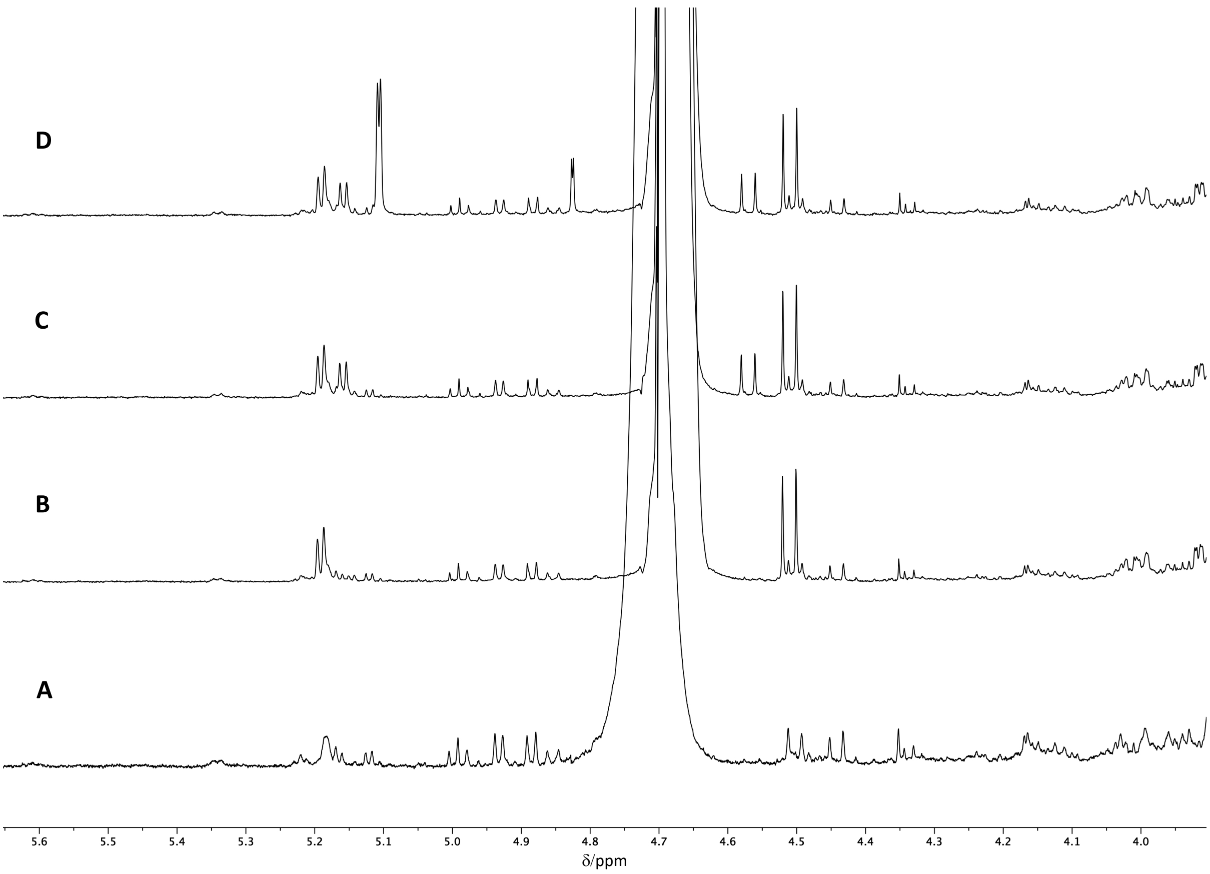

Supplementary Fig. 18 Absence of C_6 sugars from the reaction mixture when C_5 sugars are formed. A – A solution of KCN (0.090 mmol, 6 mg), $NaH_2PO_4.2H_2O$ (0.150 mmol, 23 mg) and Na_3PSO_3 (the purity and water content were predetermined and accounted for, 0.200 mmol) were dissolved in degassed 10% D_2O in H_2O (1 mL) and pH adjusted to 6.5. Glycolonitrile **1** (0.015 mmol, 3.0 μL) was added, the volume made up to 2 mL with degassed 10% D_2O in H_2O then the solution irradiated. After 2.5 h, a ^{1}H NMR spectrum of the crude reaction mixture (spectrum A) was acquired; B – As spectrum A, after spiking with a commercial sample of galactose; C – As spectrum B, after spiking with a commercial sample of glucose; D – As spectrum C, after spiking with a commercial sample of mannose. As there was effectively no selectivity for the diastereoselective formation of one tetrose or pentose over another, it was assumed no selectivity would be observed in the potential formation of hexoses, hence glucose, galactose and mannose were used as representative examples.

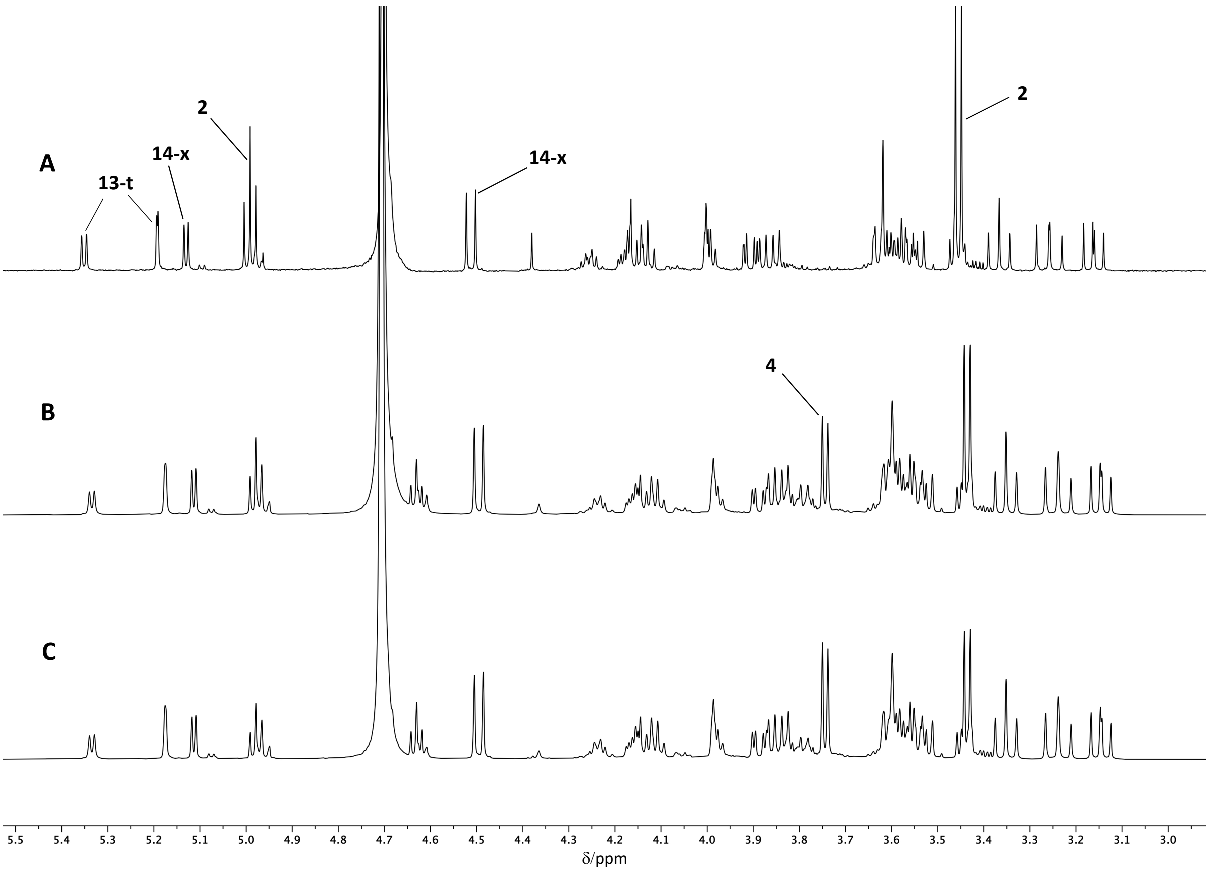

Supplementary Fig. 19 Competition experiment of a C_2, C_4 and C_5 sugar for HCN. To facilitate observation and quantification of the system, glyceraldehyde **7** was omitted from the mixture and only one tetrose and one pentose were added (threose **13-t** and xylose **14-x** were chosen as they gave resolved signals in the ^1H NMR spectrum of the mixture). A – NaD_2PO_4 (*ca.* 0.200 mmol, 26 mg) Was dissolved in D_2O (1.4 mL) and the pD adjusted to 6.6 with NaOD. Glycolaldehyde **2** (0.100 mol, 6 mg), **13-t** (≥ 60%, 0.100 mol, 20 mg), **14-x** (0.100 mmol, 15 mg) and sodium succinate (added as an internal standard, 0.050 mmol, 8 mg, not shown) were added and a ^1H NMR spectrum acquired (spectrum A); B – As spectrum A, 15 min after the addition of KCN (0.100 mmol, 6 mg), re-adjustment of the pD to 6.9 and the volume made up to 2 mL with D_2O; C – As spectrum B after 16 h. Using succinate as a reference, in spectrum A the integration of **2**:**13-t**:**14-x** was 1.42:1.65:1.59, in spectrum B it was 0.87:1.22:1.60 and in spectrum C it was 0.71:1.25:1.61.

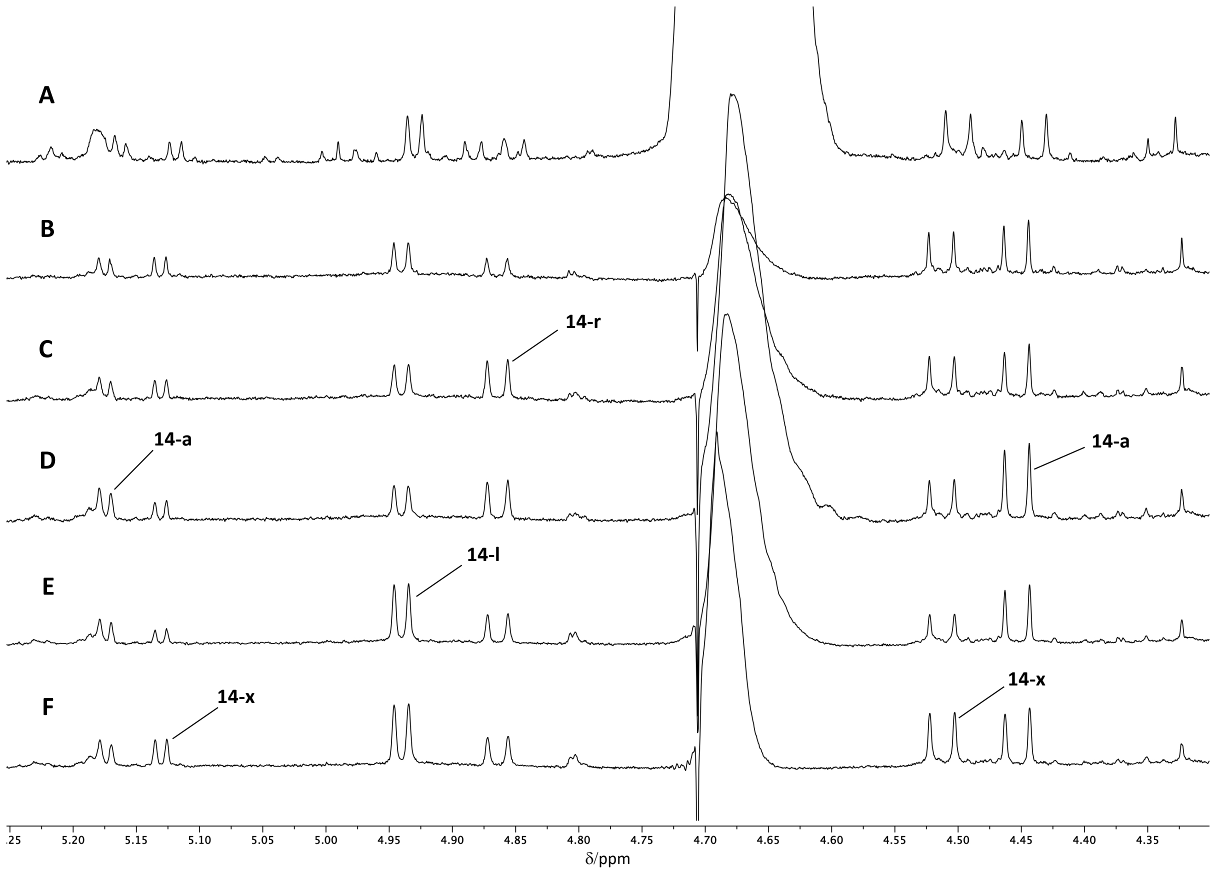

Supplementary Fig. 20 Pentoses **14** remain after evaporation to dryness. A – A solution of KCN (0.090 mmol, 6 mg), $NaH_2PO_4.2H_2O$ (0.150 mmol, 23 mg) and Na_3PSO_3 (the purity and water content were predetermined and accounted for, 0.200 mmol) were dissolved in degassed 10% D_2O in H_2O (1 mL) and pH adjusted to 6.5. Glycolonitrile **1** (0.015 mmol, 3.0 μL) was added, the volume made up to 2 mL with degassed 10% D_2O in H_2O then the solution irradiated. After 2.75 h an aliquot (450 μL) of the reaction was removed and succinate (pH 7, 50 mM, 50 μL) was added, then a ^1H NMR spectrum was acquired (spectrum A); From the remaining reaction, a portion (900 μL) was removed, succinate (pH 7, 50 mM, 100 μL) added and the sample was diluted with H_2O (~ 7 mL). N_2 Was bubbled through the solution until a dry residue remained (~ 3 d). The residue was dissolved in D_2O and spectrum B was acquired; C – As spectrum B, spiked with a commercial sample of ribose **14-r**; D – As spectrum C, spiked with a commercial sample of arabinose **14-a**; E – As spectrum D, spiked with a commercial sample of lyxose **14-l**; F – As spectrum E, spiked with a commercial sample of xylose **14-x**.

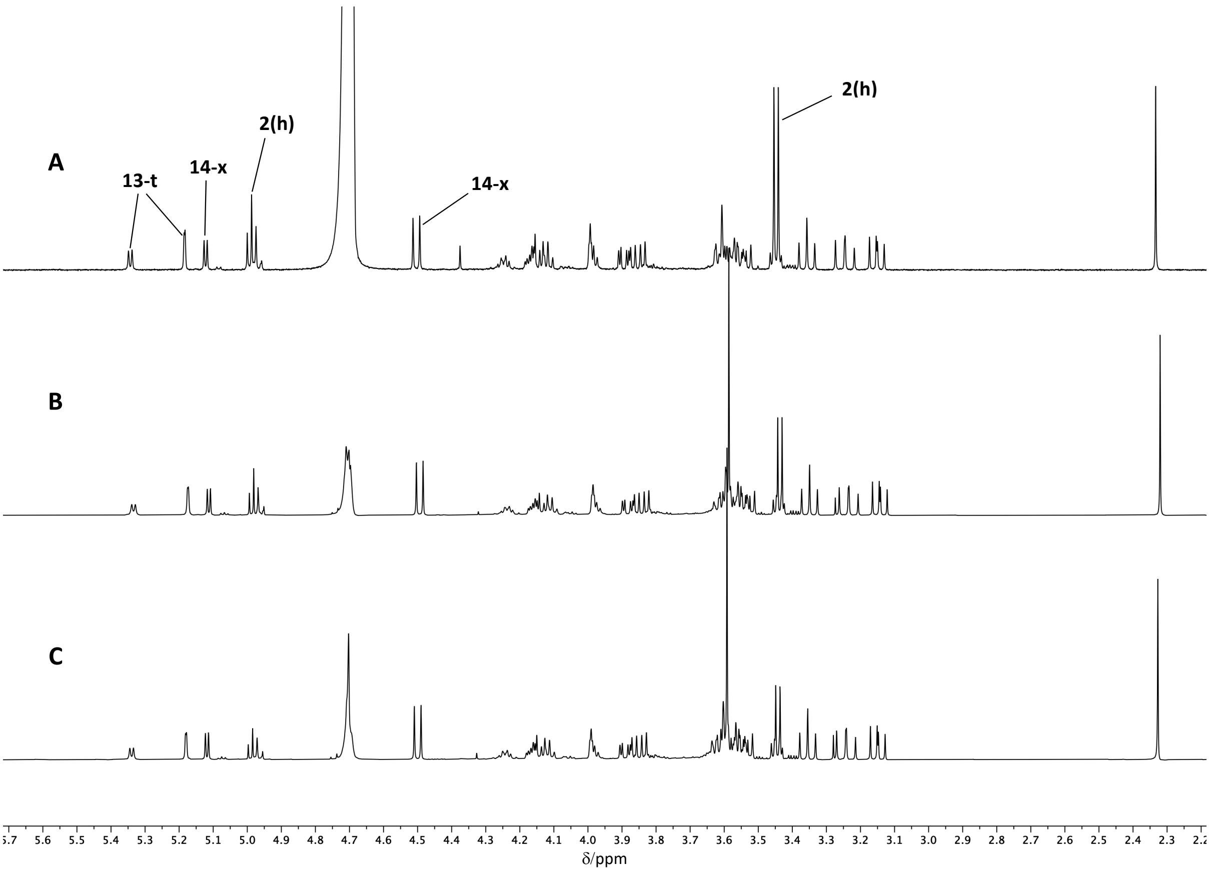

Supplementary Fig. 21 Competition experiment showing the stability of pentoses **14** to photochemical reduction. The system was simplified to include only glycolaldehyde **2**, threose **13-t** and xylose **14-x**, as they gave resolved signals in the ^{1}H NMR spectrum of the mixture and eased quantification. Glycolaldehyde **2** (18 mg, 0.300 mmol) and xylose **14-x** 45 mg, 0.300 mmol) were dissolved in H_2O (0.5 mL) and threose **13-t** syrup (≥ 60%) was added until roughly equimolar (as judged by removing several μL, diluting with D_2O and obtaining a ^{1}H NMR spectrum). Sodium succinate (10 mg, 0.062 mmol) was added as a standard and the volume adjusted to 2 mL with H_2O. From this stock solution, a portion was removed (0.5 mL), $NaH_2PO_4.2H_2O$ (31 mg, 0.200 mmol) and $Na_3PSO_3.xH_2O$ (purity and water content predetermined and accounted for, 0.075 mmol) were added, then the pH was adjusted to ~ 7 and the volume increased to 2 mL. The solution was irradiated and inspected at timepoints by ^{1}H NMR spectroscopy. A – ^{1}H NMR Spectrum of the starting mixture, singlet at 2.3 ppm due to the added succinate standard; B – As spectrum A, after 0.5 h of irradiation; C – As spectrum A, after 1 h of irradiation. Note the large singlet in spectra B and C at 3.6 ppm, which is due to ethylene glycol **6**. In spectrum A, the relative integration of **2**:**13-t**:**14-x** (referenced to succinate) was 3.4:4.1:4.3. In spectrum C, the relative integration of **2**:**13-t**:**14-x** (referenced to succinate) was 1.6:3.0:4.4. Therefore, 47% of **2** survives, 73% of **13-t** survives and all of **14-t** survives.

Supplementary Table 6 Comparison of stability of sugars to photoreduction or photodecomposition

Entry	Spectrum	Glycolaldehyde **1**	Threose **13-t**	Xylose **14-x**	PSO_3^{3-}	PO_4^{3-}	Irradiation time	Percentage decomposition		
								Glycolaldehyde **2**	Threose **13-t**	Xylose **14-x**
1	Supp. Fig. 21, Spectrum A	~38 mM	~32 mM	~38 mM	~38 mM	100 mM	1 h	~53%[a]	~27%[a]	trace[a]
2	Supp. Fig. 22, Spectrum A	~38 mM	~32 mM	~38 mM	–	100 mM	2 h	~24%	~10%	trace

[a] - percentage decomposition calculated from Supplementary Fig. 21, Spectrum C
trace - 1% or less

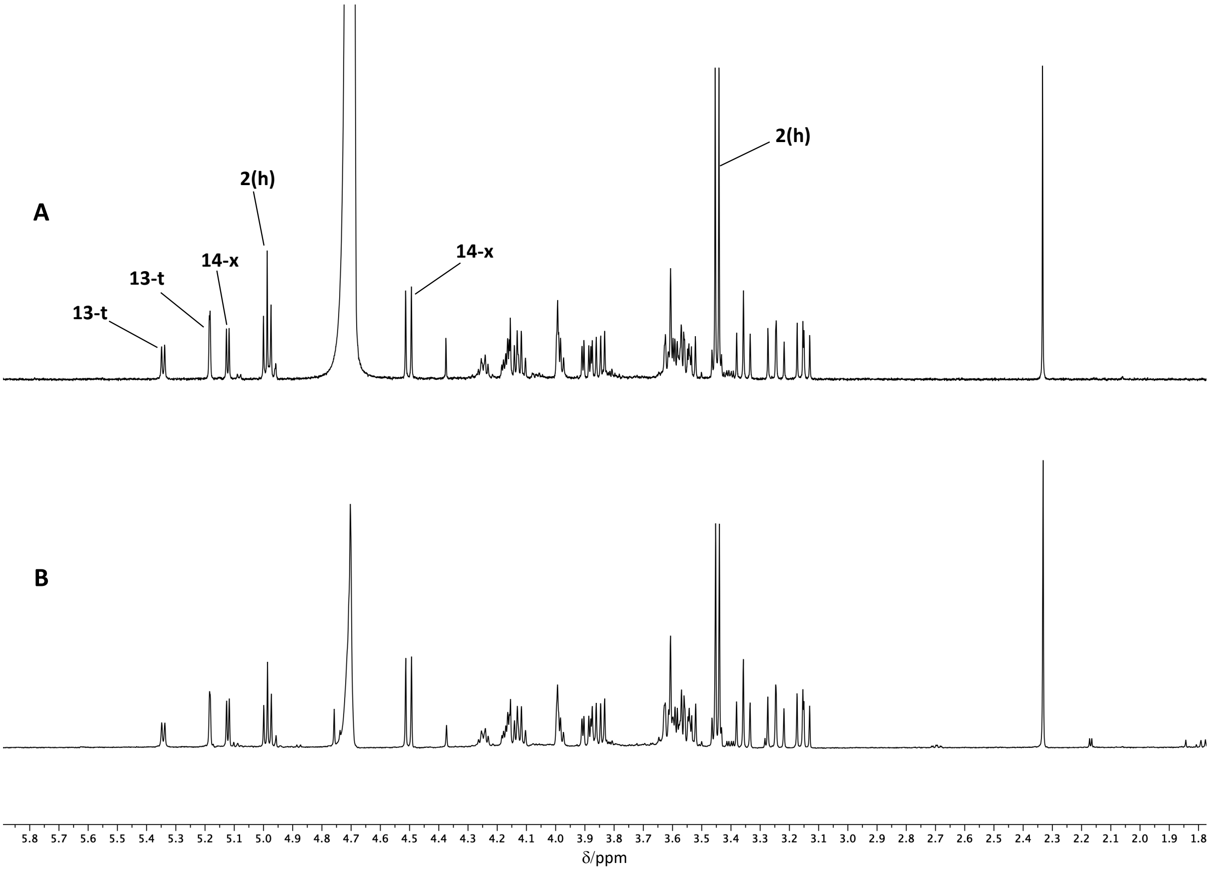

Supplementary Fig. 22 Differing rates of photochemical destruction of open-chain, predominantly cyclic and almost exclusively cyclic sugars. To facilitate observation and quantification of the system, glyceraldehyde **7** was omitted from the mixture and only one tetrose and one pentose were added (threose **13-t** and xylose **14-x** were chosen as they gave resolved signals in the ^{1}H NMR spectrum of the mixture). Glycolaldehyde **2** (18 mg, 0.300 mmol) and xylose **14-x** 45 mg, 0.300 mmol) were dissolved in H_2O (0.5 mL) and threose **13-t** syrup (≥ 60%) was added until roughly equimolar (as judged by removing several μL, diluting with D_2O and obtaining a ^{1}H NMR spectrum). Sodium succinate (~ 10 mg, ~ 0.062 mmol) was added as a standard, and the volume adjusted to 2 mL with H_2O. From this stock solution, a portion was removed (0.5 mL), $NaH_2PO_4.2H_2O$ (31 mg, 0.200 mmol) added, then the pH was adjusted to ~ 7 and the volume increased to 2 mL. The solution was then irradiated for 2 h. A – ^{1}H NMR Spectrum of the starting mixture, singlet at 2.3 ppm due to the added succinate standard; B – As spectrum A, after 2 h of irradiation. In spectrum A, the relative integration of **2**:**13-t**:**14-x** (referenced to succinate) was 3.4:4.1:4.3. In spectrum B, the relative integration of **2**:**13-t**:**14-x** (referenced to succinate) was 2.6:3.7:4.4. Therefore, 76% of **2** survives, 90% of **13-t** survives and all of **14-t** survives.

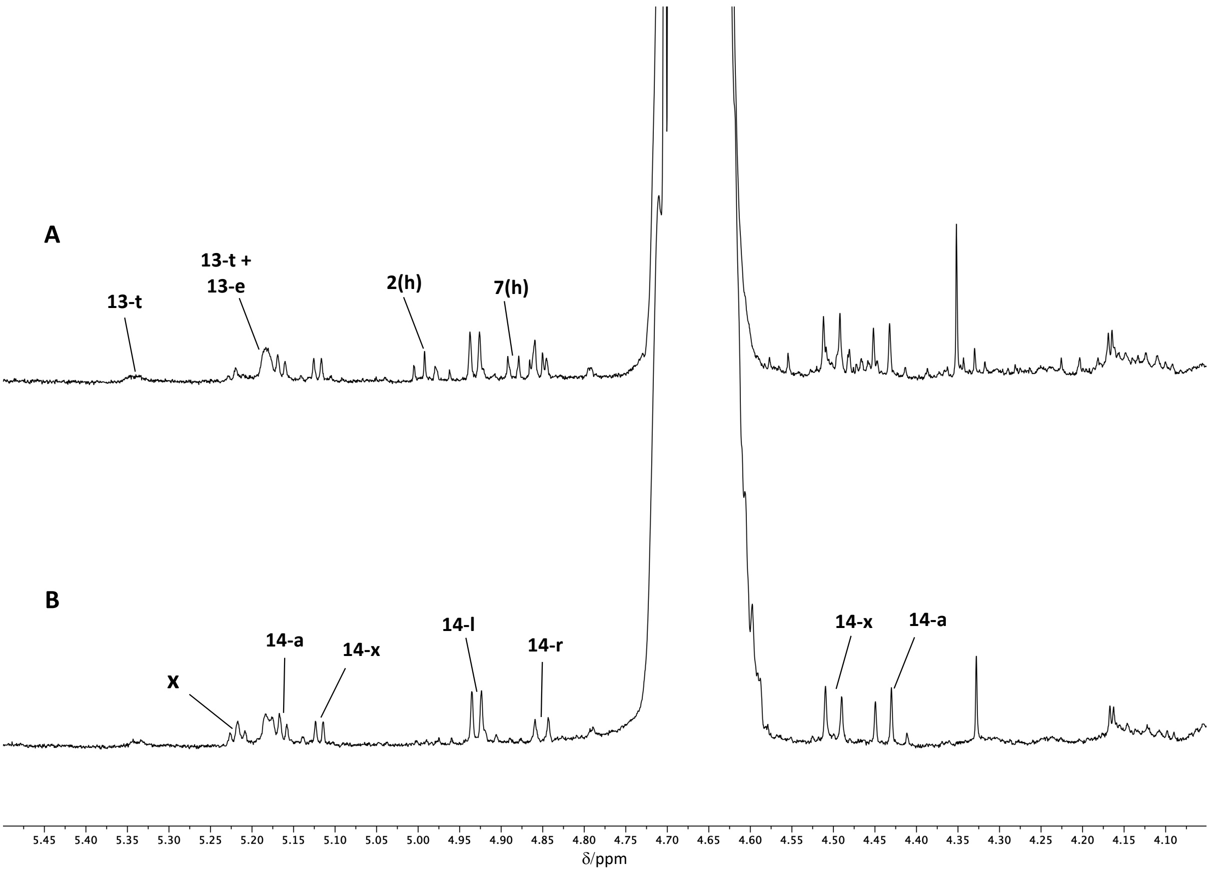

Supplementary Fig. 23 Stability of pentoses **14** under prolonged conditions of their synthesis. A solution of KCN (0.090 mmol, 6 mg), $NaH_2PO_4.2H_2O$ (0.150 mmol, 23 mg) and Na_3PSO_3 (the purity and water content were predetermined and accounted for, 0.200 mmol) were dissolved in degassed 10% D_2O in H_2O (1 mL) and pH adjusted to 6.5. Glycolonitrile **1** (0.015 mmol, 3.0 μL) was added, the volume made up to 2 mL with degassed 10% D_2O in H_2O then the solution irradiated. A – ^{1}H NMR Spectrum of the crude reaction mixture following the preceding procedure after 2.5 h, succinate added as a reference (not shown); B – As spectrum A, after 5 h irradiation. As can be seen, after 5 h irradiation, the reaction mixture is almost devoid of glycolaldehyde **2** and glyceraldehyde **7**. Although the tetroses **13** have diminished somewhat in intensity after 5 h reaction compared to 2.5 h, the abundance of pentoses **14** have actually increased - in spectrum A there is ~ 14% of **14** and in spectrum B there is ~ 21%. **X** is an unidentified compound(s).

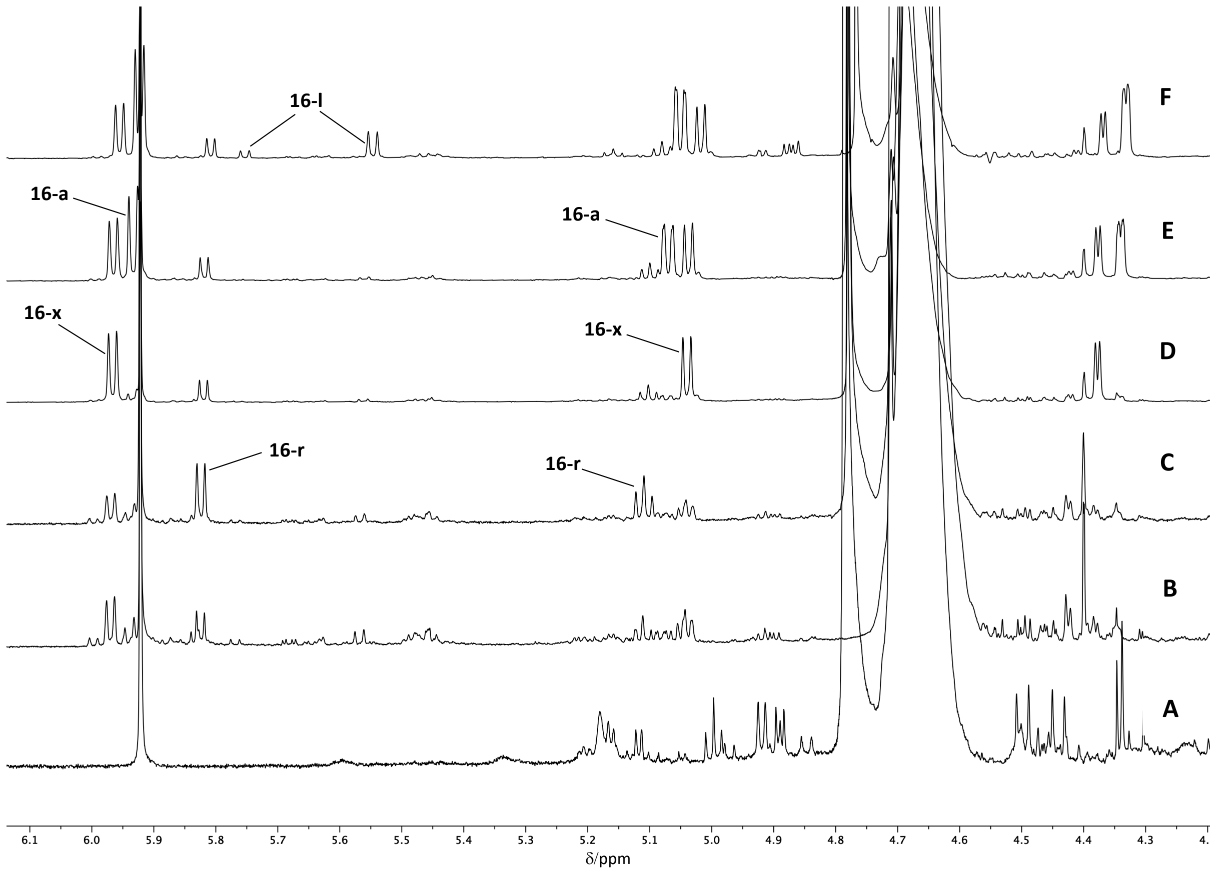

Supplementary Fig. 24 Synthesis of pentose aminooxazolines **16** from glycolonitrile **1**, HCN and NH_2CN. A solution of KCN (0.270 mmol, 18 mg), $NaH_2PO_4.2H_2O$ (0.450 mmol, 70 mg) and Na_3PSO_3 (the purity and water content were predetermined and accounted for, 0.600 mmol) were dissolved in degassed 10% D_2O in H_2O (2 mL) and pH adjusted to 6.5 with degassed 6 M HCl. Glycolonitrile **1** (0.045mmol, 4.5 μL) was added, the volume made up to 6 mL with degassed 10% D_2O in H_2O and the solution was divided into 3 cuvettes before being irradiated for 2.75 h. The contents of the cuvettes were combined and N_2 was bubbled through the solution for 18 h, at which point ~ 0.9 mL remained. A portion of this crude reaction mixture (450 μL) was then removed and succinate (pH 7, 50 μL, 50 mM) added before a ^{1}H NMR spectrum was acquired (spectrum A). To the NMR tube was charged NH_2CN (15 mg, 0.357 mmol) and, after mixing, was heated to 60 °C for 2 h. The tube was removed from the oil bath and spectrum B acquired; C – As spectrum B, spiked with an authentic sample of *ribo*-aminooxazoline **16-r**; D – As spectrum C, spiked with an authentic sample of *xylo*-aminooxazoline **16-x**; E – As spectrum D, spiked with an authentic sample of *arabino*--aminooxazoline **16-a**; F – As spectrum E, spiked with an authentic sample of *lyxo*-aminooxazoline **16-l**. *Ribo*-Aminooxazoline was formed in ~ 1% yield based on the initial concentration of **1** and HCN.

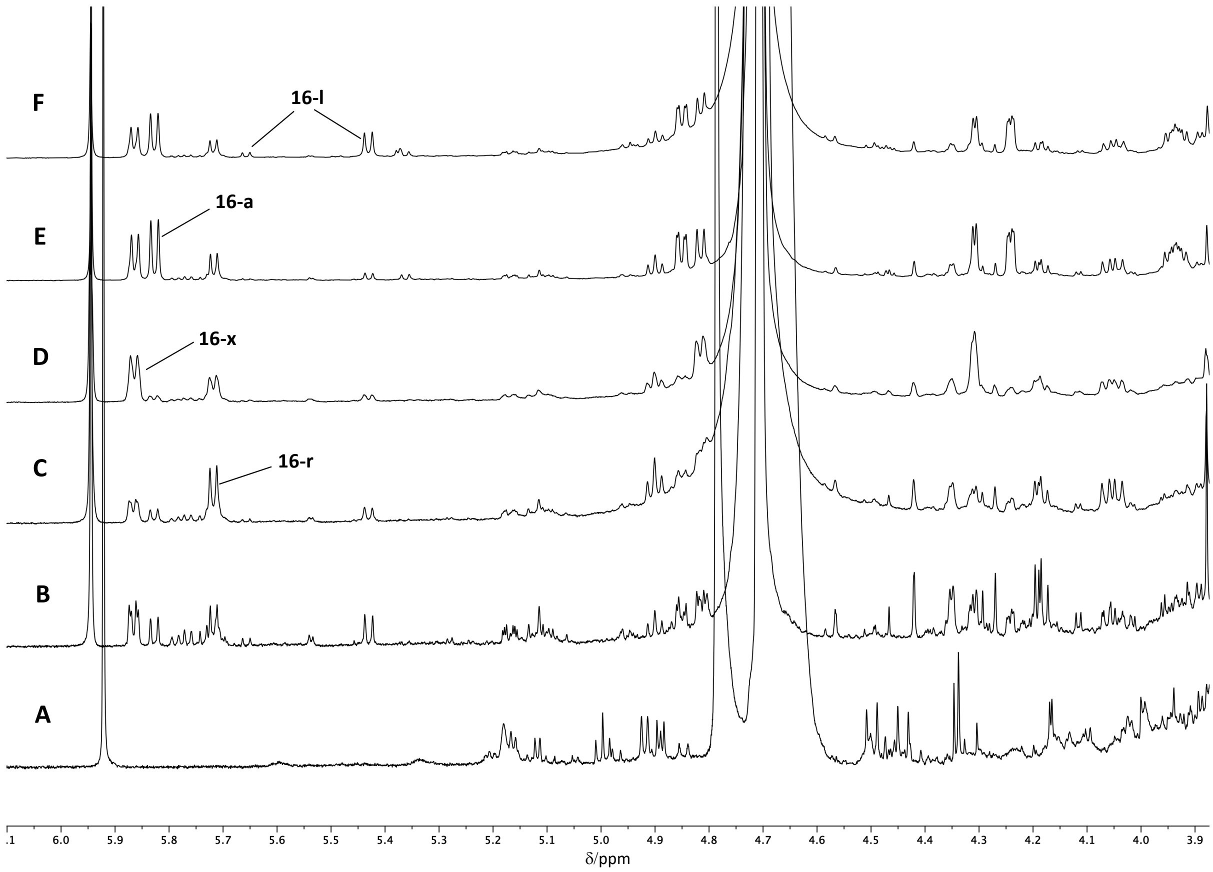

Supplementary Fig. 25 Synthesis of pentose aminooxazolines **16** from glycolonitrile **1**, HCN and $CaCN_2$. A – As spectrum A, Supplementary Fig. 24; B – Following the procedure outlined in Supplementary Fig. 24, to the remaining concentrated, crude reaction mixture (~ 450 μL), $CaCN_2$ (~ 85%, 28 mg, 0.300 mmol) was added, then the suspension was sealed and heated to 60 °C for 2 h with stirring. The suspension was briefly centrifuged, then the supernatant was removed and examined by ^{1}H NMR spectroscopy – spectrum B; C – As spectrum B, spiked with an authentic sample of *ribo*-aminooxazoline **16-r**; D – As spectrum C, spiked with an authentic sample of *xylo*-aminooxazoline **16-x**; E – As spectrum D, spiked with an authentic sample of *arabino*-aminooxazoline **16-a**; F – As spectrum E, spiked with an authentic sample of *lyxo*-aminooxazoline **16-l**.

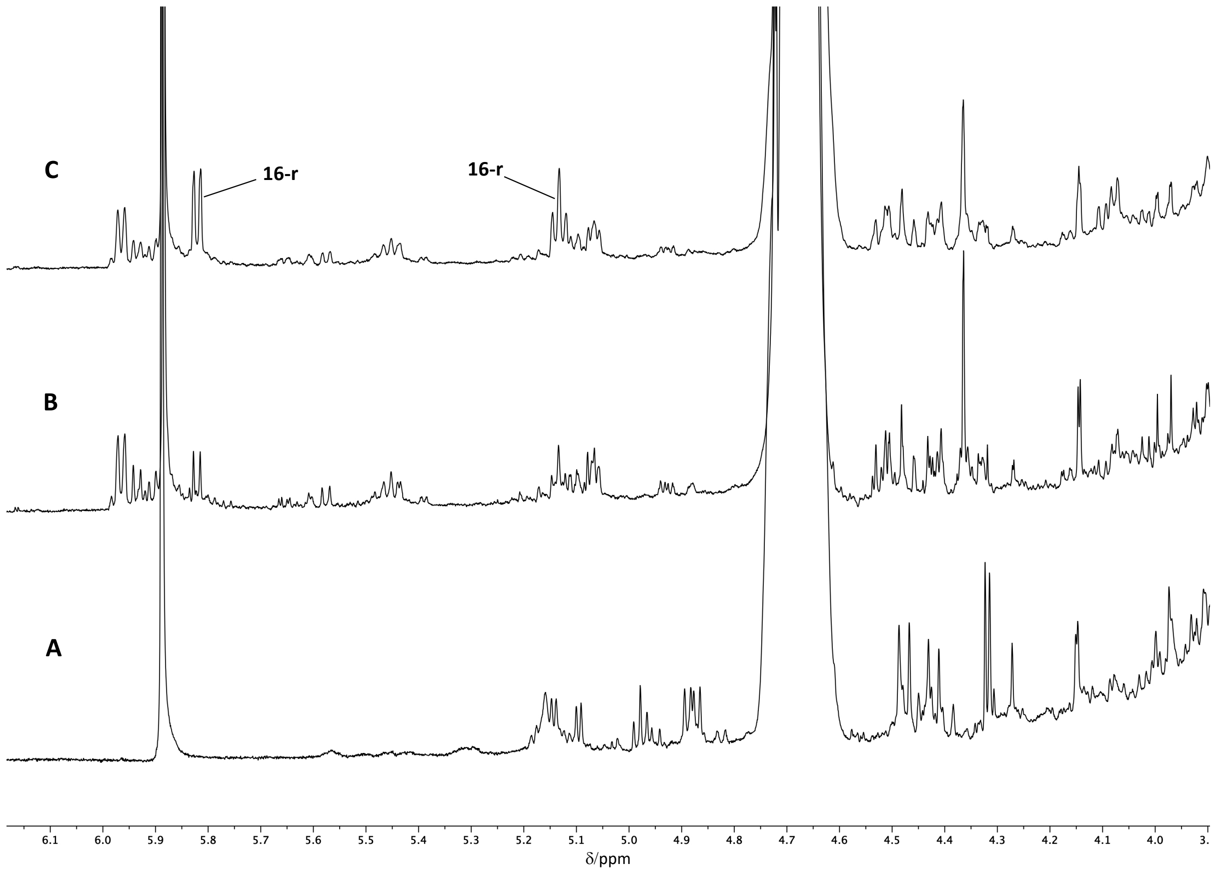

Supplementary Fig. 26 Synthesis of pentose aminooxazolines **16** from HCN and NH_2CN. A solution of KCN (0.330 mmol, 22 mg), $NaH_2PO_4.2H_2O$ (0.450 mmol, 70 mg) and Na_3PSO_3 (the purity and water content were predetermined and accounted for, 0.600 mmol) were dissolved in degassed 10% D_2O in H_2O (2 mL) and pH adjusted to 6.5 with degassed 6 M HCl. The volume was made up to 6 mL with degassed 10% D_2O in H_2O and the solution was divided into 3 cuvettes before being irradiated for 3 h. The contents of the cuvettes were combined and N_2 was bubbled through the solution for 18 h, at which point ~ 0.6 mL remained. A portion of this crude reaction mixture (450 μL) was then removed and succinate (pH 7, 50 μL, 50 mM) added before a ^{1}H NMR spectrum was acquired (spectrum A). To the NMR tube was charged NH_2CN (15 mg, 0.357 mmol) and, after mixing, was heated to 60 °C for 2.5 h. The tube was removed from the oil bath and spectrum B acquired; C – As spectrum B, spiked with an authentic sample of *ribo*-aminooxazoline **16-r**. Yield of *ribo*-aminooxazoline **16-r** ~ 1% starting from HCN.

Procedure 2

Dihydroxyacetone **12** or glyceraldehyde **7** (0.030 mmol, 2.7 mg), $Na_3PSO_3.xH_2O$ (purity and water content predetermined and accounted for, 2 equiv.) and $NaH_2PO_4.2H_2O$ (if used, 0.060 mmol, 9 mg) were dissolved in an Eppendorf tube with degassed 10% D_2O in H_2O (1 mL), and the pH was adjusted to 6.5 with degassed HCl. The volume was made up to 2 mL with degassed 10% D_2O in H_2O, and the solution was transferred to a quartz cuvette and sealed. The reaction was irradiated for the desired amount of time, after which it was analysed by ^1H NMR spectroscopy.

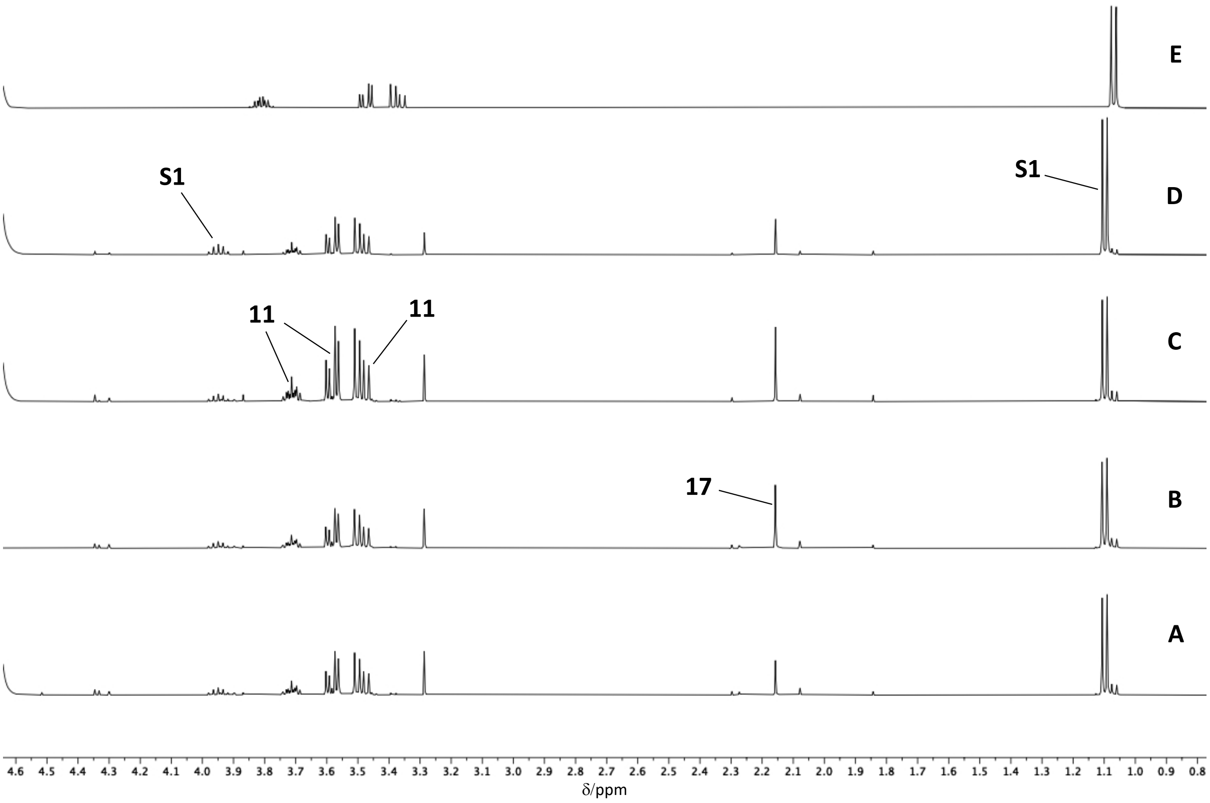

Supplementary Fig. 27 Photochemical reduction of dihydroxyacetone **12** to glycerol **11** in the absence of PO_4^{3-}. A – ^1H NMR Spectrum of the reaction according to Procedure 2 after 0.5 h using **12** as the starting material and no added phosphate; B – As spectrum A, spiked with a commercial sample of acetone **17** (2.15 ppm); C – As spectrum B, spiked with a commercial sample of **11**; D – As spectrum C, spiked with a commercial sample of isopropanol **S1**; E – ^1H NMR Spectrum of an authentic sample of propan-1,2-diol. The fact that only 5% of propan-1,2-diol was formed as compared to 22% of isopropanol shows that α-dehydroxylation predominates over 1,2-reduction as the preferred, one electron reduction pathway for hydroxyacetone. Yields estimated by addition of disodium fumarate (50 mM solution, 50 μL into 450 μL of crude reaction) as a standard and relative integration of ^1H NMR signals.

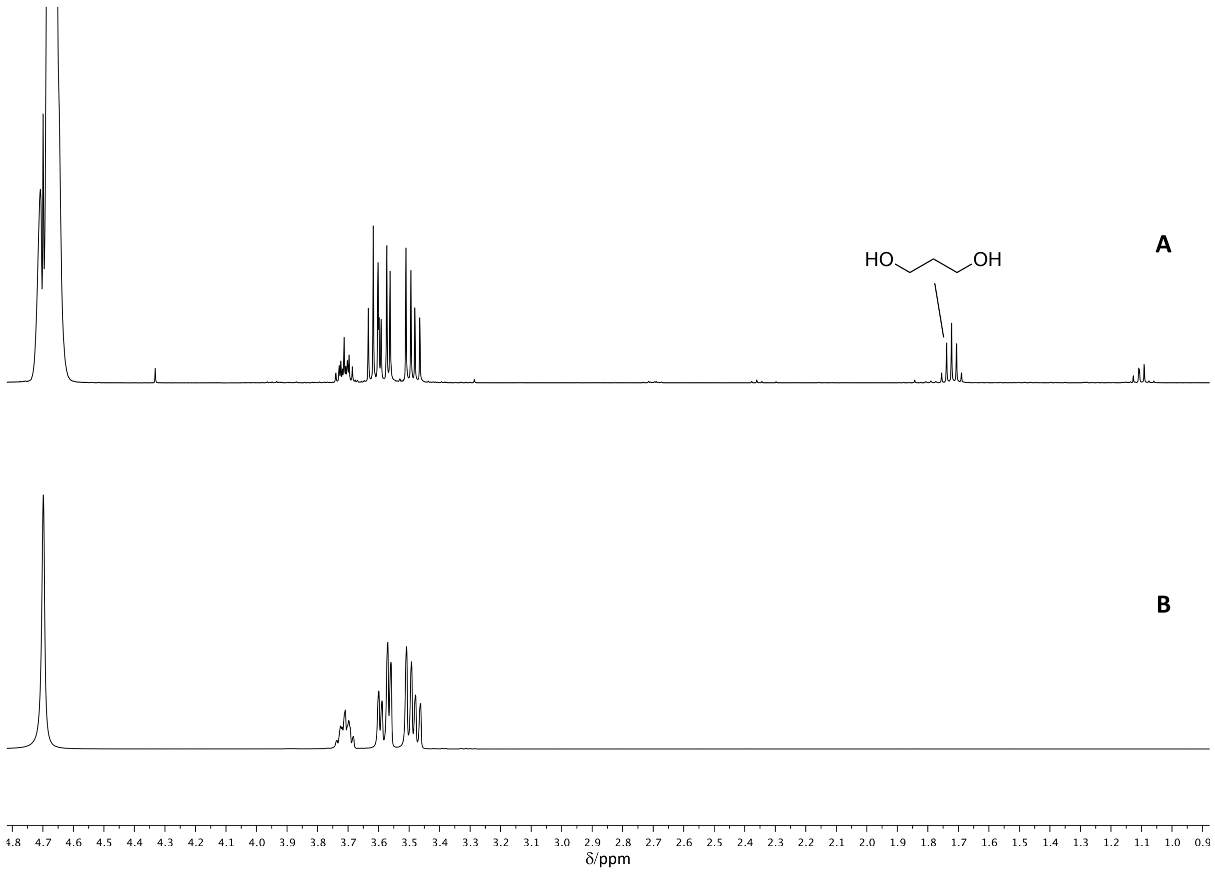

Supplementary Fig. 28 Photochemical reduction of glyceraldehyde **7** to glycerol **11** in the absence of PO_4^{3-}. A – ^{1}H NMR Spectrum of the reaction according to Procedure 2 after 45 min using **7** as the starting material and no added phosphate; B – ^{1}H NMR Spectrum of a commercial sample of **11**. Yields estimated by addition of disodium fumarate (50 mM solution, 50 μL into 450 μL of crude reaction) as a standard and relative integration of ^{1}H NMR signals.

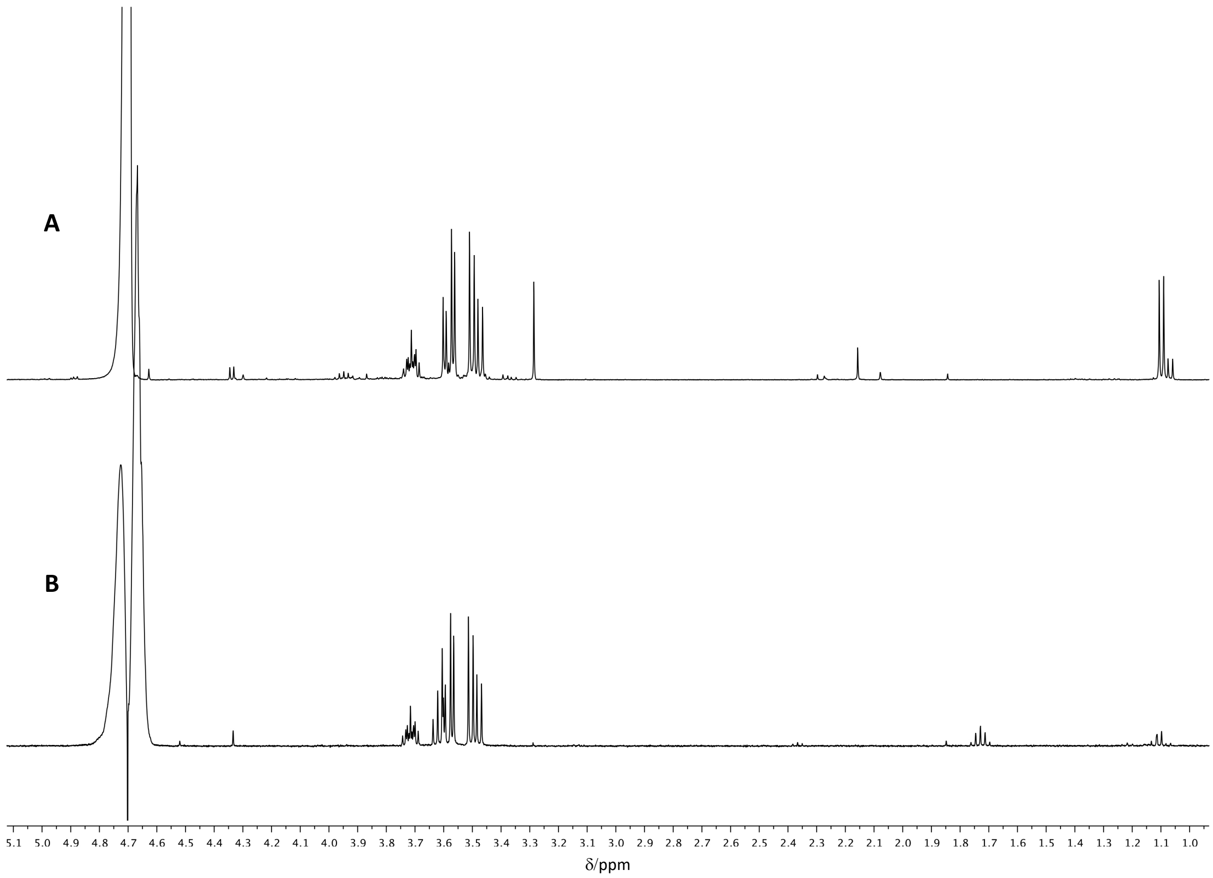

Supplementary Fig. 29 Photochemical reduction of dihydroxyacetone **12** and glyceraldehyde **7** to glycerol **11** in the presence of PO_4^{3-}. A – ^{1}H NMR Spectrum of the reaction according to Procedure 2 after 0.5 h using **12** as the starting material with phosphate buffer included; B – ^{1}H NMR Spectrum of the reaction according to Procedure 2 after 45 min using **7** as the starting material with phosphate buffer included. Yields estimated by addition of disodium fumarate (50 mM solution, 50 μL into 450 μL of crude reaction) as a standard and relative integration of ^{1}H NMR signals.

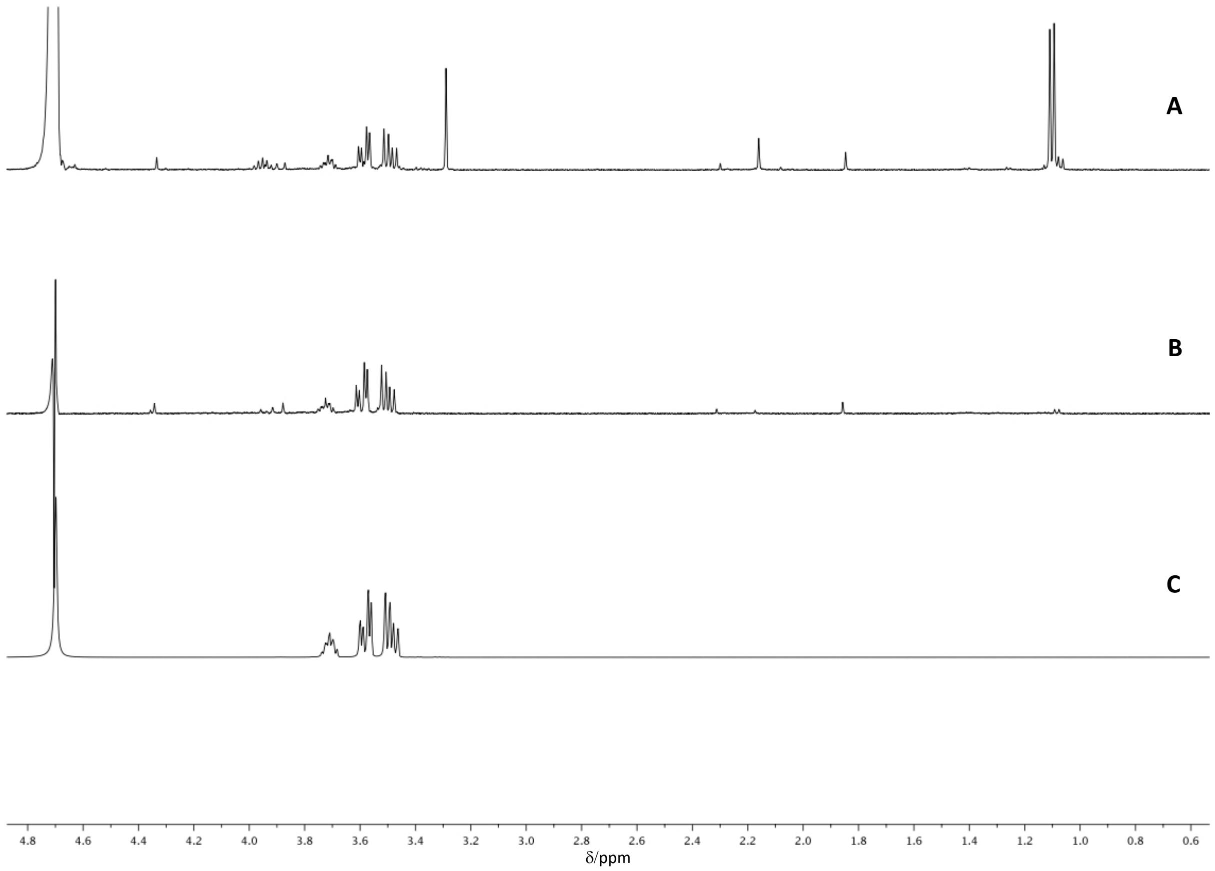

Supplementary Fig. 30 Synthesis and concentration of glycerol **11**. A – ^{1}H NMR Spectrum after 1 h of reaction carried out according to Procedure 2 using dihydroxyacetone **12** as the starting material (1.2 equiv. of Na_3PSO_3 was used, no phosphate included); B – As spectrum A after being concentrated to dryness (a rotary evaporator was used with the water bath set at 45 °C) and then re-dissolved in D_2O; C – ^{1}H NMR Spectrum of a commercial sample of **11**.

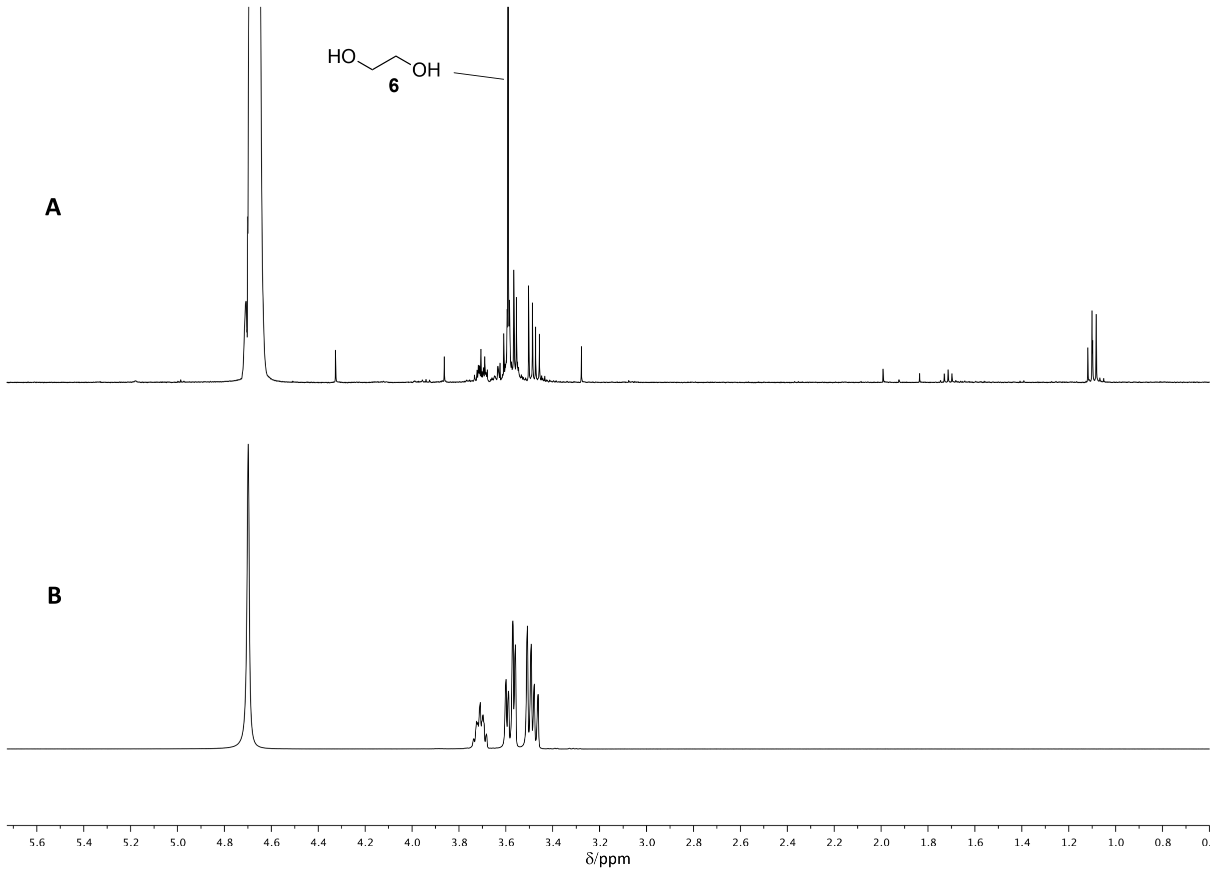

Supplementary Fig. 31 Synthesis of glycerol **11** from glycolonitrile **1** and HCN. A – A solution of KCN (0.020 mmol, 1.3 mg), $NaH_2PO_4.2H_2O$ (0.150 mmol, 23 mg) and $Na_3PSO_3.xH_2O$ (purity and water content predetermined and accounted for, 0.150 mmol) in degassed 10% D_2O in H_2O (1 mL) was adjusted to pH 6.5. The volume was made up to 2 mL using degassed 10% D_2O in H_2O and **1** (0.020 mmol, 2.0 μL) was added. The reaction was then irradiated for 2.5 h and a ^1H NMR spectrum was acquired of the crude reaction mixture (spectrum A); B – ^1H NMR Spectrum of a commercial sample of glycerol. Yields estimated by addition of disodium fumarate (50 mM solution, 50 μL into 450 μL of crude reaction) as a standard and relative integration of ^1H NMR signals.

Procedure 3

Dibasic thiophosphate was prepared according to General procedure 1 (0.100 mmol). Glycerol **11** (4.6 mg, 0.050 mmol) and acrylonitrile (if used, 0.200 mmol, 11 mg, 13.1 μL) were dissolved in degassed formamide (1 mL) then added to $HPSO_3^{2-}$ in an Eppendorf tube, sealed and either left at room temperature or heated to 70 °C. Samples were removed at the desired time points, diluted with D_2O and examined by ^{31}P NMR spectroscopy.

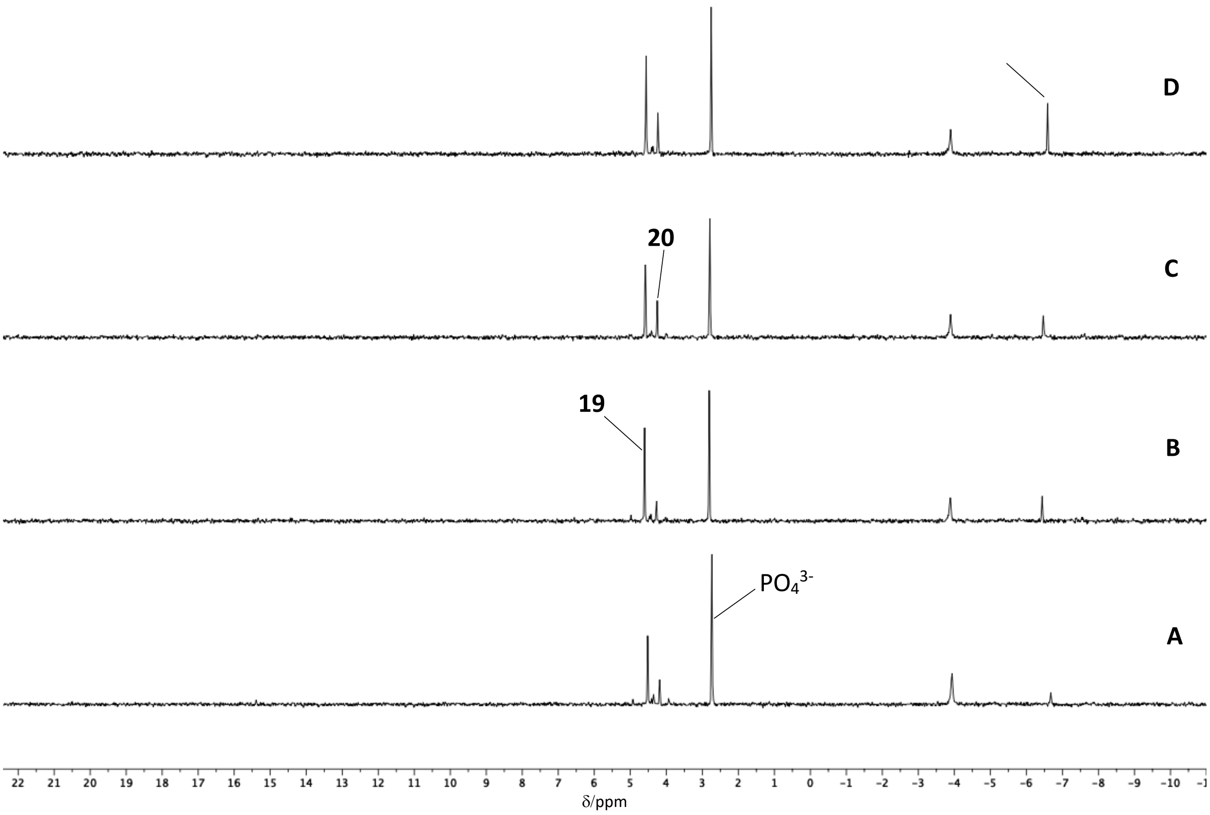

Supplementary Fig. 32 Phosphorylation of glycerol **11** using PSO_3^{3-} and acrylonitrile. A – ^{31}P NMR Spectrum after 3 h of reaction carried out according to Procedure 3 at 70 °C (glycerol-1-phosphate **19** ~ 30% yield and glycerol-2-phosphate **20** in ~ 11% yield); B – As spectrum A, after being spiked with a commercial sample of glycerol-1-phosphate **19**; C – As spectrum B, after being spiked with a commercial sample of glycerol-2-phosphate **20**; D – As spectrum C, after being spiked with commercial pyrophosphate. The other minor signals from 3.5 ppm to 5.0 ppm are thought to be bis- and tris-phosphorylated glycerol. We then attempted to determine what the signal at - 3.94 ppm corresponded to, see Supplementary Fig. 33. Yields based on **11** and determined by relative integration of the ^{31}P NMR signals.

Supplementary Table 7 Phosphorylation of glycerol **11** with PSO_3^{3-} under various conditions

Entry	Spectrum	Glycerol **11**	PSO_3^{3-}	Activator	Time	Temp.	Yield	
							Glycerol-1-phosphate **19**	Glycerol-2-phosphate **20**
1	Supp. Fig. 32, Spectrum A	50 mM	100 mM	Acrylonitrile (200 mM)	3 h	70 °C	~30%	~11%
2	Supp. Fig. 34, Spectrum A	50 mM	100 mM	–	10 h	70 °C	~27%	~9%
3	Supp. Fig. 35, Spectrum C	50 mM	100 mM	Acrylonitrile (200 mM)	7 d	RT	~25%	~6%
4	Supp. Fig. 36, not shown	50 mM	50 mM	$Fe(CN_6)^{3-}$ (100 mM)	7 h	RT	~20%	~6%
5	Supp. Fig. 36, Spectrum A	50 mM	100 mM	$Fe(CN_6)^{3-}$ (200 mM)	7 h	RT	~30%	~8%
6	Supp. Fig. 37, Spectrum A	50 mM	100 mM	UV light	15 h	~ 40 °C[a]	~28%	~8%

[a] - the temperature unavoidably rises in the UV apparatus used
RT - room temperature

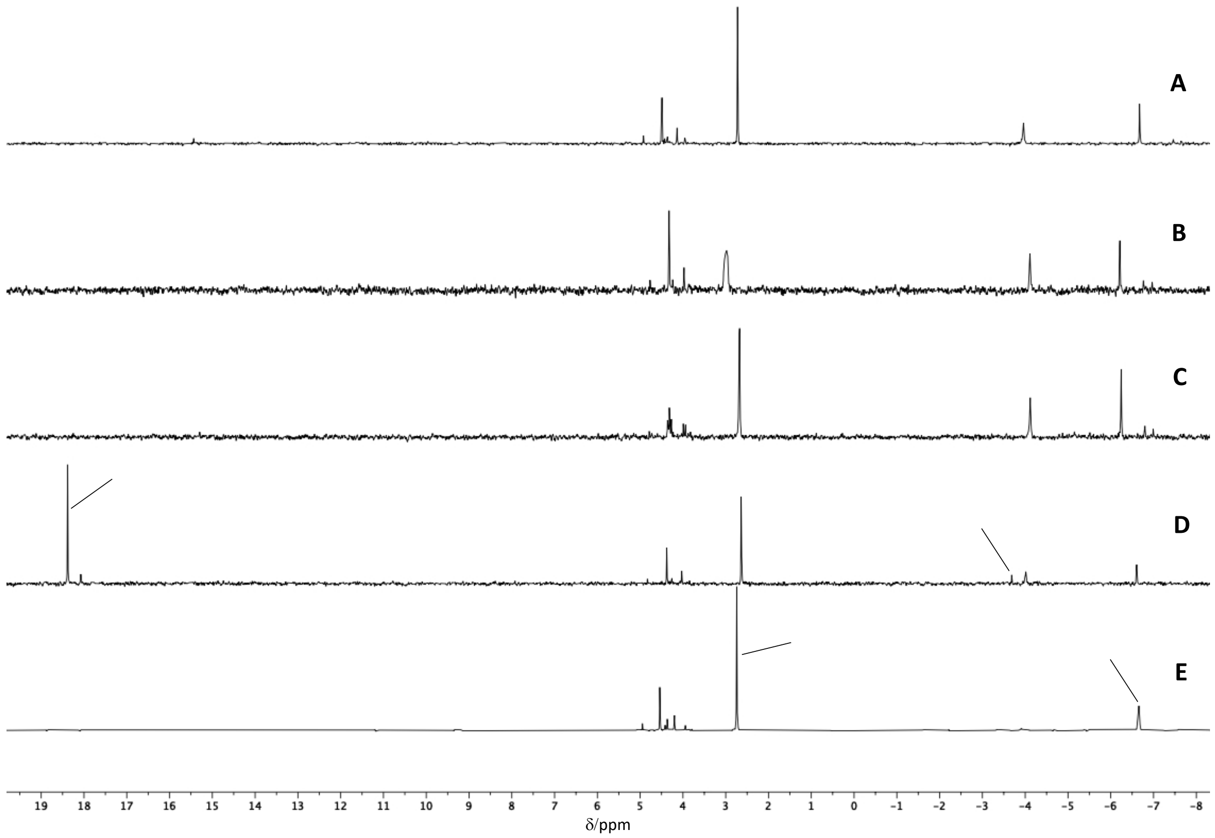

Supplementary Fig. 33 Phosphorylation of glycerol **11** using PSO_3^{3-} and acrylonitrile. A – ^{31}P NMR Spectrum after 3 h of reaction carried out according to Procedure 3 at 70 °C; B – As spectrum A, after being adjusted to pH ~ 13. The peak at – 3.94 ppm was first assumed to be symmetrical monothiopyrophosphate, resulting from the addition of one molecule of thiophosphate through the sulfur anion onto another molecule of activated thiophosphate with loss of RS^-, but the chemical shift does not match that reported for symmetrical monothiopyrophosphate (~ 15 ppm at pH = 13[45]); C – As spectrum B, except a ^{31}P-^{1}H coupling experiment was run. As the signal is not split in this spectrum, it would appear this species is not attached to glycerol or acrylonitrile, but to rule out glycerol-1,3-cyclic phosphate unequivocally the standard was made and spiked into the NMR sample; D – As spectrum C, after being spiked with a mixture of glycerol-1,3-cyclic phosphate (- 3.65 ppm) and glycerol-1,2-cyclic phosphate (18.5 ppm, see Reference 1 for synthesis); E – As spectrum A, but heating was continued for 20 h whereupon the signal at - 3.94 ppm had almost been consumed and the peaks corresponding to orthophosphate and pyrophosphate had increased. Additionally, the compound does not seem to be related to acrylonitrile as it appears to form in the absence of acrylonitrile (Supplementary Fig. 37).

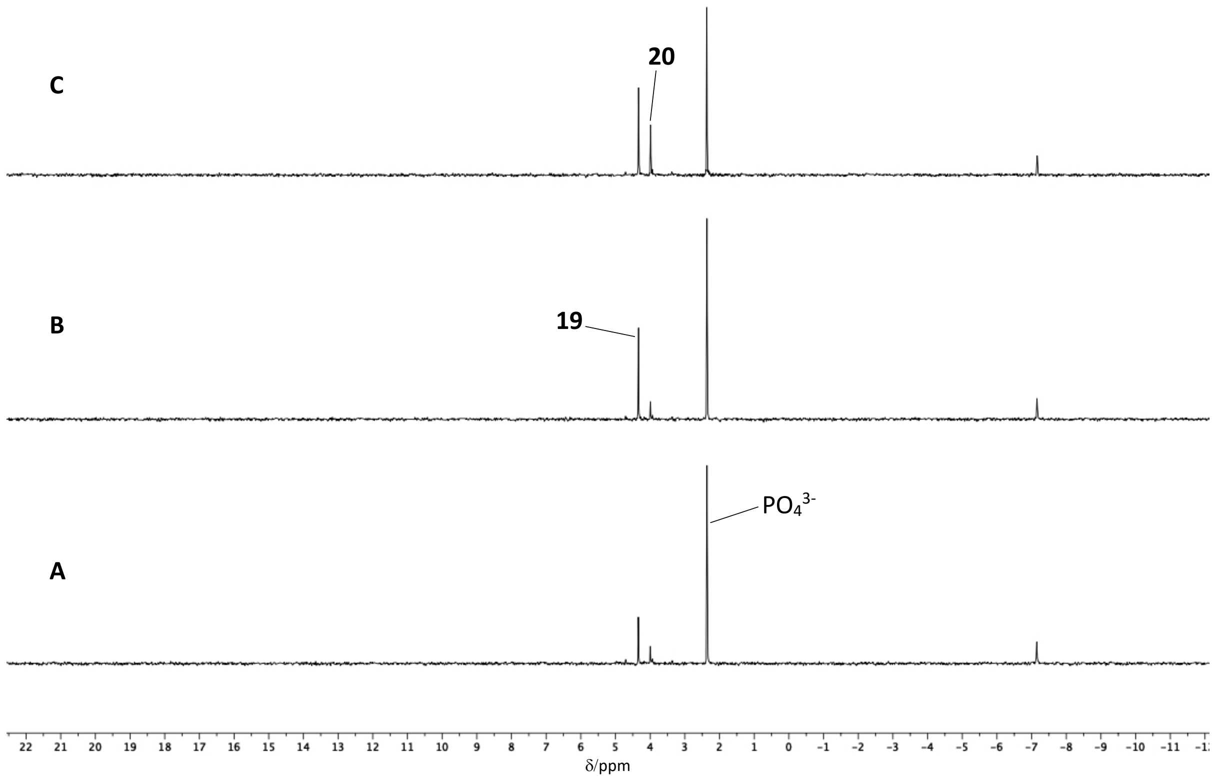

Supplementary Fig. 34 Phosphorylation of glycerol **11** using PSO_3^{3-} in the absence of acrylonitrile. A – ^{31}P NMR Spectrum after 10 h of reaction carried out according to Procedure 3 at 70 °C (glycerol-1-phosphate **19** ~ 27% yield and glycerol-2-phosphate **20** in ~ 9% yield); B – As spectrum A, after being spiked with a commercial sample of glycerol-1- phosphate **19**; C – As spectrum B, after being spiked with a commercial sample of glycerol-2- phosphate **20**. Slight changes in chemical shift are due to minor variations in pH upon addition of the standards. The peak at - 7.15 ppm is due to pyrophosphate. Other minor signals from 3.5 ppm to 5.0 ppm are thought to be bis- and tris-phosphorylated glycerol. Yields based on **11** and determined by relative integration of the ^{31}P NMR signals.

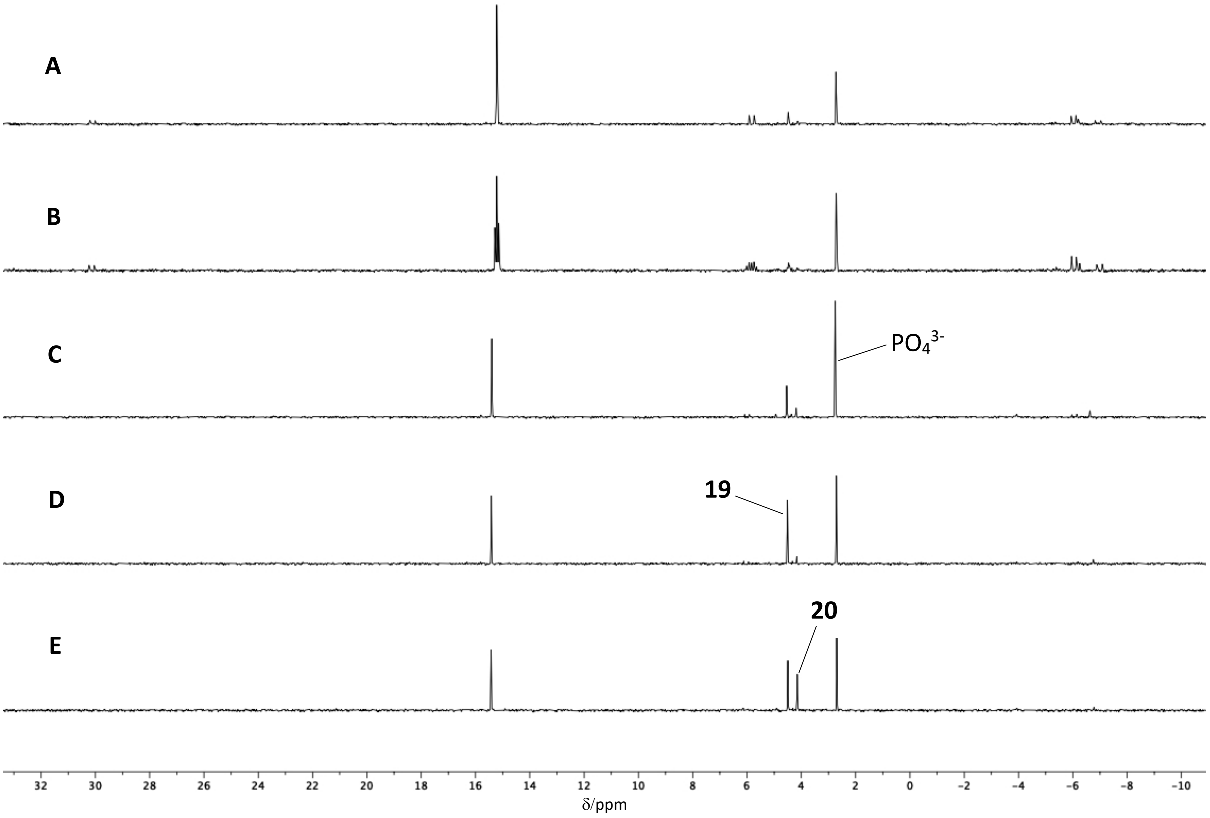

Supplementary Fig. 35 Phosphorylation of glycerol **11** using PSO_3^{3-} at room temperature in the presence of acrylonitrile. A – ^{31}P NMR Spectrum after 3 days of reaction carried out according to Procedure 3; B – As spectrum A using ^{31}P-^{1}H coupled experiment. The singlet which was at 15.2 ppm in spectrum A has been split into a triplet (J = 10.9), indicating it is *S*-2-cyanoethyl thiophosphate resulting from attack of thiophosphate on acrylonitrile. In what were a pair of doublets in spectrum A [δ 5.83 (J = 29.3), – 6.03 (J = 29.1)], one of the signals is now split into a doublet of triplets in spectrum B [δ 5.83 (J = 29.3, 14.8), - 6.04 (J = 29.3)], suggesting this is 2-cyanoethyl *S*-thiolodiphosphate - the product of *S*-2-cyanoethyl thiophosphate adding to another activated phosphate species - and shows good correlation with the ^{31}P NMR data of a related structure.[46] The other pair of doublets in spectrum A [d 30.1 (J = 31.7), - 6.95 (J = 32.0)] we assign to unsymmetrical monothiopyrophosphate, although it shows a slight perturbation from the reported ^{31}P NMR data,[45] the literature data was acquired under different conditions (pure D_2O, pH 13.1); C – As spectrum A, after 1 week of reaction according to Procedure 3 (glycerol-1-phosphate **19** ~ 25% yield and glycerol-2-phosphate **20** in ~ 6% yield); D – As spectrum C, after being spiked with a commercial sample of **19**; E – As spectrum D, after being spiked with a commercial sample of **20**. The other minor signals from 4.0 – 4.8 ppm are thought to be bis- and tris-phosphorylated glycerol. Yields based on **11** and determined by relative integration of the ^{31}P NMR signals.

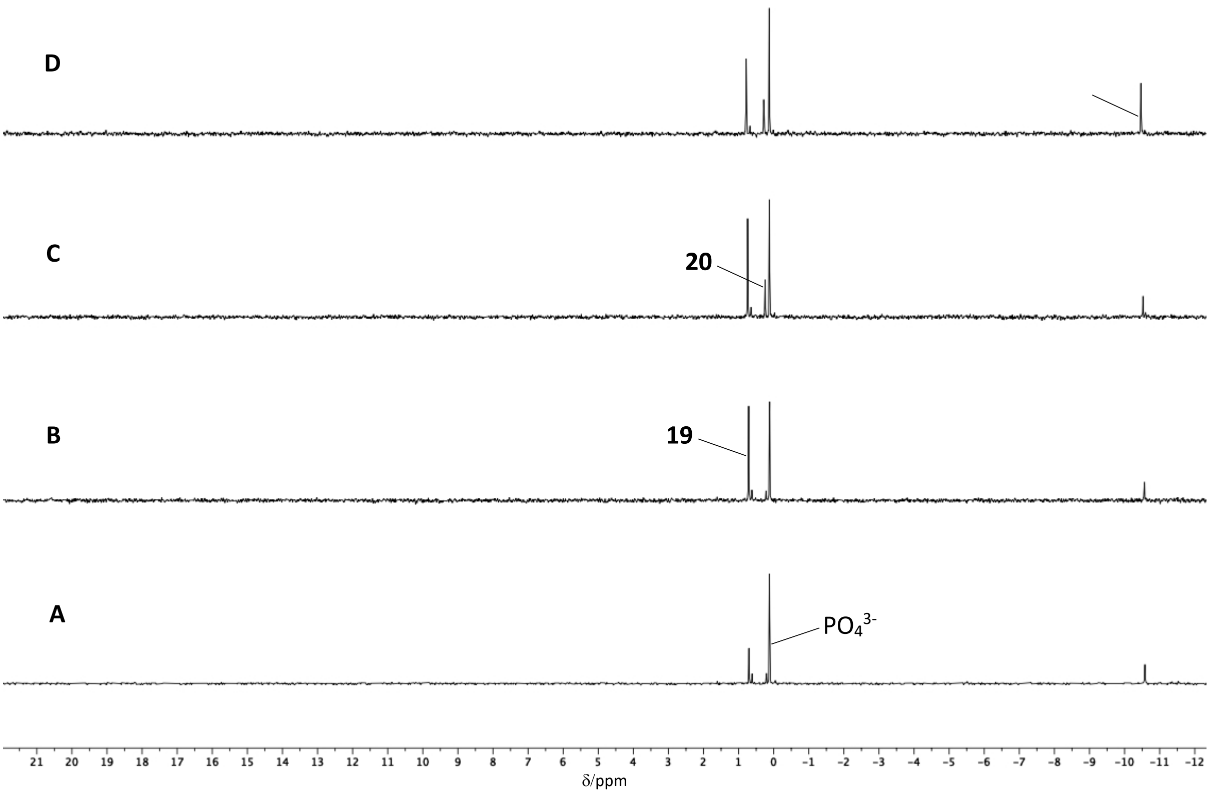

Supplementary Fig. 36 Phosphorylation of glycerol **11** by activation of PSO_3^{3-} with ferricyanide. Dibasic thiophosphate was prepared according to General procedure 1 (0.050 mmol or 0.100 mmol). Glycerol **11** (4.6 mg, 0.050 mmol) was dissolved in degassed formamide (1 mL) and added to the thiophosphate. Ferricyanide (2 equiv. relative to thiophosphate) was then added, and the reaction agitated before a sample was removed, diluted with D_2O and examined by ^{31}P NMR spectroscopy. A – ^{31}P NMR Spectrum after 7 h reaction using 100 mM thiophosphate (~ 20% of glycerol-1- phosphate **19** and ~ 6% of glycerol-2-phosphate **20** for 50 mM PSO_3^{3-} (not shown) or ~ 30% of **19** and ~ 8% of **20** for 100 mM PSO_3^{3-}); B – As spectrum A, after being spiked with a commercial sample of **19**; C – As spectrum B, after being spiked with a commercial sample of **20**; D – As spectrum C, after being spiked with a commercial sample of pyrophosphate. The other minor signals from – 0.1 ppm to 0.6 ppm are thought to be bis- and tris-phosphorylated glycerol. Ferrocyanides ($Fe(CN_6)^{4-}$) are heavily implicated in our scheme,[1,8,9,10,36] and these complexes are easily oxidised to ferricyanides ($Fe(CN_6)^{3-}$) by UV light.[47] Yields based on **11** and determined by relative integration of the ^{31}P NMR signals.

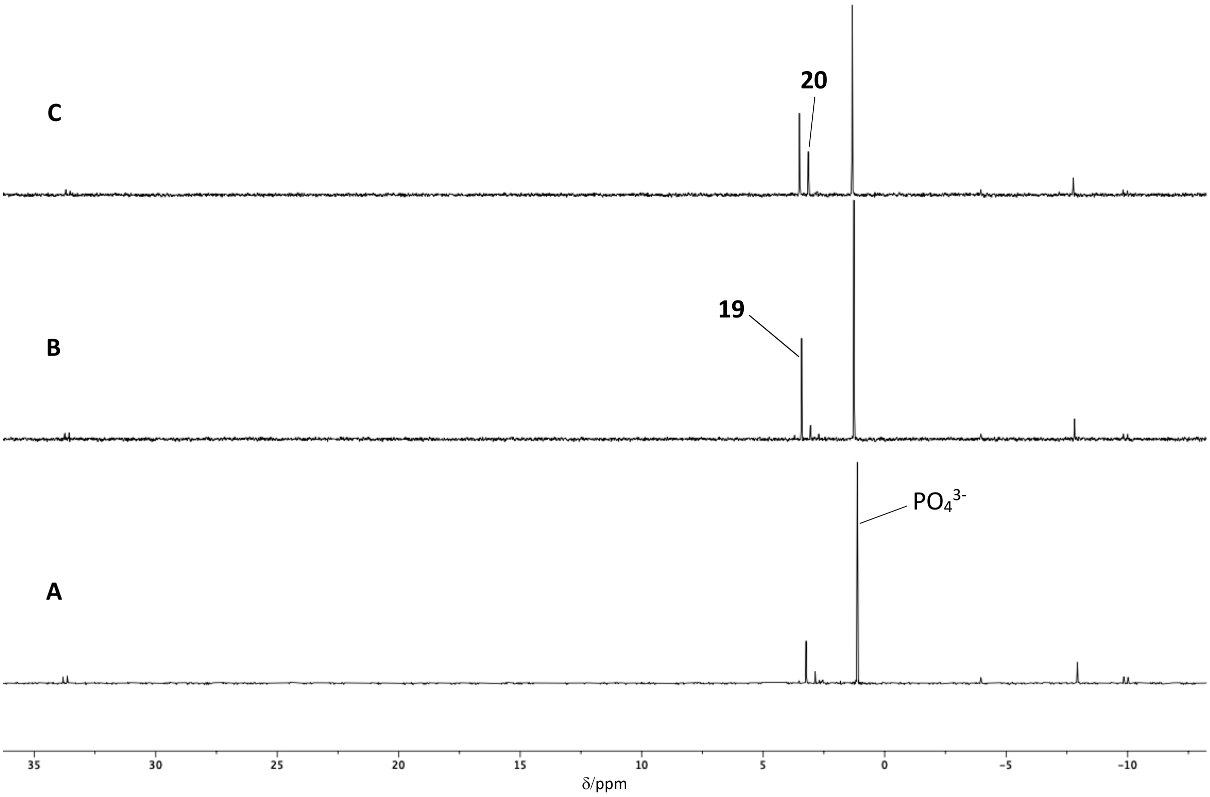

Supplementary Fig. 37 Phosphorylation of glycerol **11** by irradiation of PSO_3^{3-}. Dibasic thiophosphate was prepared according to General procedure 1 (0.100 mmol). Glycerol **11** (4.6 mg, 0.050 mmol) was dissolved in degassed formamide (1 mL), added to the thiophosphate and then the solution was transferred to a quartz cuvette. The reaction was sealed and irradiated for the desired amount of time before a sample was removed, diluted with D_2O and examined by ^{31}P NMR spectroscopy. A – ^{31}P NMR Spectrum after 15 h of reaction (~ 28% of glycerol-1-phosphate **19** and ~ 8% of glycerol-2-phosphate **20**); B – As spectrum A, after being spiked with a commercial sample of **19**; C – As spectrum B, after being spiked with a commercial sample of **20**. The other minor signals from 1.8 ppm to 3.5 ppm are thought to be bis- and tris-phosphorylated glycerol. The compound which gives a signal at - 3.94 ppm has been discussed in Supplementary Fig. 33. The pair of doublets (33.7 ppm and - 9.85 ppm, J = 28.5) are thought to be due to unsymmetrical monothiopyrophosphate and correlate reasonably well with the literature values (30.8 ppm and - 5.98 ppm, J = 30, in D_2O, pH = 13.1)[45], there may be some discrepancy due to the difference in pH and that our samples contained 20-30% formamide. The peak at - 7.95 ppm is due to pyrophosphate. The chemical shift was altered slightly by a change in pH upon addition of the standards. Yields based on **11** and determined by relative integration of the ^{31}P NMR signals.

Procedure 4

Glycolaldehyde **2** (0.050 mmol, 3 mg) and $Na_3PSO_3.xH_2O$ (purity and water content predetermined and accounted for, 0.200 mmol) were dissolved in an Eppendorf tube in degassed 10% D_2O in H_2O (1 mL) and NH_4Cl (16 mg, 0.300 mmol) and $NaH_2PO_4.2H_2O$ (if used, 16 mg, 0.100 mmol) were added. The pH was adjusted to 9.2, or 7.0 if phosphate was used, then the volume was made up to 2 mL with degassed 10% D_2O in H_2O. The solution was transferred to a quartz cuvette, sealed and the reaction was irradiated for the desired amount of time after which an aliquot was analysed by ^1H NMR spectroscopy.

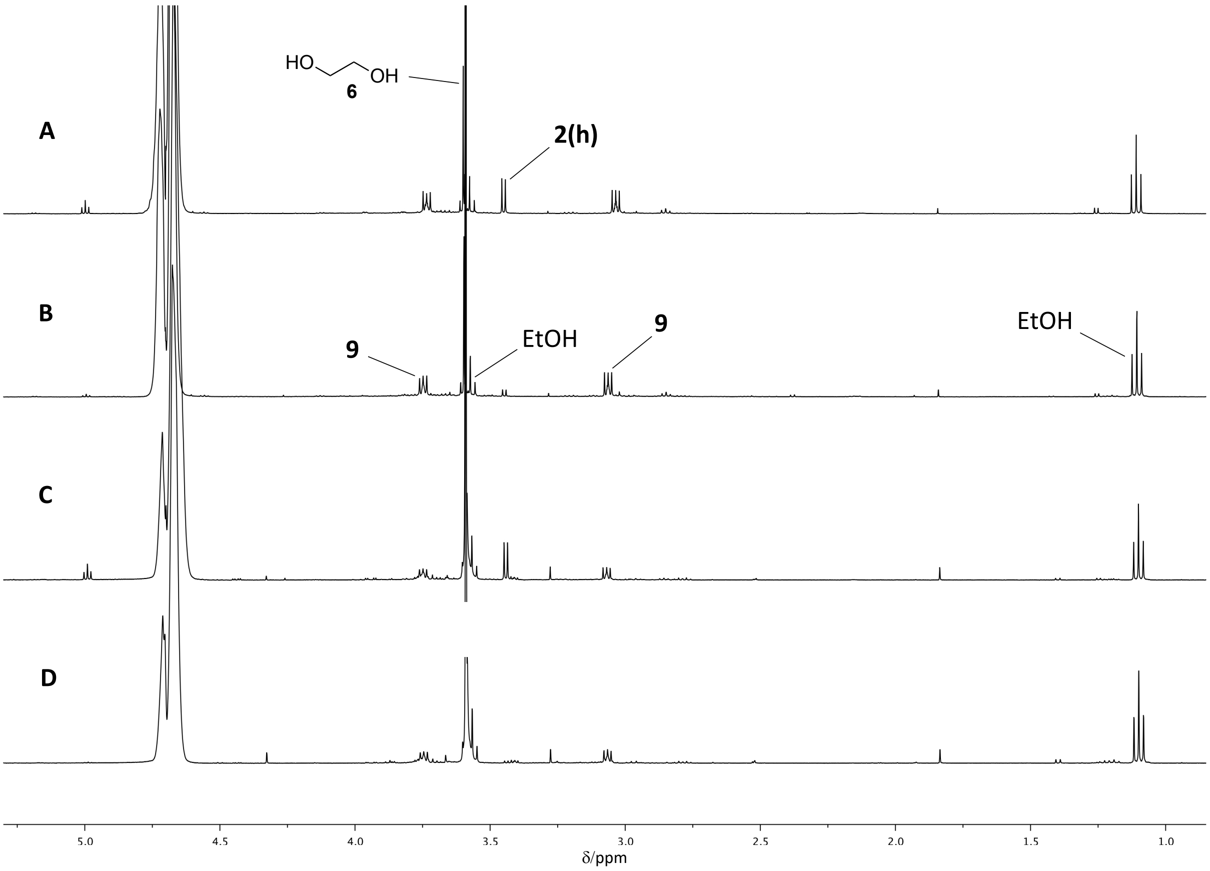

Supplementary Fig. 38 Synthesis of ethanolamine **9** from glycolaldehyde **2**, in the presence and absence of phosphate. A – ^1H NMR Spectrum after 2 h reaction according to Procedure 4 at pH 9.2; B – As spectrum A, after 6 h reaction; C – ^1H NMR Spectrum after 2 h reaction according to Procedure 4 with the inclusion of phosphate at pH 7.0; D – As spectrum C, after 6 h reaction. Yields estimated by addition of disodium fumarate (50 mM solution, 50 μL into 450 μL of crude reaction) as a standard and relative integration of ^1H NMR signals.

Supplementary Table 8 Reductive amination of glycolaldehyde **2** with NH_3

Entry	Spectrum	Glycolaldehyde **2**	PSO_3^{3-}	NH_4Cl	PO_4^{3-}	Irradiation time	Yields			
							Ethanolamine **9**	Ethylene glycol **6**	EtOH	Glycolaldehyde[a] **2**
1	Supp. Fig. 38, Spectrum A	25 mM	100 mM	150 mM	–	2 h	~15%	~8%	~13%	~10%
2	Supp. Fig. 38, Spectrum B	25 mM	100 mM	150 mM	–	6 h	~14%	~11%	~16%	~2%
3	Supp. Fig. 38, Spectrum C	25 mM	100 mM	150 mM	50 mM	2 h	~5%	~78%	~8%	~6%
4	Supp. Fig. 38, Spectrum D	25 mM	100 mM	150 mM	50 mM	6 h	~6%	~82%	~12%	trace

[a] - yield of **2** corresponds to the amount remaining after irradation
trace - 1% or less

Procedure 5

To an Eppendorf tube was charged NH_4Cl (8 mg, 0.150 mmol), $NaH_2PO_4.2H_2O$ (8 mg, 0.050 mmol) and $Na_3PSO_3.xH_2O$ (purity and water content predetermined and accounted for, 0.100 mmol), then degassed 10% D_2O in H_2O (1 mL) was added. The pH was adjusted to 7.0, the volume was made up to 2 mL with degassed 10% D_2O in H_2O and glycolonitrile **1** (4.9 mL, 0.050 mmol) was added. The solution was transferred to a quartz cuvette, sealed and the reaction was irradiated for 1.5 h.

From this solution, 1 mL was removed and added to an Eppendorf tube containing cyanamide (2 mg, 0.050 mmol), which was then sealed and heated to 50 °C for 20 h. An aliquot (450 μL) was removed and transferred to an NMR spectroscopy tube and an aqueous solution of fumarate (50 mM, 50 μL) was added as an internal standard.

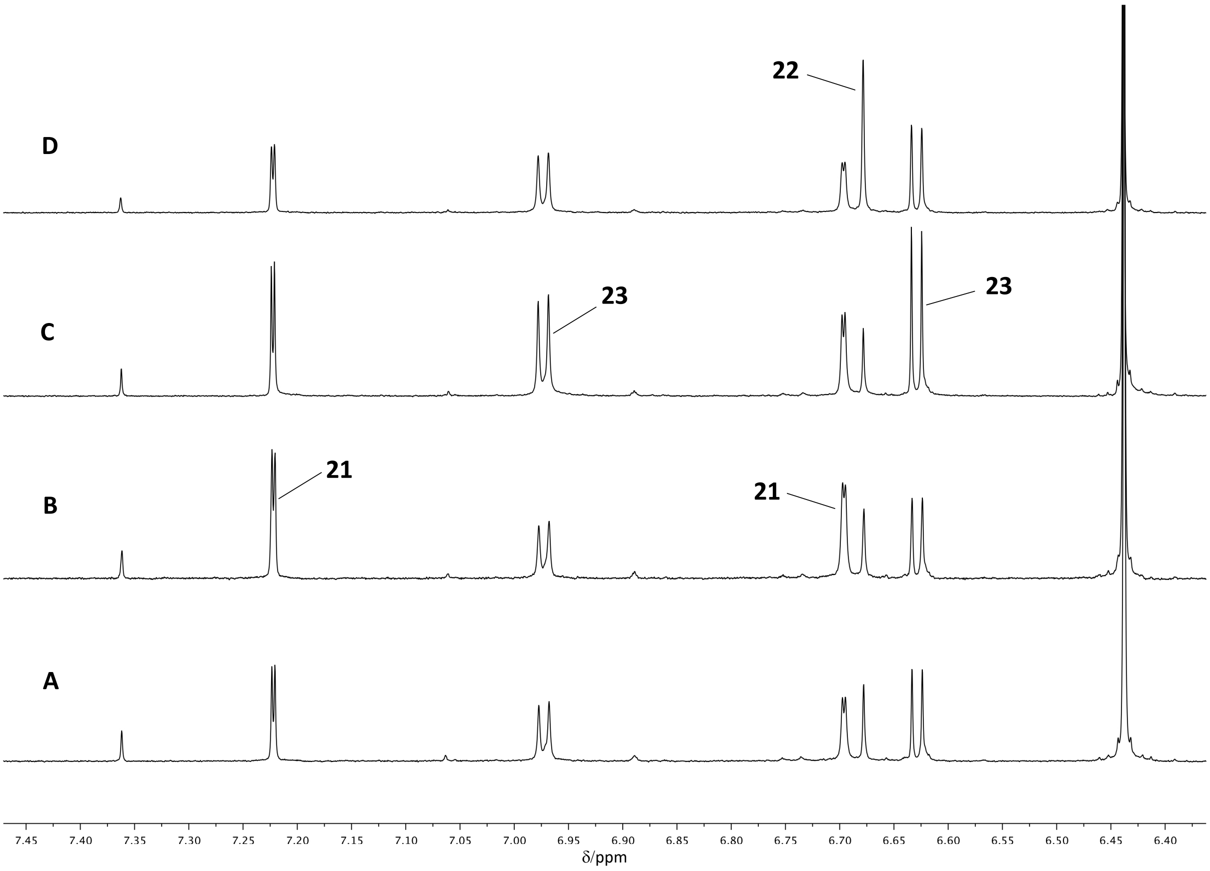

Supplementary Fig. 39 Prebiotic synthesis of ethanolamine **9**, 2-aminooxazole **21**, 2-aminoimidazole **22** and 2-aminothiazole **23** from glycolonitrile **1** in one pot (**14** not shown). A – ^{1}H NMR Spectrum of the crude reaction mixture after 20 h heating according to Procedure 5; B – As spectrum A, spiked with a commercial sample of **21**; C – As spectrum B, spiked with a commercial sample of **23;** D – As spectrum C, spiked with a commercial sample of **22**. Yields estimated by addition of disodium fumarate (50 mM solution, 50 μL into 450 μL of crude reaction) as a standard (singlet at 6.43 ppm) and relative integration of ^{1}H NMR signals.

Supplementary Table 9 One-pot synthesis of azoles

Entry	Spectrum	Glycolonitrile **1**	PSO_3^{3-}	NH_4Cl	PO_4^{3-}	Irradiation time	Yields[a]			
							Ethanolamine **9**	2-Aminooxzole **21**	2-Aminoimidazole **22**	2-Aminothiazole **23**
1	Supp. Fig. 39, Spectrum A	25 mM	50 mM	75 mM	25 mM	1.5 h	~1%	~8%	~2%	~9%
2	Supp. Fig. 40, Spectrum A	25 mM	25 mM	–	25 mM	1.5 h	–	~16%	–	~2%
3	Supp. Fig. 40, Spectrum B	25 mM	75 mM	–	25 mM	40 min	–	~7%	–	~8%

[a] - yields based on **1** and were obtained after heating the reduction products with NH_2CN for 20 h (see Supplementary Figs. 39 and 40 and Procedure 5)

Although the yield of **9** was low, its accumulation may be expected given the high boiling point, unless undergoing other reactions. This is in line with Orgel's synthesis of oligonucleotides from *ribo*-nucleoside-2′,3′-cyclic phosphates, which requires the inclusion of **9** and evaporation of H_2O to the point of dryness[25].

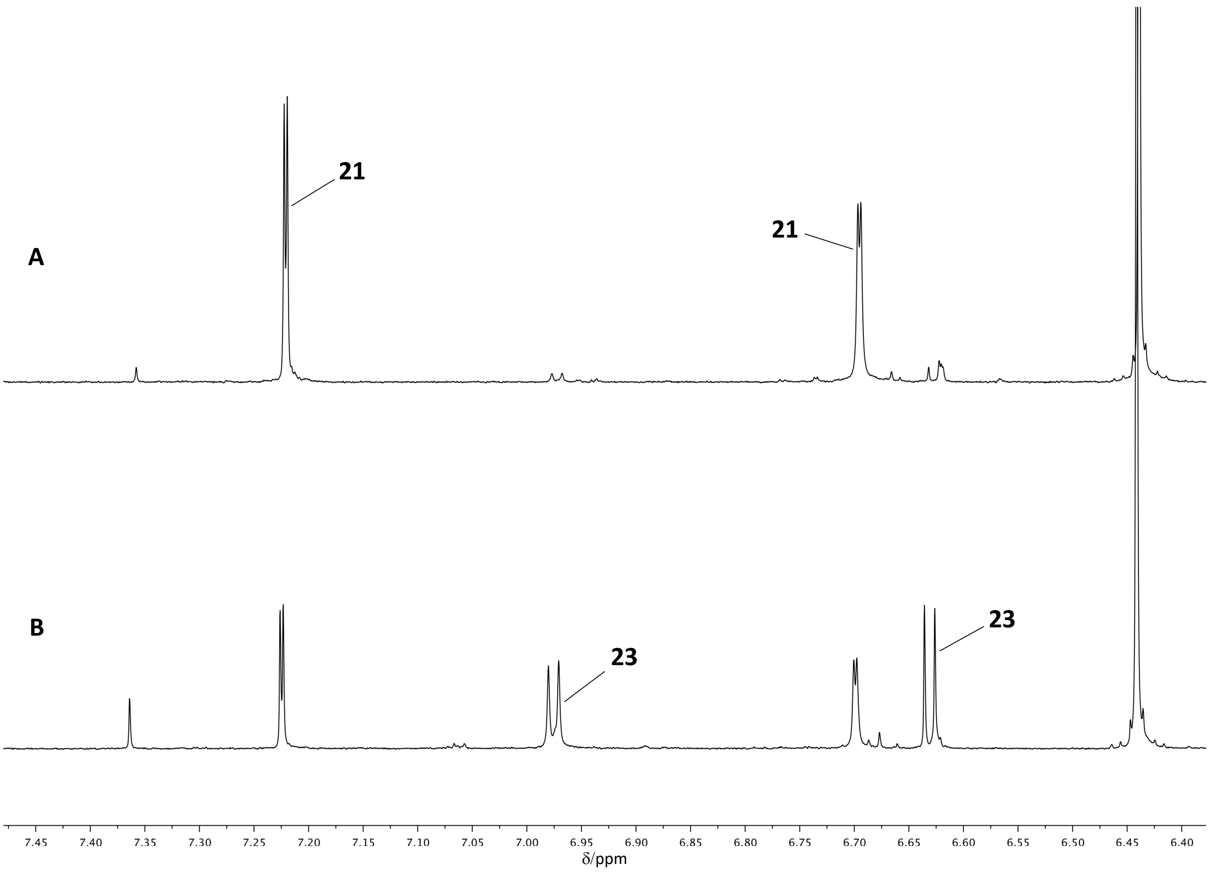

Supplementary Fig. 40 Prebiotic synthesis of 2-aminooxazole **21** and 2-aminothiazole **23** from glycolonitrile **1** in one pot. A – ^{1}H NMR Spectrum of the crude reaction mixture after 20 h heating according to Procedure 5, except no NH_4Cl was added and only 1 equivalent of $Na_3PSO_3.xH_2O$ was used. After 1.5 h irradiation, there was ~ 36% glycolaldehyde **2** present (not shown); B – As spectrum A, except 3 equivalents of $Na_3PSO_3.xH_2O$ were used. After 40 min irradiation there was ~ 32% glycolaldehyde **2** present (not shown). Yields estimated by addition of disodium fumarate (50 mM solution, 50 μL into 450 μL of crude reaction) as a standard (singlet at 6.43 ppm) and relative integration of ^{1}H NMR signals.

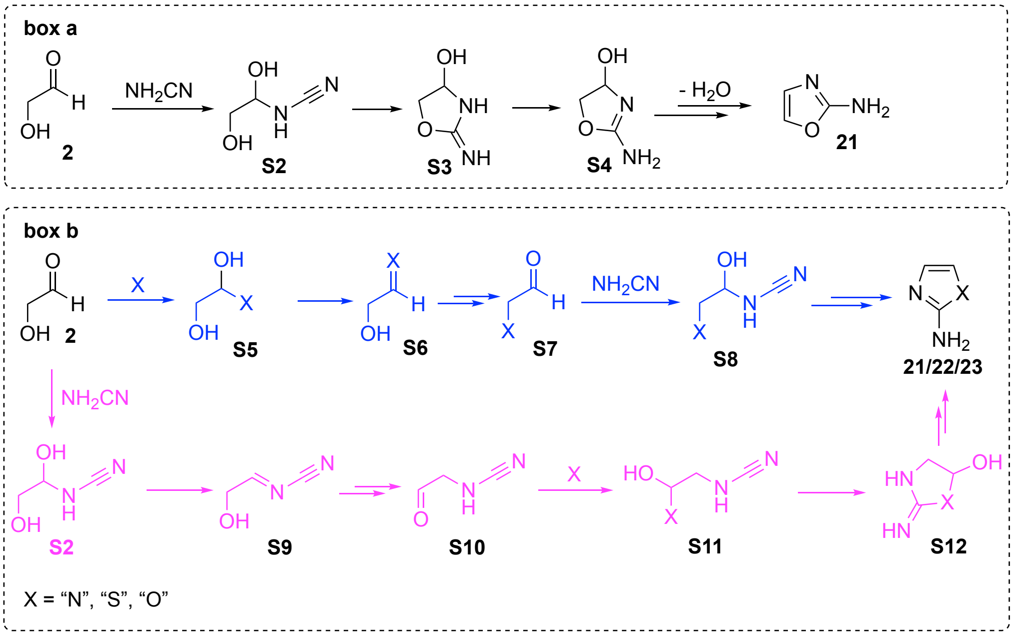

Supplementary Fig. 41 Mechanistic considerations for the production of azoles **21**, **22** and **23** from glycolaldehyde **2** and cyanamide in the presence NH_3 and H_2S in H_2O. When the prebiotic synthesis of 2-aminooxazole **21** was first reported from the condensation of **2** with cyanamide, a mechanism which essentially follows the route depicted in **box a** was suggested.[26] However, after it was shown that 2-aminoimidazole **21** can be formed by inclusion of NH_4Cl with **2** and cyanamide it was clear that a different route must be available which operates in tandem with, or instead of, that shown in **box a.**[48] Similarly, to form 2-aminothiazole **23**, the mechanism in **box a** will not suffice. There are two possible alternative mechanisms which are outlined in **box b**, the difference being that either the nucleophile (XH_n) adds first (blue pathway) or cyanamide adds first (magenta pathway). Following the blue pathway from **S5**, H_2O is expelled to give the C=XH_{n-2} bond in **S6**, this compound can undergo a Lobry de Bruyn-Alberda van Ekenstein rearrangement in the case that XH_n = OH_2 and an Amadori rearrangement in the case that XH_n = NH_3 to give **S7** (see below for XH_n = SH_2). Addition of cyanamide then allows cyclisation of XH_{n-1} onto the nitrile carbon of **S8** and after dehydration and aromatisation results in **21/22/(23)**. Following the magenta pathway, cyanamide adds to **2** giving **S2** and this species extrudes H_2O to give **S9** which undergoes Amadori rearrangement to aldehyde **S10**. Addition of the nucleophile XH_n then gives **S11** from which XH_{n-1} or C1-OH can cyclise onto the nitrile carbon, eventually leading to **21/22/23**. Whilst the blue pathway may be reasonable in the case of **22**, to arrive at **23** *via* this route would require the formation of a thiocarbonyl using nothing more than general acid-base catalysis, which seems unlikely considering the usual requirement for strong Bronsted/Lewis acid catalysis.

Procedure 6

A solution of 2-aminoimidazole sulfate (2.5 mg, 0.019 mmol), 2-aminooxazole **21** (3 mg, 0.050 mmol), 2-aminothiazole **23** (6 mg, 0.060 mmol), ethanolamine **9** (0.36 μL, 0.006 mmol) and $NaH_2PO_4.2H_2O$ (8 mg, 0.050 mmol) in H_2O/D_2O (0.7/0.1 mL) was adjusted to pH 6.5 and the volume made up to 1 mL with H_2O. Glyceraldehyde **6** (4.5 mg, 0.050 mmol) was added, the tube sealed and heated to 40 °C for the desired amount of time.

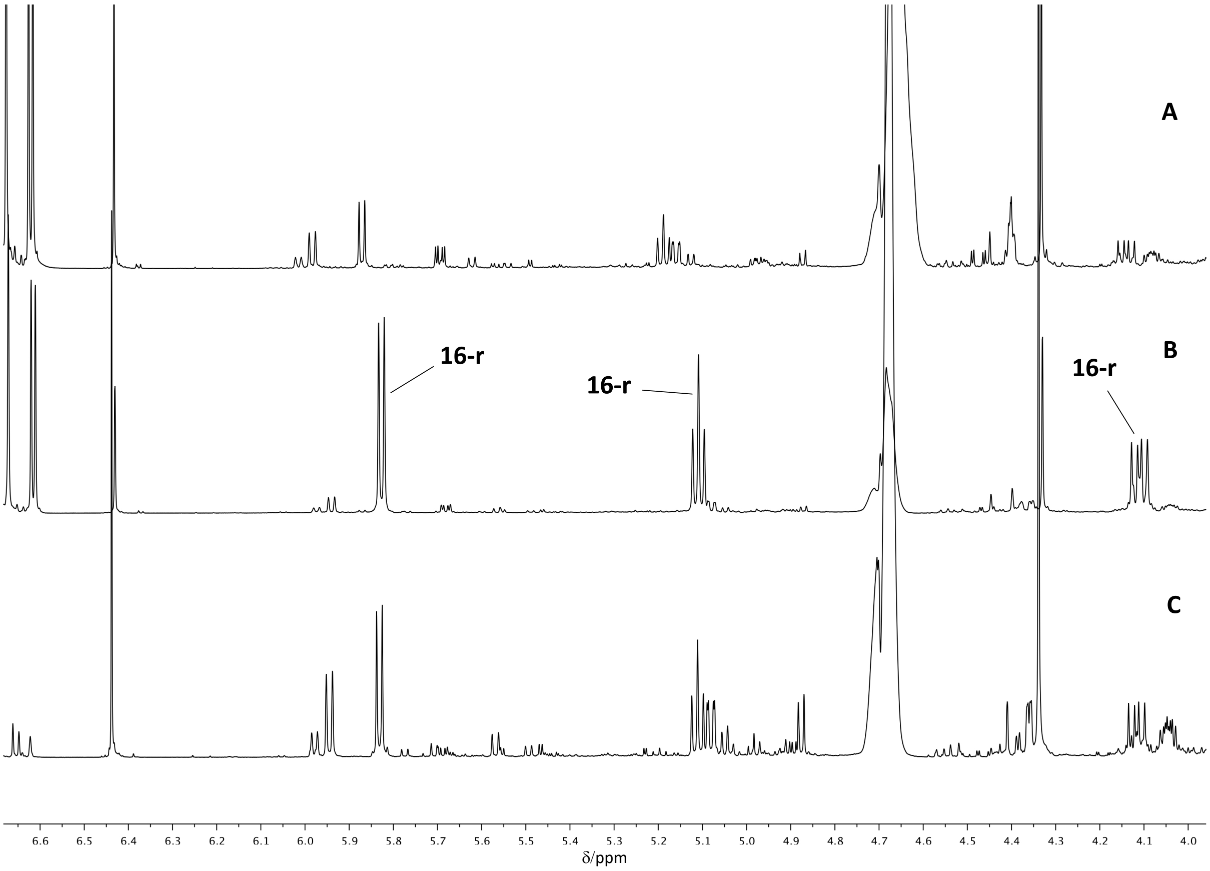

Supplementary Fig. 42 Prebiotic synthesis of pentose aminooxazolines **16** from glycolonitrile **1** in one pot. A – ^{1}H NMR Spectrum of the crude reaction mixture after 20 h heating according to Procedure 6; B – As spectrum A, spiked with an authentic sample of *ribo*-aminooxazoline (**16-r**); C – ^{1}H NMR Spectrum of the crude reaction mixture after 20 h heating according to Procedure 6 except 2-aminoimidazole sulfate, 2-aminothiazole **23** and ethanolamine **9** were not added. Although the yields of **16** in the original report were > 80%, the concentration of reagents used here are approximately an order of magnitude lower.[26]

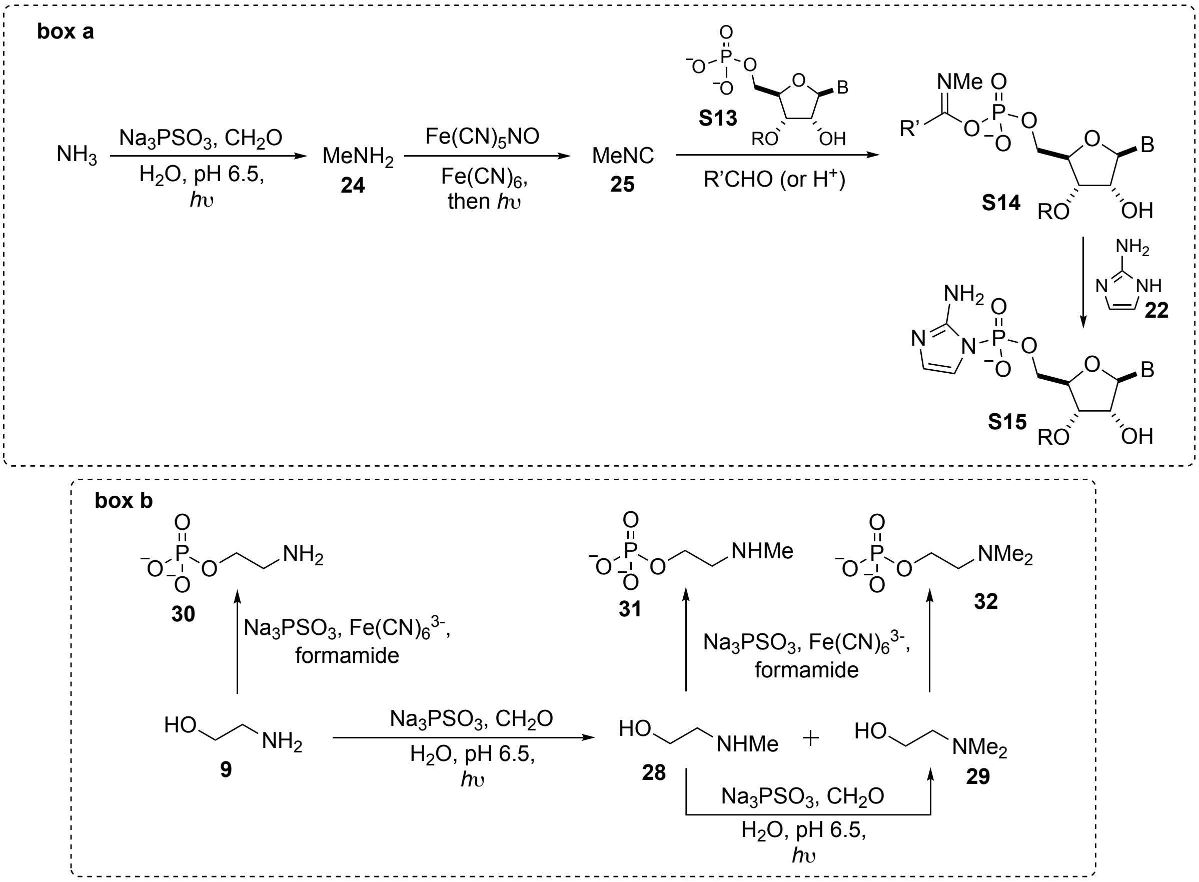

Supplementary Fig. 43 Synthesis of $MeNH_2$ **24** and methylated ethanolamine derivatives **28** and **29** from ethanolamine **9**. **box a** – the reductive methylation of ammonia provides **24** (this work, see main text) and we have previously shown how simple amines such as **24** can be converted into isonitriles *e.g.* **25**, by ferrocyanide and nitroprusside, also expected products of the cyanosulfidic scenario we have discussed before.[1,7] We,[7,8] and others,[30] have shown how isonitriles can be activated for nucleophilic attack by nucleotides **S13** by an aldehyde or mildly acidic conditions. The imidoyl phosphate intermediate **S14** is unstable and can be subject to attack by imidazoles, such as 2-aminoimidazole **22**, giving phosphoroimidazolides **S15**. These can be 5'-mononucleotides, activated for incorporation into a primer,[29] or a 5'-terminus of an oligonucleotide activated for ligation;[30] **box b** – reductive methylation of ethanolamine **9** sequentially gives **28** and **29**, further addition of formaldehyde **27** and reduction converts more **9** to **28** and **28** to **29**, potentially giving **29** exclusively. The amino alcohols **9**, **28** and **29** can then be phosphorylated with Na_3PO_3.

Procedure 7

Formaldehyde **27** (37%, 0.050 mmol, 3.8 μL), $Na_3PSO_3.xH_2O$ (purity and water content predetermined and accounted for, 0.150 mmol) and $Na_2HPO_4.7H_2O$ (if used, 27 mg, 0.100 mmol) were dissolved in an Eppendorf tube in degassed 10% D_2O in H_2O (1 mL) and NH_4Cl (16 mg, 0.300 mmol) was added. The pH was adjusted to 9.2 or 7.0 (if phosphate was included) using degassed HCl/NaOH, then the volume was made up to 2 mL with degassed 10% D_2O in H_2O. The solution was transferred to a quartz cuvette, sealed and the reaction was irradiated for the desired amount of time. An aliquot (450 μL) of the reaction was spiked with an aqueous solution of fumarate (50 mM, 50 μL) as an internal standard and a ^1H NMR spectrum was collected.

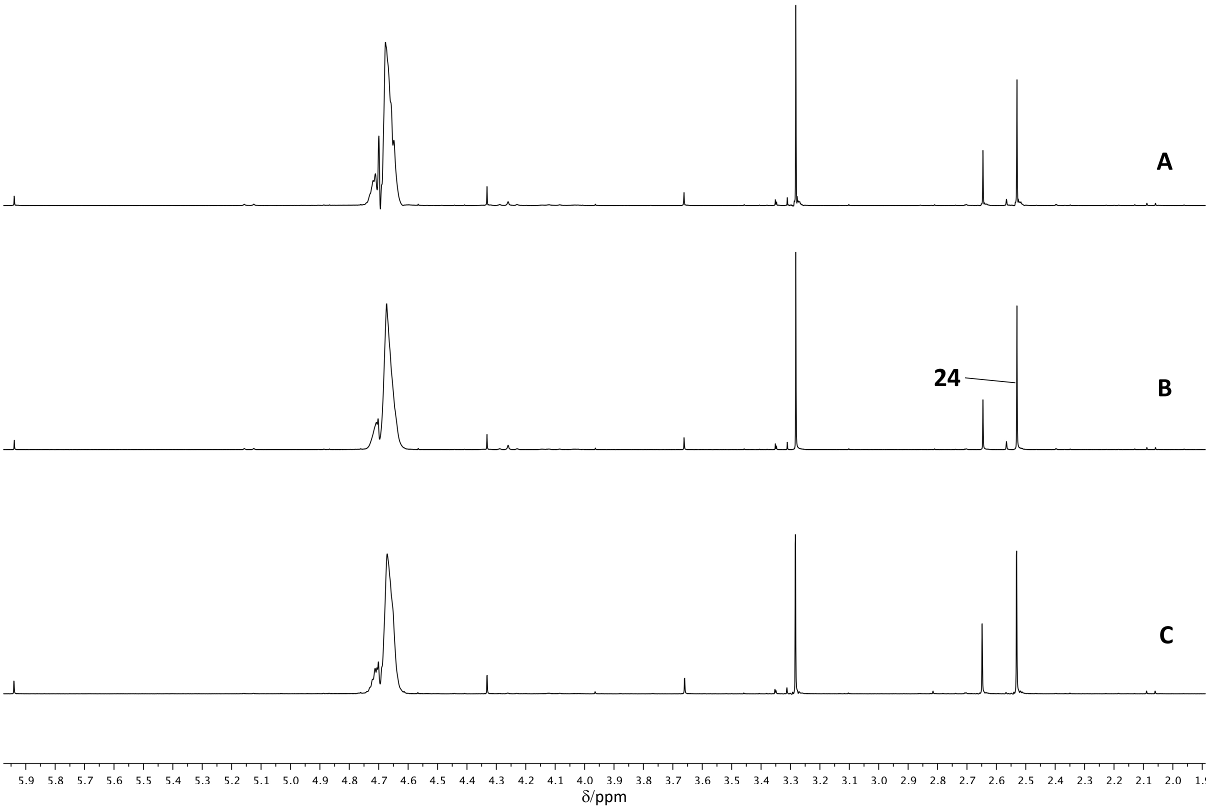

Supplementary Fig. 44 Prebiotic synthesis of methylamine **24**. A – ^1H NMR Spectrum of a reaction carried out according to Procedure 7 after 2 h irradiation at pH 9.2; B – As spectrum A, spiked with a commercial sample of **24**; C – ^1H NMR Spectrum of a reaction carried out according to Procedure 7 after 2 h irradiation at pH 7.0. Singlets observed at δ 3.28 ppm in all spectra are due to MeOH which is present in commercial formaldehyde **27** as a stabiliser and also results from direct reduction of **27**.

Supplementary Table 10 Reductive amination of CH_2O with NH_3

Entry	Spectrum	Amine	PSO_3^{3-}	PO_4^{3-}[a]	Irradiation time	CH_2O **27**	Yield
							$MeNH_2$ **24**
1	Supp. Fig. 44, Spectrum A	NH_4Cl (150 mM)	75 mM	–	2 h	25 mM	~11%
2	Supp. Fig. 44, Spectrum C	NH_4Cl (150 mM)	75 mM	50 mM	2 h	25 mM	~26%
3	Supp. Fig. 44, not shown	NH_4Cl (75 mM)	75 mM	–	2 h	25 mM	~10%
4	Supp. Fig. 44, not shown	NH_4Cl (75 mM)	75 mM	50 mM	2 h	25 mM	~10%

[a] - Reactions containing PO_4^{3-} were run at an initial pH of 7, otherwise at an initial of pH 9.2

Procedure 8

Ethanolamine **9** (0.060 mmol, 3.6 μL), formaldehyde **27** (37% wt., 0.180 mmol, 13.5 μL) and $Na_3PSO_3.xH_2O$ (purity and water content predetermined and accounted for, 0.240 mmol) were dissolved in an Eppendorf tube in degassed 10% D_2O in H_2O (1 mL), then the pH was adjusted to 7.0. The volume was made up to 2 mL with degassed 10% D_2O in H_2O and the solution was transferred to a quartz cuvette, sealed and the reaction was irradiated for 4 h. A ^1H NMR spectrum was then collected.

Formaldehyde **27** (37% wt., 0.040 mmol, 3.2 μL) and $Na_3PSO_3.xH_2O$ (purity and water content predetermined and accounted for, 0.080 mmol) were added and the pH re-adjusted to 7.0. Irradiation continued a further 2 h before another ^1H NMR spectrum was collected.

Formaldehyde **27** (37% wt., 0.040 mmol, 3.2 μL) was added and irradiation continued for a further 2 h after which time a ^1H NMR spectrum was collected.

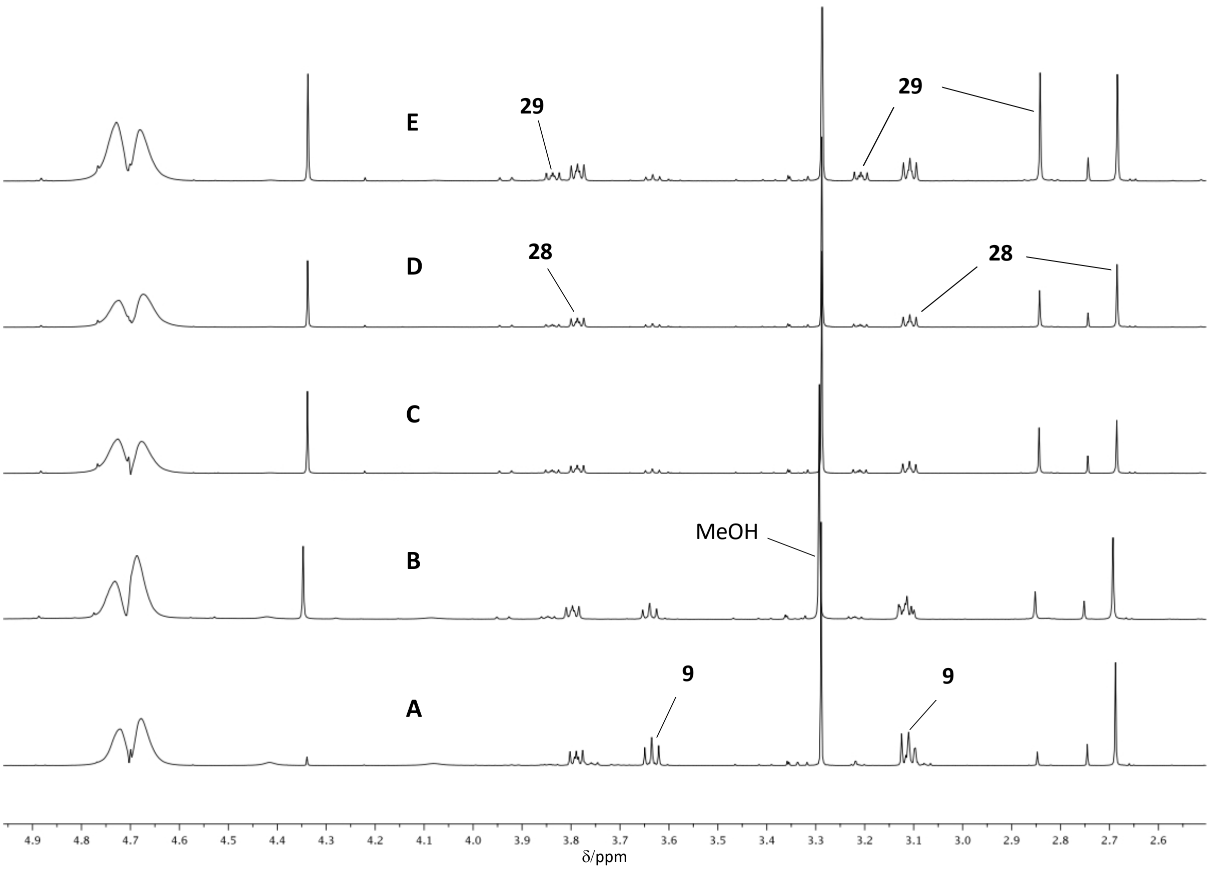

Supplementary Fig. 45 Prebiotic synthesis of *N*-methyl ethanolamine **28** and *N,N*-dimethyl ethanolamine **29** from ethanolamine **9**. A – ^1H NMR Spectrum of a reaction carried out according to Procedure 8 after 4 h irradiation; B – ^1H NMR Spectrum of a reaction carried out

according to Procedure 8 after 2 h irradiation after the 2nd addition of formaldehyde **27**; C – ^{1}H NMR Spectrum of a reaction carried out according to Procedure 8 after 2 h irradiation after the 3rd addition of **27**; D – As spectrum C, spiked with an authentic sample of **28**; E – As spectrum D, spiked with a commercial sample of **29**.

Procedure 9

The aminoalcohol **9**, **28** or **29** (0.050 mmol) and $Na_3PSO_3.xH_2O$ (purity and water content predetermined and accounted for, 0.050 mmol) were dissolved in an Eppendorf tube in degassed H_2O (0.8 mL) and the pH was adjusted to 7.0 – 7.2 with degassed HCl. The solution was frozen in liquid N_2 and lyophilised.

The solid was dissolved in degassed formamide (1 mL) and potassium ferricyanide (0.100 mmol, 33 mg) was added. The reaction was then agitated and a sample was taken at the desired timepoint and diluted with D_2O. NMR Spectroscopy was then used to analyse the reaction.

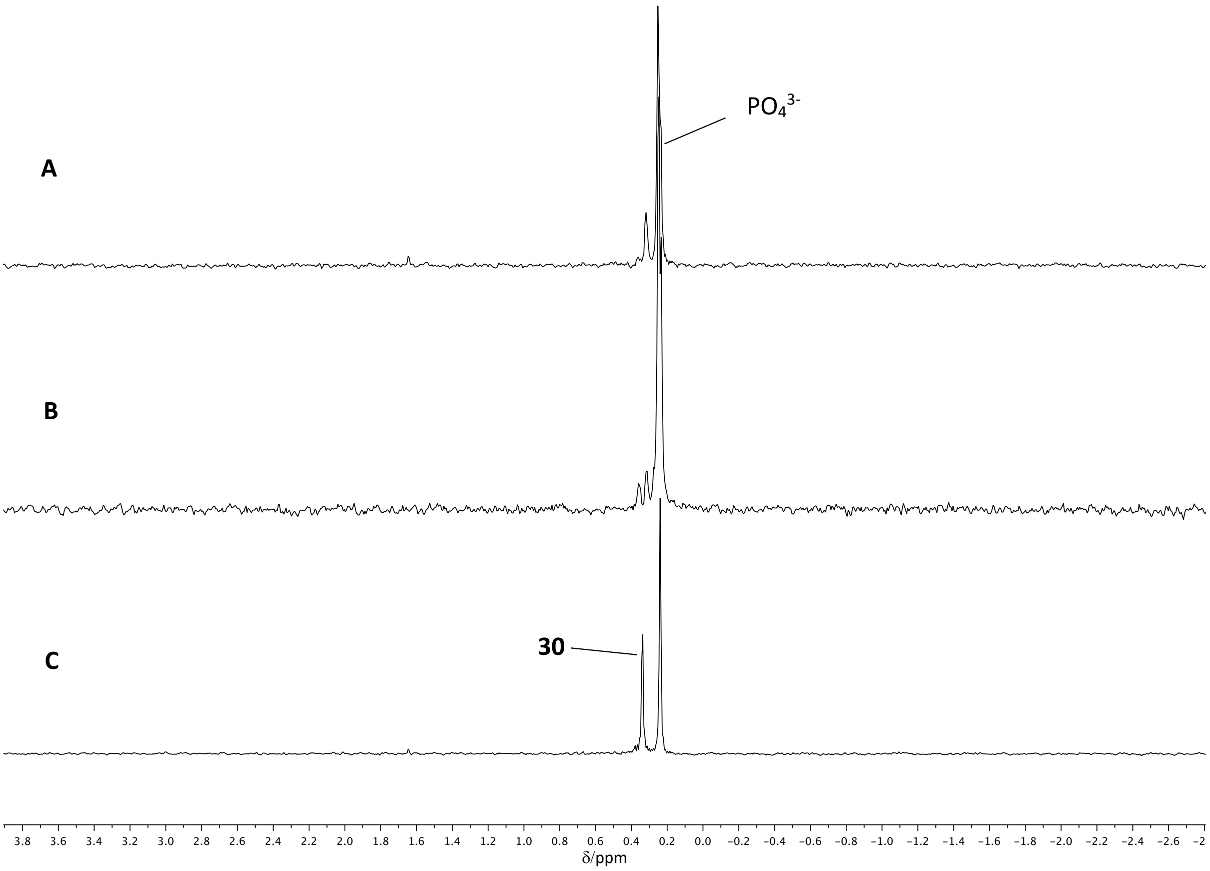

Supplementary Fig. 46 Phosphorylation of ethanolamine **9**. A – ^{31}P NMR Spectrum of a reaction after 6.5 h carried out according to Procedure 9 using **9** as the nucleophile; B – Same sample as in spectrum A but this NMR spectrum was acquired with ^{1}H-^{31}P coupling (peak at 0.34 ppm splits into a triplet, indicating coupling to a CH_2 group); C – As spectrum A, spiked with a commercial sample of *O*-phosphorylethanolamine **30**.

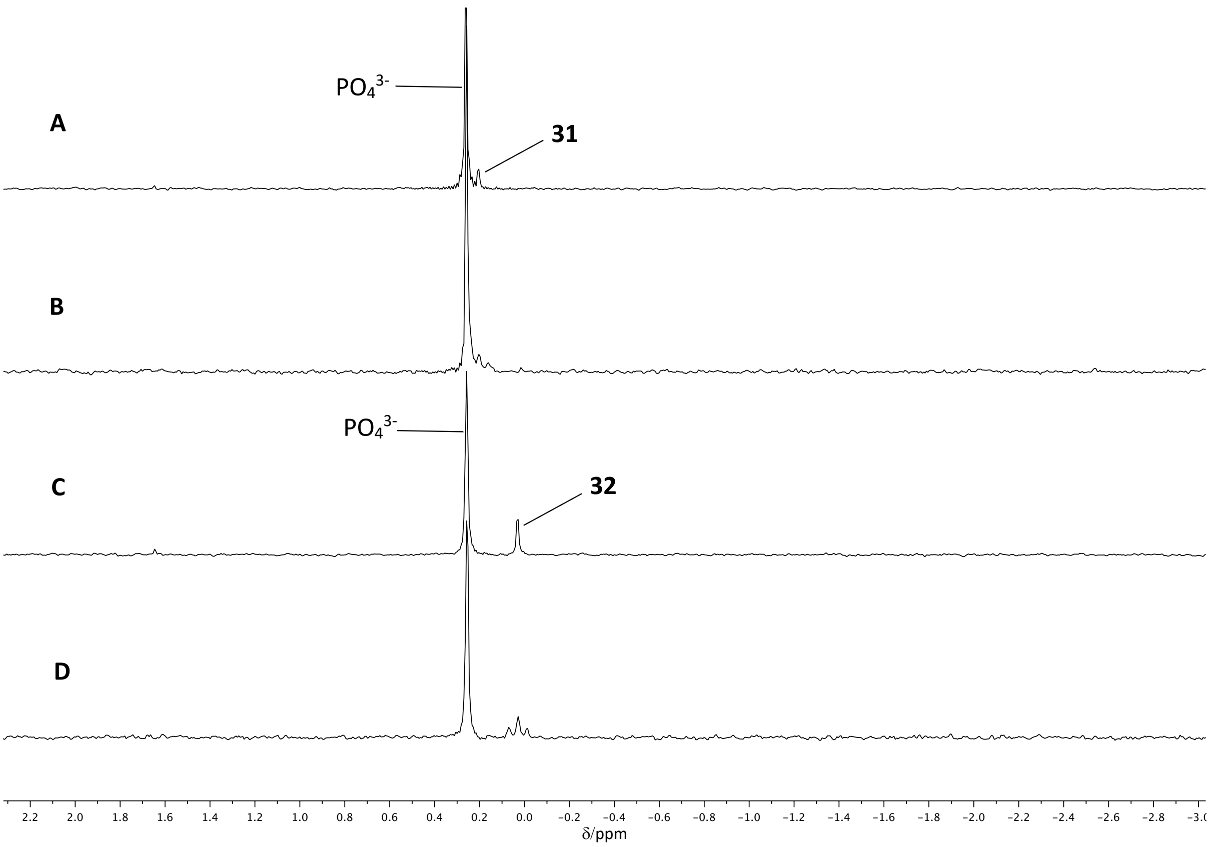

Supplementary Fig. 47 Synthesis of *N*-methylethanolamine phosphate **31** and *N,N*-dimethylethanolamine phosphate **32** from *N*-methyl ethanolamine **28** and *N,N*-dimethyl ethanolamine **29**, respectively. A – ^{31}P NMR Spectrum of a reaction after 6.5 h carried out according to Procedure 9 using **28** as the nucleophile; B – Same sample as in spectrum A, NMR spectrum acquired with ^{1}H-^{31}P coupling (peak at 0.20 ppm splits into a triplet, indicating coupling to a CH_2 group); C – ^{31}P NMR Spectrum of a reaction after 6.5 h carried out according to Procedure 9 using **29** as the nucleophile; D – Same sample as in spectrum C, NMR spectrum acquired with ^{1}H-^{31}P coupling (peak at 0.03 ppm splits into a triplet, indicating coupling to a CH_2 group).

Procedure 10

Glycolonitrile **1** (0.030 mmol, 3.0 μL) and $Na_3PSO_3.xH_2O$ (purity and water content predetermined and accounted for, 0.120 mmol) were dissolved in an Eppendorf tube with degassed 10% D_2O in H_2O (1 mL), then the pH was adjusted to 6.5 using degassed HCl. The volume was made up to 1.5 mL with degassed 10% D_2O in H_2O, the reaction was sealed and heated to 65 °C for 20 h and a ^1H NMR spectrum was acquired (Supplementary Fig. 48, spectrum A).

The NMR spectrum sample was recombined with the mother liquors and $Na_3PSO_3.xH_2O$ (purity and water content predetermined and accounted for, 0.120 mmol) was added. The pH was re-adjusted to 6.5 and the solution was sealed and transferred to a quartz cuvette. The reaction was irradiated for the desired amount of time after which it was analysed by ^1H NMR spectroscopy.

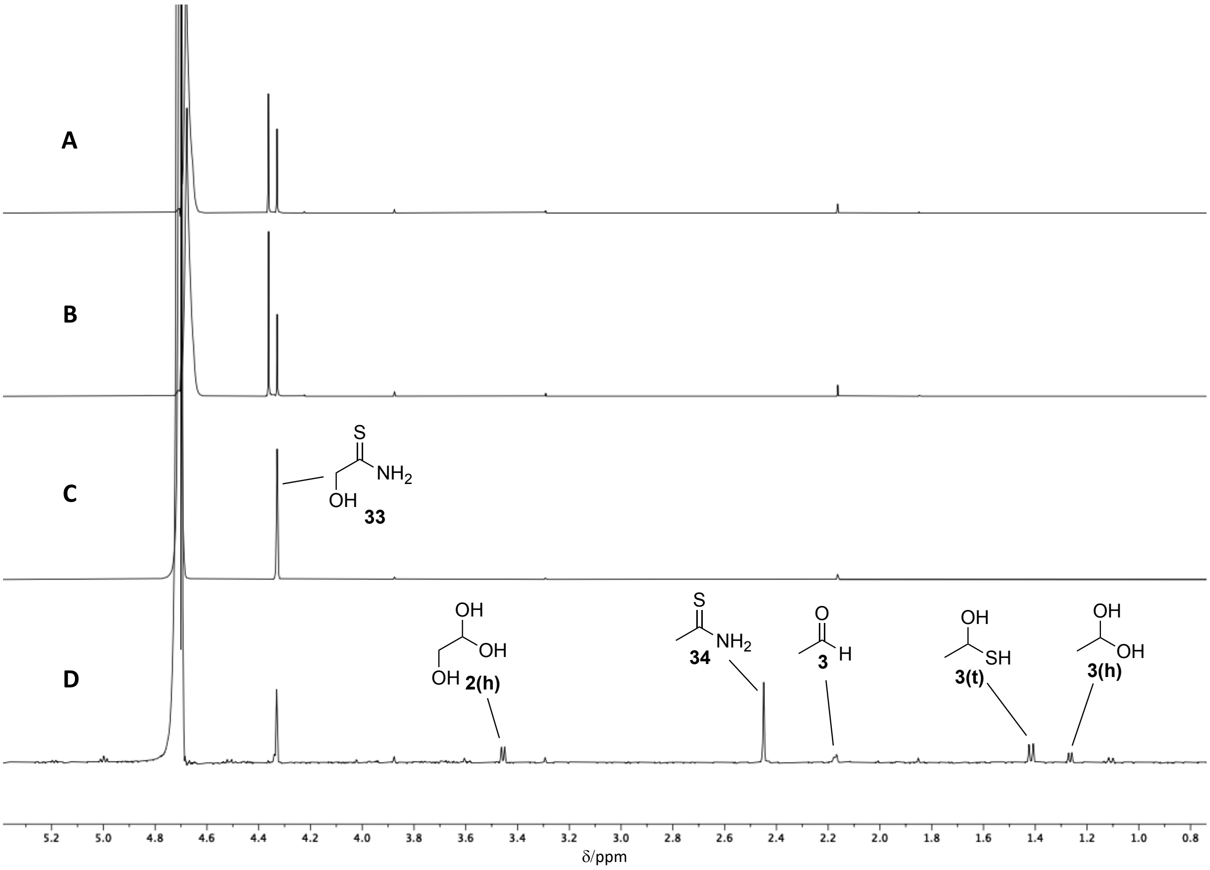

Supplementary Fig. 48 A – ^1H NMR Spectrum of a reaction carried out according to Procedure 10 after 0.5 h heating. A small aliquot was removed and this spectrum acquired; B – As spectrum A, spiked with a commercial sample of glycolonitrile **1**; C – ^1H NMR Spectrum of a reaction carried out according to Procedure 10 after 20 h of heating; D – ^1H NMR Spectrum of the reaction carried out according to Procedure 10 after 4 h irradiation. **3(t)** Designates the what we believe to be the thiohydrate of acetaldehyde **3**. Yield of **3** (~ 17%) includes **3(t)** and **3(h)**.

Procedure 11

Potassium cyanide (6.5 mg, 0.100 mmol), acetone **17** (3.7 μL, 0.050 mmol) and $Na_3PSO_3.xH_2O$ (purity and water content predetermined and accounted for, 0.250 mmol) were dissolved in an Eppendorf tube with degassed 10% D_2O in H_2O (0.6 mL), then the pH was adjusted to 6.5 using degassed HCl. The volume was made up to 1 mL with degassed 10% D_2O in H_2O, the reaction was sealed and heated to 50 °C for 4 h. A ^1H NMR spectrum of the crude reaction was collected (Supplementary Fig. 49, spectrum A). The pH was adjusted to 6.5 and the solution sparged with N_2 for 2 h before a ^1H NMR spectrum was collected (Supplementary Fig. 49, spectrum B). The volume was re-adjusted to 1 mL with degassed 10% D_2O in H_2O and transferred to a quartz cuvette, then the reaction was irradiated for 4 h. A ^1H NMR spectrum was collected (Supplementary Fig. 49, spectrum C) then KCN (2 mg, 0.031 mmol) was added to the NMR sample and the pH adjusted to 6.5, after which a further ^1H NMR spectrum was collected (Supplementary Fig. 49, spectrum D).

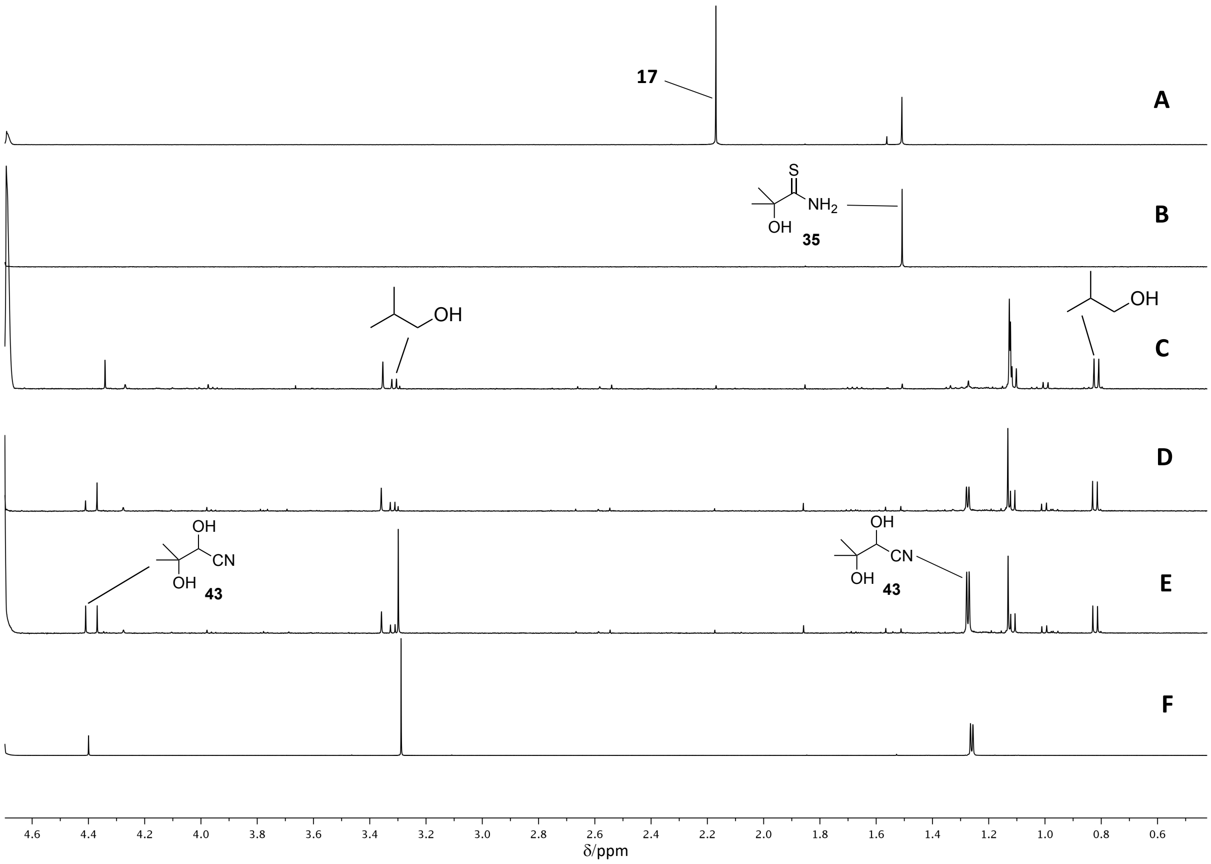

Supplementary Fig. 49 Synthesis of cyanohydrin **43**. A – ^1H NMR Spectrum of a reaction carried out according to Procedure 11 after 4 h of heating acetone cyanohydrin with PSO_3^{3-} (singlet at 2.17 ppm is due to residual acetone **17**); B – As spectrum A after sparging with N_2;

C – As spectrum B after irradiation for 4 h; D – As spectrum C after addition of HCN; E – As spectrum D after spiking with synthetically prepared **43** (singlet at 3.29 ppm due to residual MeOH from the preparation of **43**, see Synthetic procedures to make standards (p. 81) and Supplementary Fig. 67); F – ^{1}H NMR Spectrum of synthetically prepared **43** (singlet at 3.29 ppm due to residual MeOH from the preparation of **43**, see Synthetic procedures to make standards (p. 81) and Supplementary Fig. 67).

Procedure 12

Potassium cyanide (14 mg, 0.220 mmol) was dissolved in a solution of α–hydroxyisobutyraldehyde **38** (2 mL, *ca.* 100 mM, as prepared in Synthetic procedures to make standards, p. 75) and $Na_3PSO_3.xH_2O$ (purity and water content predetermined and accounted for, 1.2 mmol was added). The pH was adjusted to 6.5 using degassed HCl (final volume *ca.* 2.2 mL) and the reaction was sealed and heated to 50 °C for 2 days at which point a ^1H NMR spectrum was collected (Supplementary Fig. 50, spectrum B). Of this reaction mixture, a portion was taken (0.5 mL) and dissolved in degassed 10% D_2O in H_2O (1 mL) and $Na_3PSO_3.xH_2O$ (purity and water content predetermined and accounted for, 0.200 mmol) was added. The pH was adjusted to 6.5 then the volume made up to 2 mL with degassed 10% D_2O in H_2O. The resulting solution was transferred to a quartz cuvette, sealed and irradiated for 12 h.

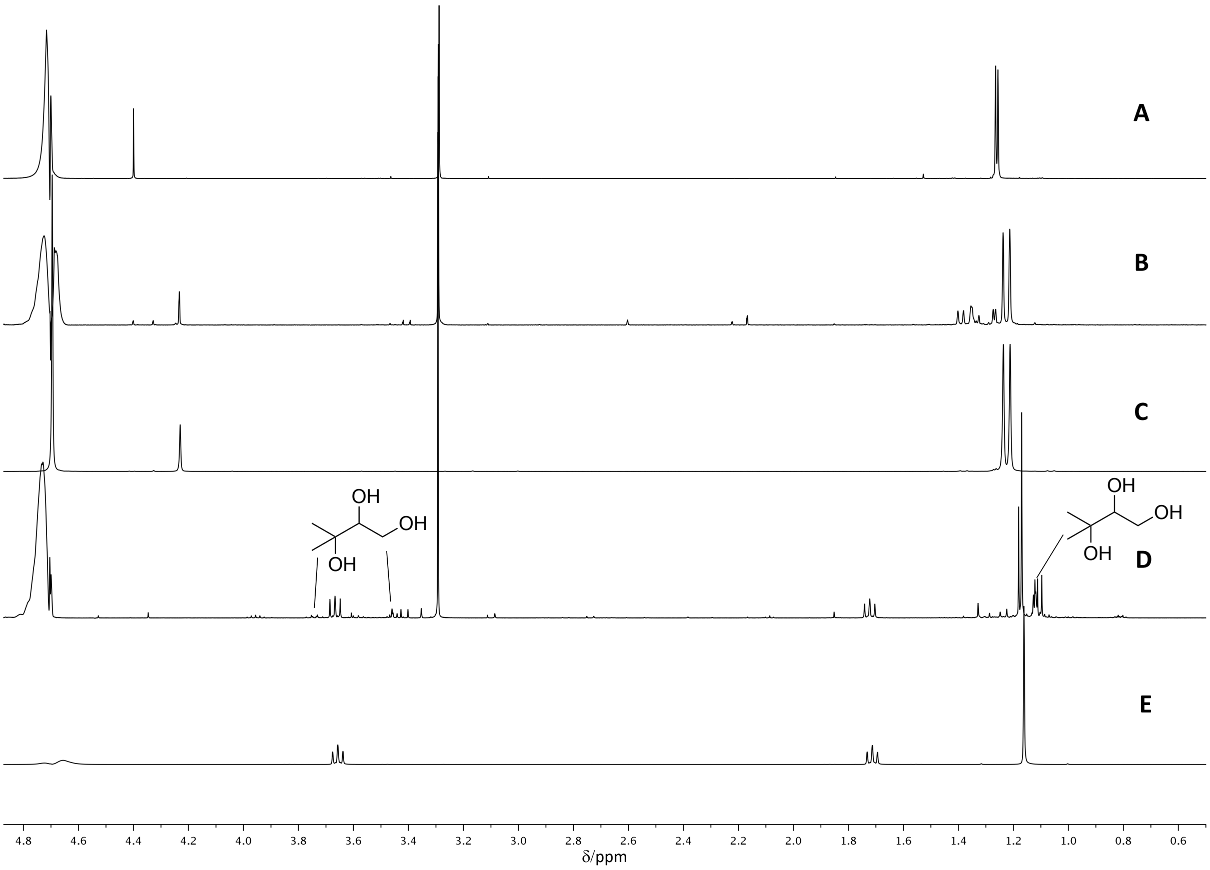

Supplementary Fig. 50 Synthesis of 3-methyl-butane-1,3-diol **39**. A – ^1H NMR Spectrum of the cyanohydrin formed after addition of HCN to α–hydroxyisobutyraldehyde **38** (peak at 3.33 ppm due to MeOH present in the initial aldehyde **38** solution, see Synthetic procedures to make standards, p. 81 and Supplementary Figs. 65 and 67); B – ^1H NMR Spectrum of a reaction carried out according to Procedure 12 after heating for 2 days (peak at 3.33 ppm due to MeOH

present in the initial aldehyde **38** solution, see Supplementary Figs. 65 and 67); C – ^{1}H NMR Spectrum of a synthetic standard of thioamide **44** (See Synthetic procedures to make standards, p.81 and Supplementary Fig. 69); D – As spectrum B, after further addition of Na_3PSO_3 and irradiation for 12 h, according to Procedure 12 (peak at 3.33 ppm due to MeOH present in the initial aldehyde **38** solution, see Supplementary Figs. 65 and 67); E – ^{1}H NMR Spectrum of a commercial sample of 3-methylbutane-1,3-diol **39**.

Supplemententry Table 11 Thiolysis of cyanohydrin **43** and reduction of the resulting thioamide, **44**

Entry	Spectrum	Starting material	PSO_3^{3-}	Conditions	Product and yield
1	Supp. Fig. 50, Spectrum B	**43** (~ 90 mM)	545 mM	50 °C, 2 d	Thioamide **44**, ~ 64%
2	Supp. Fig. 50, not shown	**43** (~ 90 mM)	200 mM	50 °C, 3 d	Thioamide **44**, ~ 41%
3	Supp. Fig. 50, Spectrum D	**44** (~ 14 mM)	100 mM	UV, 12 h	3-Methyl-1,3-butanediol **39**, ~ 62%<br><br>3-Methyl-1,2,3-butanetriol, ~ 16%

Procedure 13

3-Methyl-butane-1,3-diol **39** (10 mg, 0.100 mmol) was dissolved in formamide (1 mL) and $(NH_4)H_2PO_4$ (35 mg, 0.300 mmol) was added. The tube was sealed, stirred and heated to the desired temperature. At the appropriate time point, a small portion of the reaction was diluted with D_2O and a ^1H NMR spectrum was acquired.

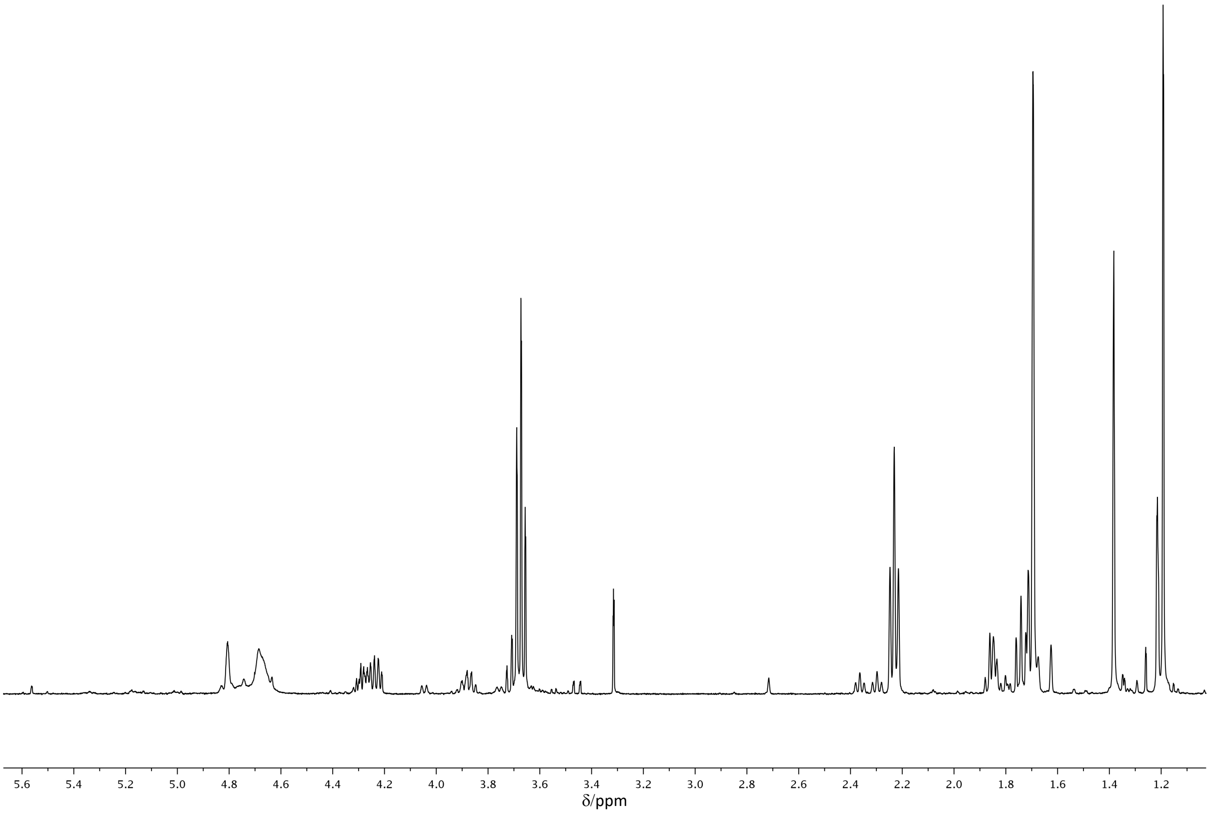

Supplementary Fig. 51 Prebiotic synthesis of potential isoprenoid precursors **42**, **45** and **46** (see Supplementary Fig. 52 for identification). ^1H NMR Spectrum of a reaction after 22 h at 150 °C according to Procedure 13.

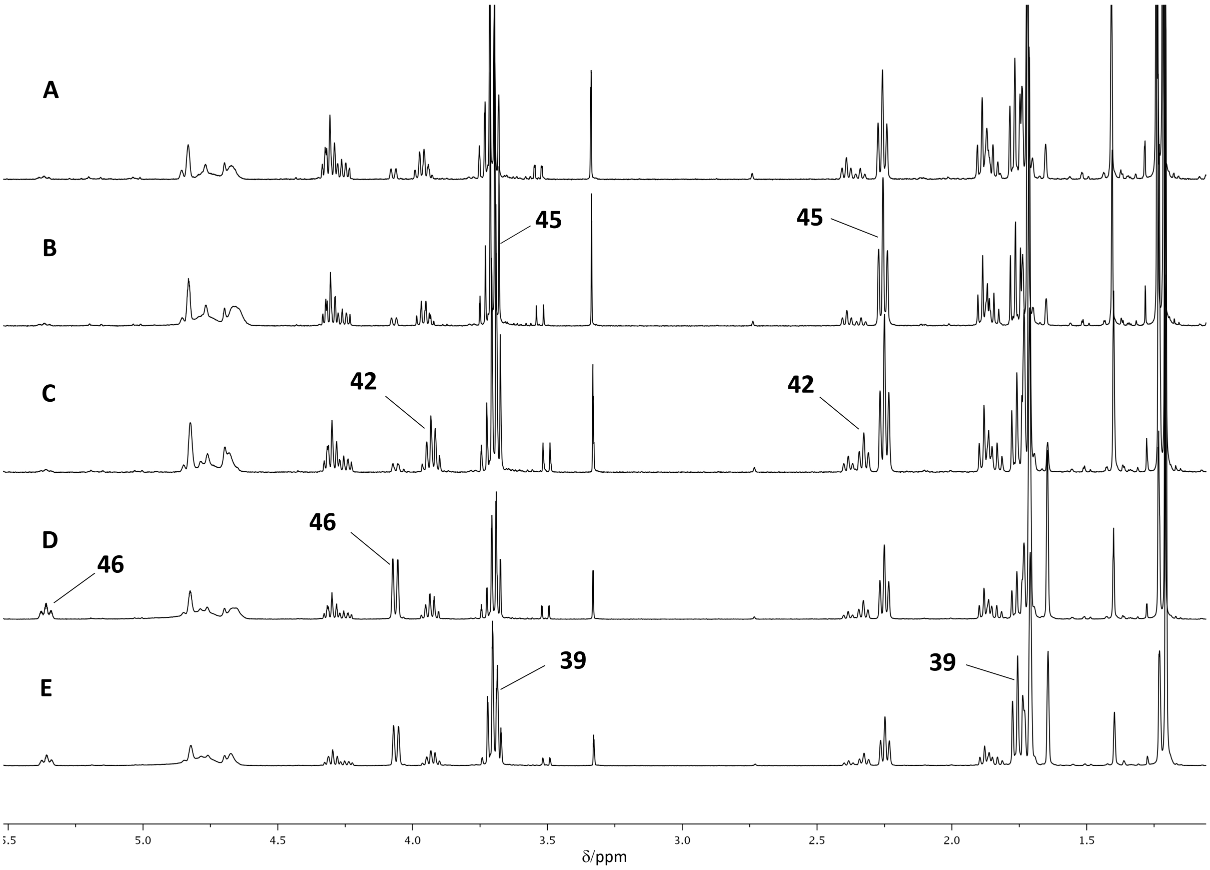

Supplementary Fig. 52 Identification of potential isoprenoid precursors **42**, **45** and **46**. A – ^{1}H NMR Spectrum of a reaction carried out according to Procedure 13 after heating for 8 h at 150 °C; B – As spectrum A, spiked with a commercial sample of isopentenyl alcohol **45**; C – As spectrum B, spiked with a synthetic sample of isopentenyl phosphate **42** (See Synthetic procedures to make standards, p. 82 and Supplementary Fig. 73); D – As spectrum C, spiked with a commercial sample of dimethylallyl alcohol **46**; E – As spectrum D, spiked with a commercial sample of 3-methyl-butane-1,3-diol **39**.

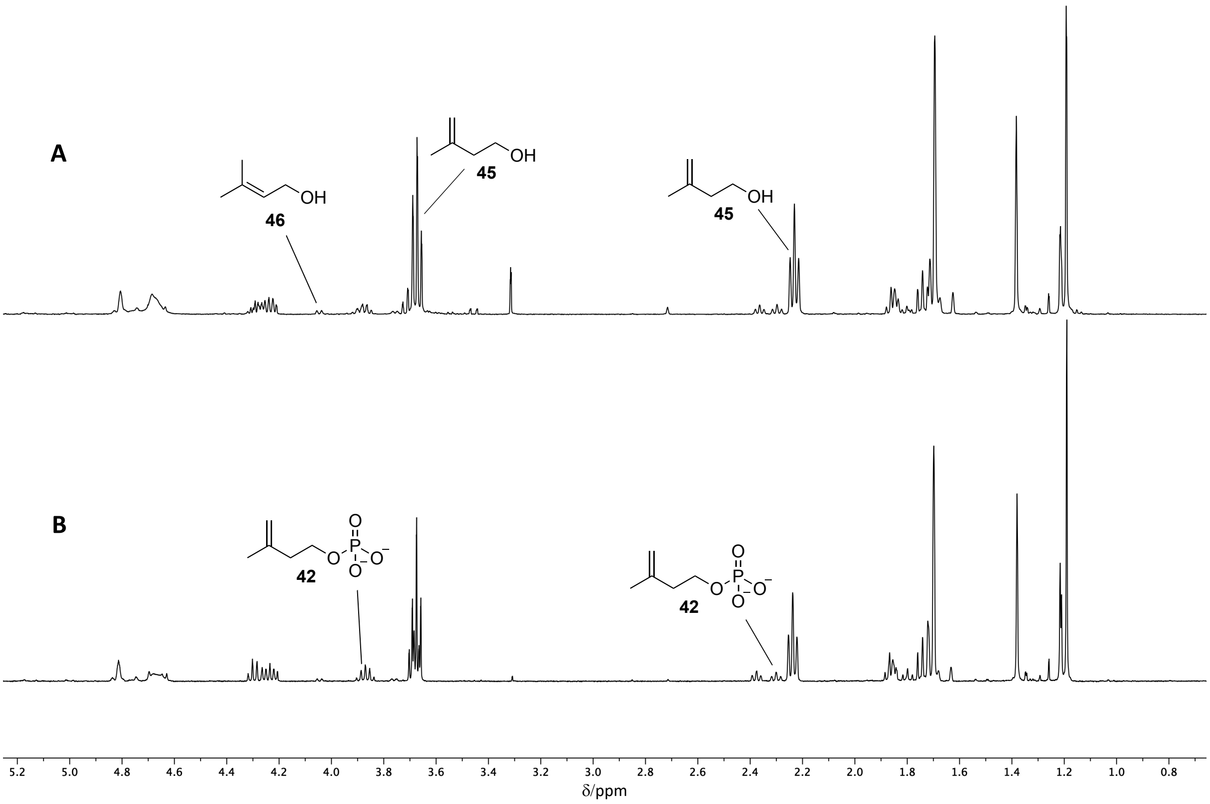

Supplementary Fig. 53 Comparison of elimination reactions of **39** at different temperatures. A – ^{1}H NMR Spectrum of a reaction carried out according to Procedure 13 after heating for 8 h at 150 °C; B – ^{1}H NMR Spectrum of a reaction carried out according to Procedure 13 after heating for 5 d at 120 °C.

Supplementary Table 12 Comparison of elimination reactions of **39** at different temperatures

Entry	Spectrum	**39**	Temp.	Time	Yield of products		
					**42**	**45**	**46**
1	Supp. Fig. 53, Spectrum A	100 mM	150 °C	22 h	~6%	~48%	~3%
2	Supp. Fig. 53, Spectrum B	100 mM	120 °C	5 d	~6%	~53%	~2%

42 - Isopentenyl phosphate
45 - Isopentenyl alcohol
46 - Dimethylallyl alcohol

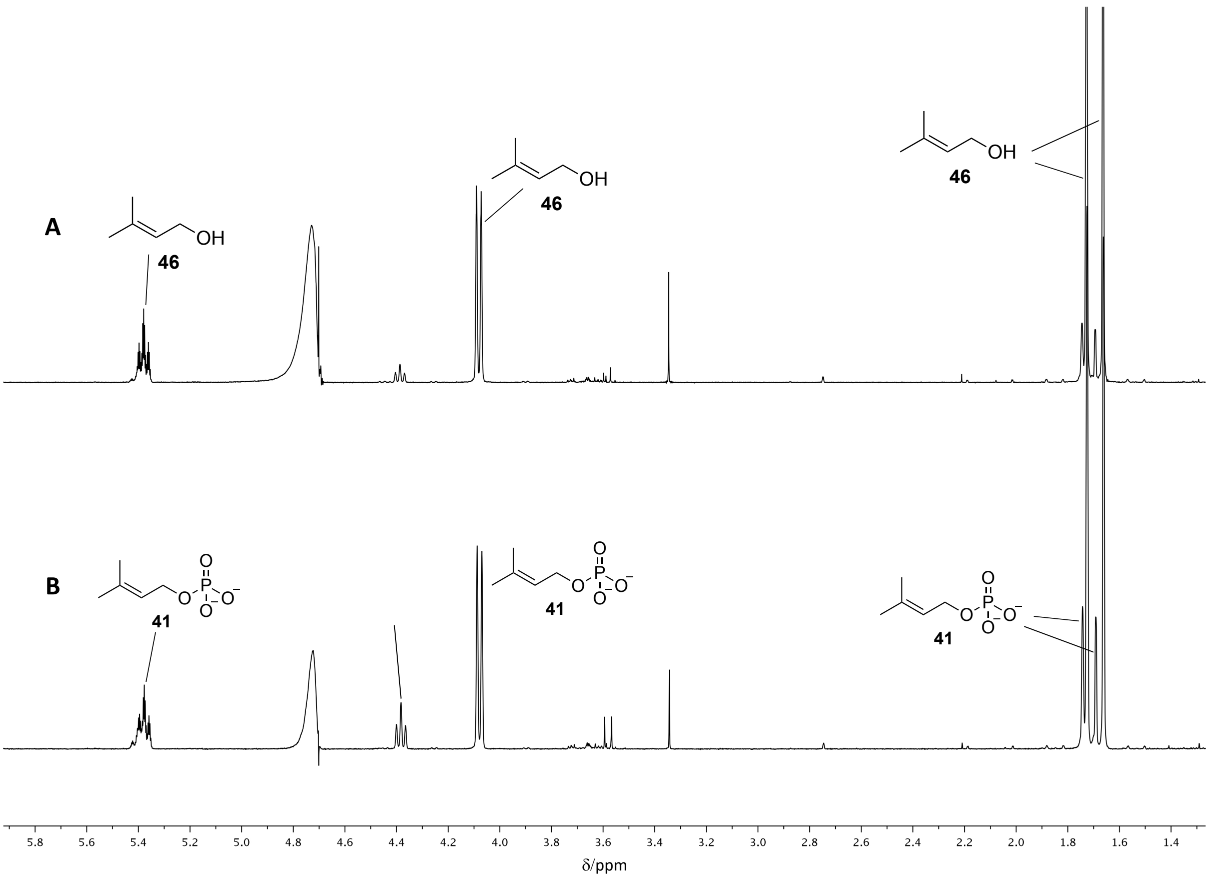

Supplementary Fig. 54 Phosphorylation of dimethylallyl alcohol **46**. Dibasic thiophosphate was prepared according to General procedure 1 (0.050 mmol or 0.100 mmol). Dimethylallyl alcohol **46** (5.1 μL, 0.050 mmol) was dissolved in degassed formamide (1 mL) and added to the thiophosphate. Potassium ferricyanide (66 mg, 0.200 mmol) was then added and the reaction agitated for the desired time before a sample was removed, diluted with D_2O and examined by ^{31}P NMR spectroscopy. A – ^{31}P NMR Spectrum of a reaction after 7 h reaction; B – As spectrum A spiked with a sample of dimethylallyl phosphate **41** (see Synthetic procedures to make standards, p.82 and Supplementary Fig. 71).

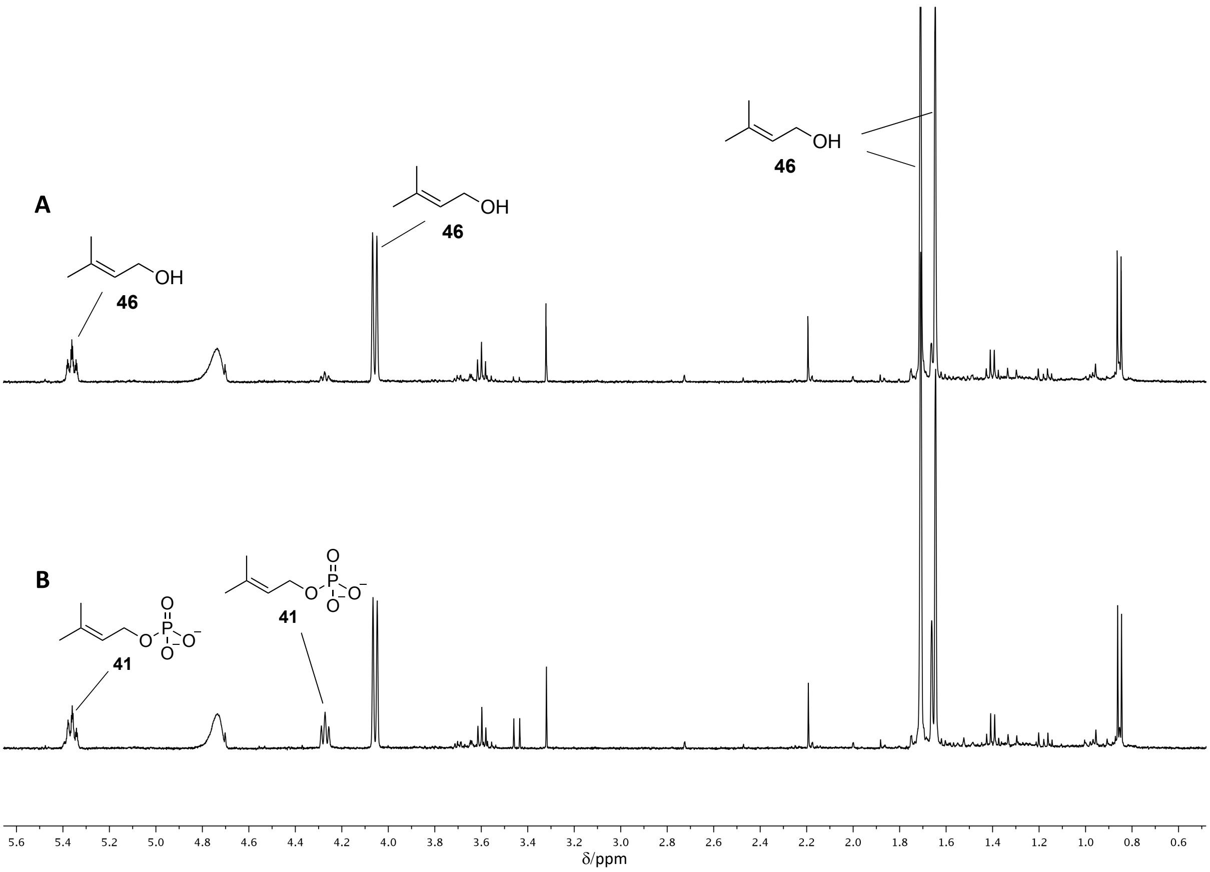

Supplementary Fig. 55 Phosphorylation of dimethylallyl alcohol **46**. Dibasic thiophosphate was prepared according to General procedure 1 (0.100 mmol). Dimethylallyl alcohol **46** (5.1 μL, 0.050 mmol) was dissolved in degassed formamide (1 mL), added to the thiophosphate and then the solution was transferred to a quartz cuvette. The reaction was sealed and irradiated for the desired amount of time before a sample was removed, diluted with D_2O and examined by ^{31}P NMR spectroscopy. A – ^{31}P NMR Spectrum of a reaction after 5 h; B – As spectrum A spiked with a sample of **41** (see Synthetic procedures to make standards, p.82 and Supplementary Fig. 71).

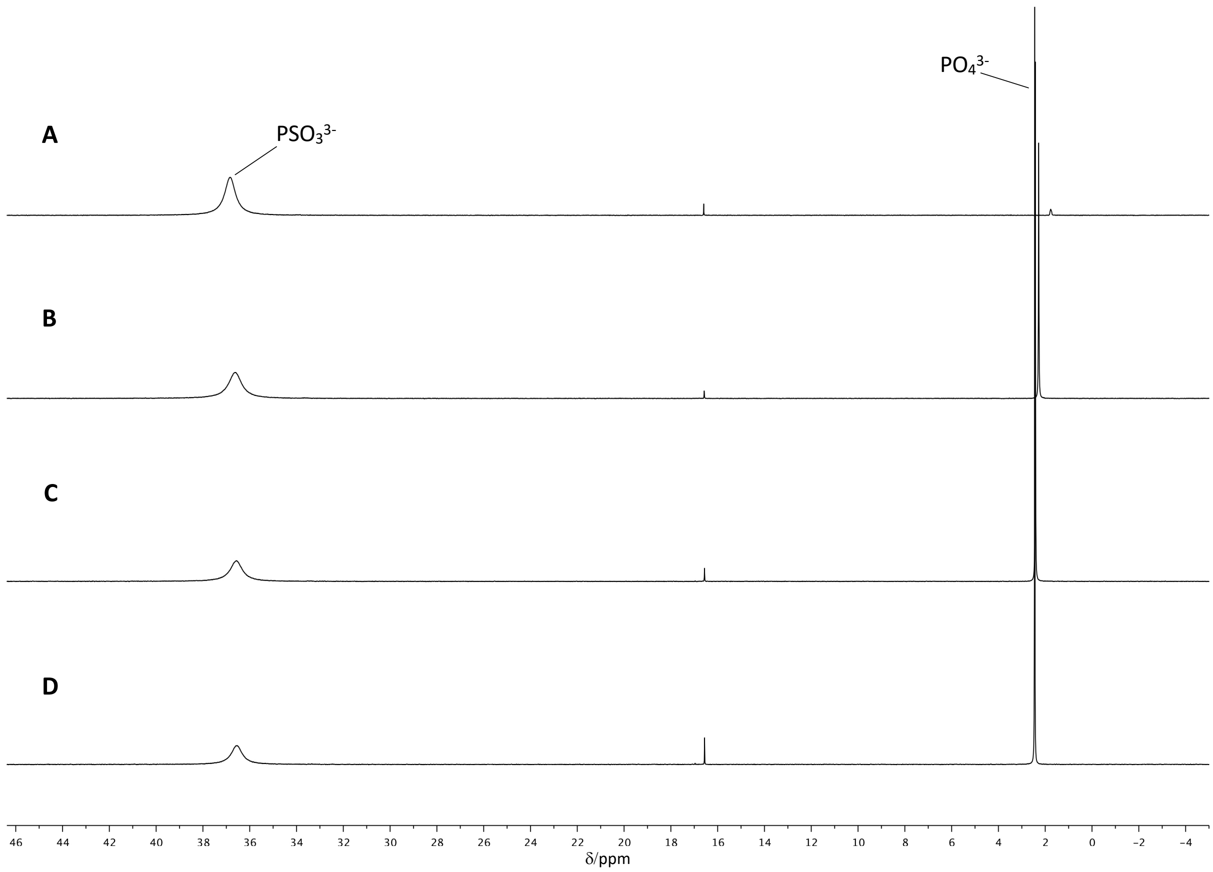

Supplementary Fig. 56 ^{31}P NMR Spectra of a time course for the hydrolysis of $Na_3PSO_3.xH_2O$ (50 mM) in degassed 10% D_2O in H_2O at pH 6.5 and ambient temperature. A – t = 0, 99% thiophosphate remains; B – t = 7 days, 75% thiophosphate remains; C – t = 13 days, 57% thiophosphate remains; D – t = 19 days, 45% thiophosphate remains. The rate of hydrolysis is actually much slower than the reported rate,[49] but this is likely due to the use of a molybdate complex used to quantify orthophosphate production which can undergo reaction with sulfides to form sulfido complexes.[50] The reagent can also be reduced by sulfides giving erroneous measurements.[50]

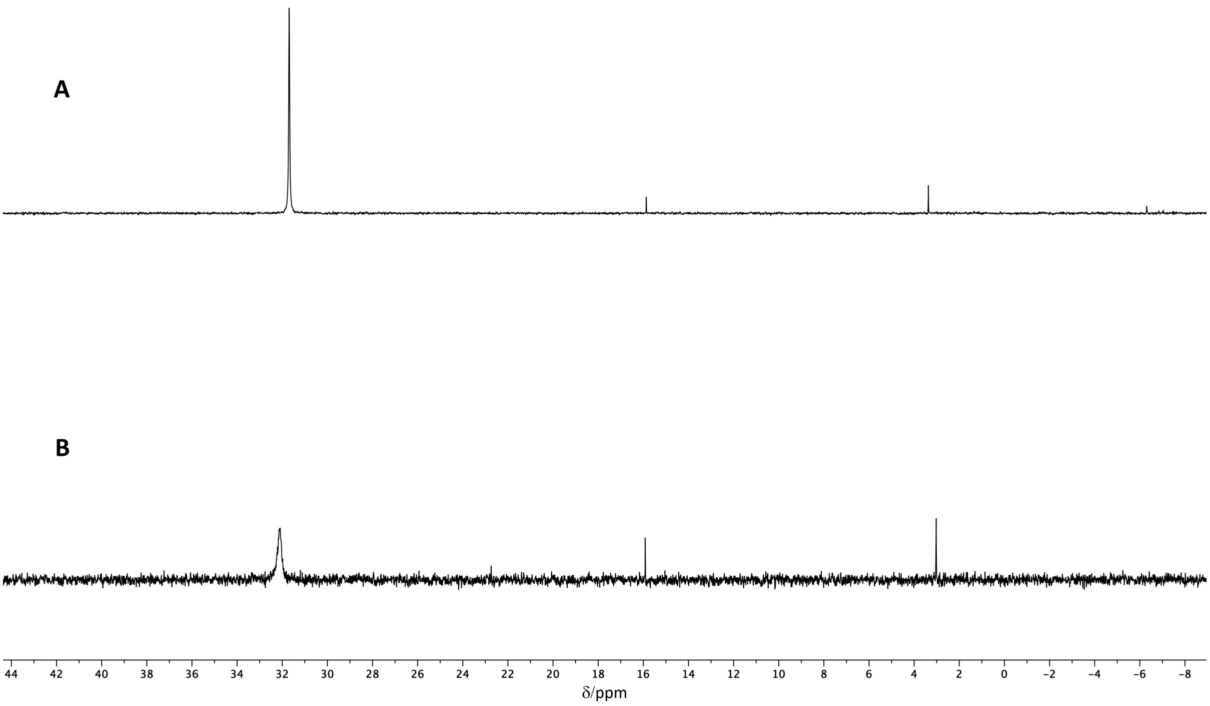

Supplementary Fig. 57 ^{31}P NMR Spectra of a time course for the hydrolysis of $Na_3PSO_3.xH_2O$ (50 mM) in degassed 10% D_2O in H_2O at pH 12 and ambient temperature. A – t = 0, 94% thiophosphate remains; B – t = 6 weeks, 89% thiophosphate remains.

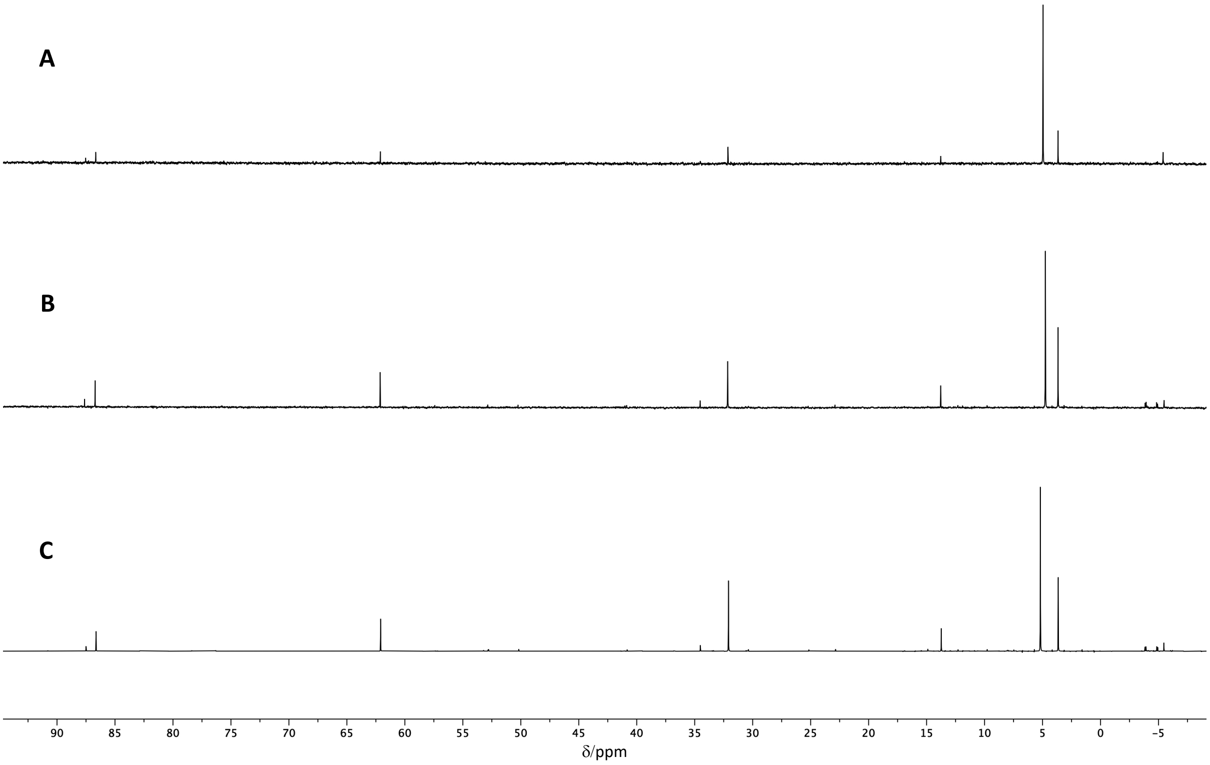

Supplementary Fig. 58 Corrosion experiment of Fe_3P (a commercial surrogate for schreibersite[51]) by water containing HS^-/H_2S. $NaSH.xH_2O$ (25 mg, 0.267 mmol) was dissolved in degassed 10% D_2O in H_2O (1 mL) in an Eppendorf and Fe_3P (300 mg, 0.151 mmol) was added. The suspension was sealed and gently agitated after the desired amount of time a sample was removed, centrifuged and examined by ^{31}P NMR spectroscopy. A – Quantitative ^{31}P NMR spectrum of the reaction after 3 d; B – Quantitative ^{31}P NMR spectrum of the reaction after 6 d; C – Quantitative ^{31}P NMR spectrum of the reaction after 12 d. The singlet at 32.1 ppm corresponds to thiophosphate (spike not shown). The percentage of the total of the integrals of the signals in the ^{31}P NMR spectra above that accounts for thiophosphate was as follows: 6% in spectrum A, 13% in spectrum B and 15% in spectrum C. For discussion of some of the other species present, see Supplementary Fig. 59. Note: the endpoint for accumulation of thiophosphate was not measured.

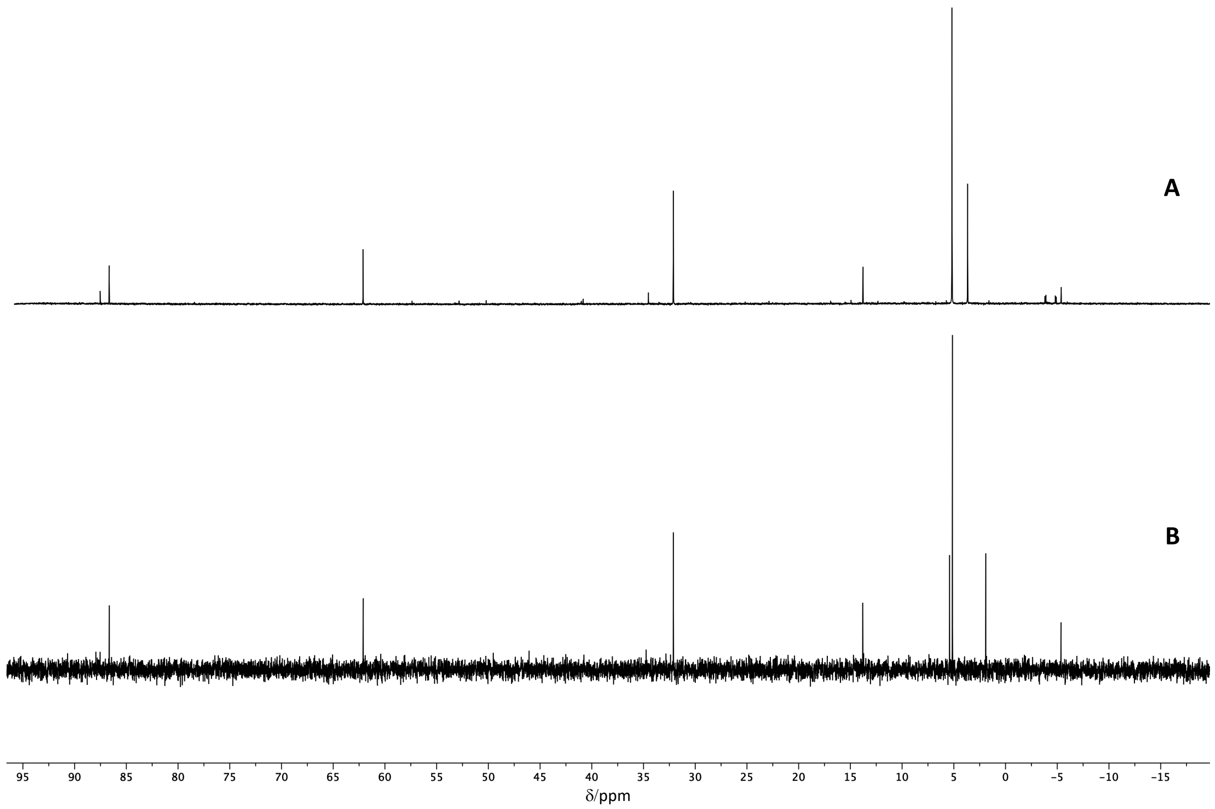

Supplementary Fig. 59 $NaSH.xH_2O$ (25 mg, 0.267 mmol) was dissolved in degassed 10% D_2O in H_2O (1 mL) in an Eppendorf and Fe_3P (300 mg, 0.151 mmol) was added. The suspension was sealed and gently agitated for 6 d, centrifuged and the supernatant examined by ^{31}P NMR spectroscopy, which gave spectrum A. B – As spectrum A, but the NMR spectrum was acquired with ^{31}P-^{1}H coupling. In spectrum A, the singlet at - 5.48 ppm is due to pyrophosphate; the doublet of doublets (- 3.89 (J = 17.1), - 4.88 (J = 17.1)) is tentatively assigned to the phosphite-phosphate anhydride (isohypophosphate) given the ^{31}P signals disappear upon coupling to H nuclei. Although the literature values for the chemical shifts differ (- 4.4 and - 9.6 ppm), those studies were conducted at acidic pH[52]); the singlet at 3.66 ppm corresponds to phosphite; singlet at 4.76 ppm corresponds to orthophosphate; the singlet at 32.1 ppm corresponds to thiophosphate; whilst the other major signals could not be definitively assigned, it is worth noting that the phosphorus atoms are not attached to hydrogen – although the signal at 87.6 ppm appears to be split, it actually aligns with the right-hand, apparent doublet in spectrum B. We also note that species of the type dithiphosphate ($PO_2S_2^{3-}$) and trithiophosphate (POS_3^{3-}) can be observed in the range ~ 60 – 90 ppm and 85 – 101 ppm, respectively, depending on pH.[53]

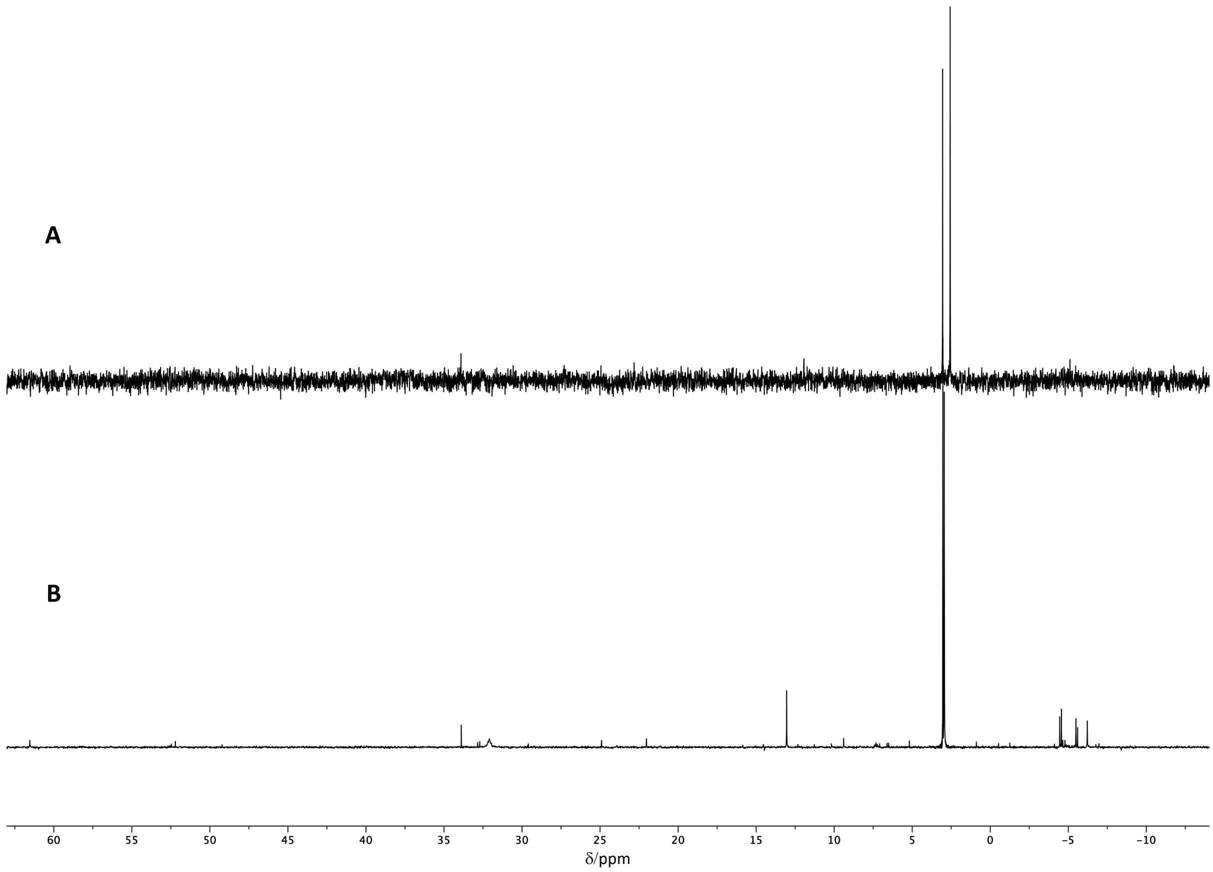

Supplementary Fig. 60 Corrosion experiment of Fe_3P (a commercial surrogate for schreibersite[51]) by water containing HS^-/H_2S at neutral pH. $NaSH.xH_2O$ (12 mg, 0.129 mmol) was dissolved in degassed 10% D_2O in H_2O (0.6 mL) in an Eppendorf and the pH was adjusted to 7.2 with degassed HCl. Fe_3P (300 mg, 0.151 mmol) was added and the suspension was sealed and gently agitated for the desired amount of time. The suspension was briefly centrifuged and the pH checked, then a sample was removed and examined by ^{31}P NMR spectroscopy. A – ^{31}P NMR spectrum of the supernatant after 7 d, the pH was ~ 8; B – ^{31}P NMR spectrum of the supernatant after 4 weeks, the pH was ~ 11.4. In spectrum B, the broad signal at 32 ppm corresponds to thiophosphate, which integrates for ~ 10% of the all the signals in the ^{31}P NMR spectrum.

Procedure 14

$NaH_2PO_4.2H_2O$ (if used, 2 mg, 0.013 mmol), KCN (6 mg, 0.090 mmol), $NaSH.xH_2O$ (> 60%, 8 mg, 0.090 mmol), $HPO_3Na_2.5H_2O$ (if used, 9 mg, 0.040 mmol) and hypophosphorous acid (if used, 50% wt. 8.3 μL, 0.080 mmol) were dissolved in degassed 10% D_2O in H_2O (1 mL) and the pH adjusted to 6.5. Glycolonitrile **1** (55% wt. 3.0 μL, 0.030 mmol) was added, the volume was made up to 2 mL with degassed 10% D_2O in H_2O before the solution was transferred to a cuvette and irradiated.

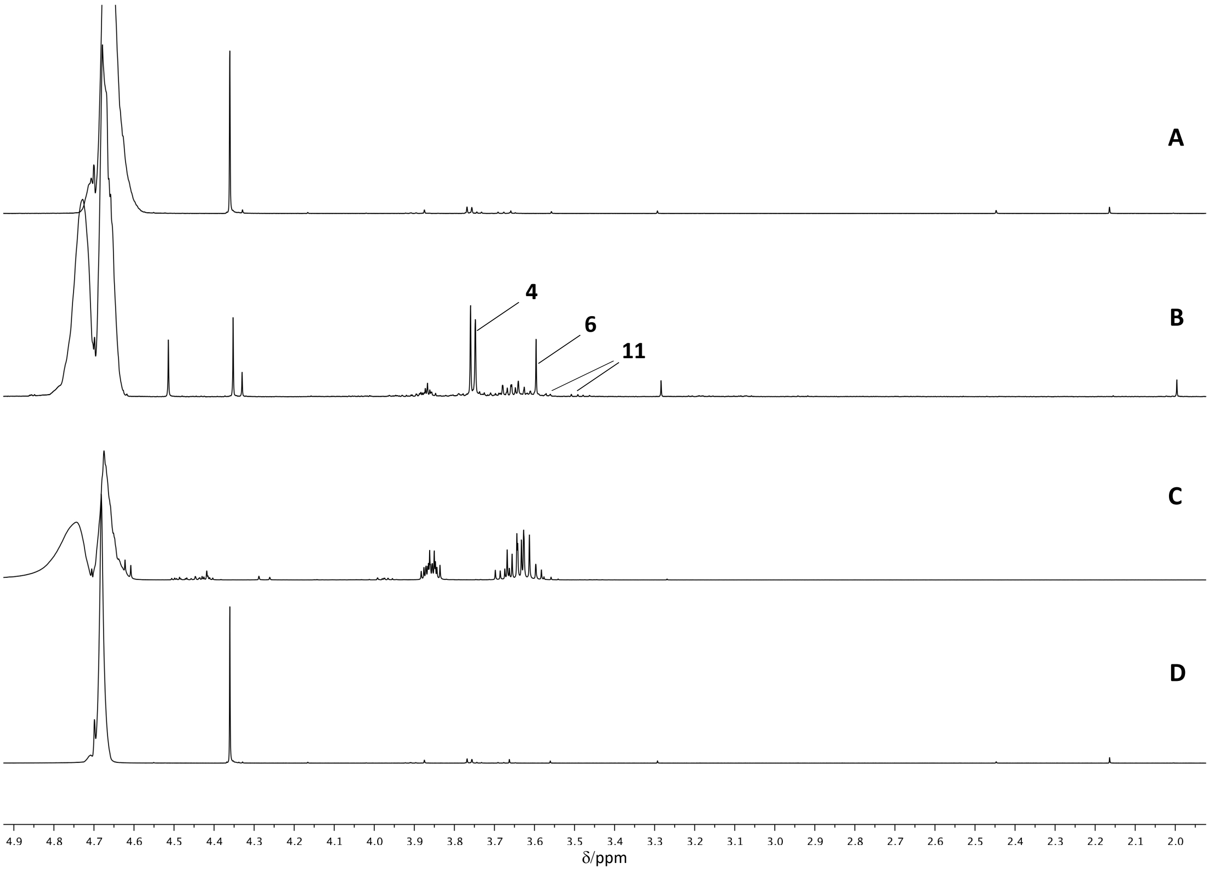

Supplementary Fig. 61 The photochemical reduction of nitriles by HS^- in the presence and absence of hypophosphite, phosphite and phosphate. A – ^1H NMR Spectrum of the crude reaction mixture after 1 h according to Procedure 14 when no phosphorus species were included in the reaction, ~ 4% of glyceronitrile **4** was present; B – ^1H NMR Spectrum of the crude reaction mixture after 1 h according to Procedure 14 with the inclusion of phosphorus species, ~ 26% of glyceronitrile **4**, ~ 16% of glyceraldehyde cyanohydrin **10**, ~ 3% ethylene glycol **6** and ~ 3% glycerol **11** were present; C – ^1H NMR Spectrum of glyceraldehyde cyanohydrin **10**; D – As spectrum A with the inclusion of $NaH_2PO_4.2H_2O$. As can be seen by comparing spectra A and D, the rate enhancement observed in spectrum B must be due to the inclusion of the reduced phosphorus species. The ratio of HPO_3^{2-}:$H_2PO_2^-$:PO_4^{3-} is the same

as that reported by Bryant and Kee for the anoxic corrosion of schreibersite under conditions of UV irradiation, and thus seem most relevant to our geochemical scenario.[13]

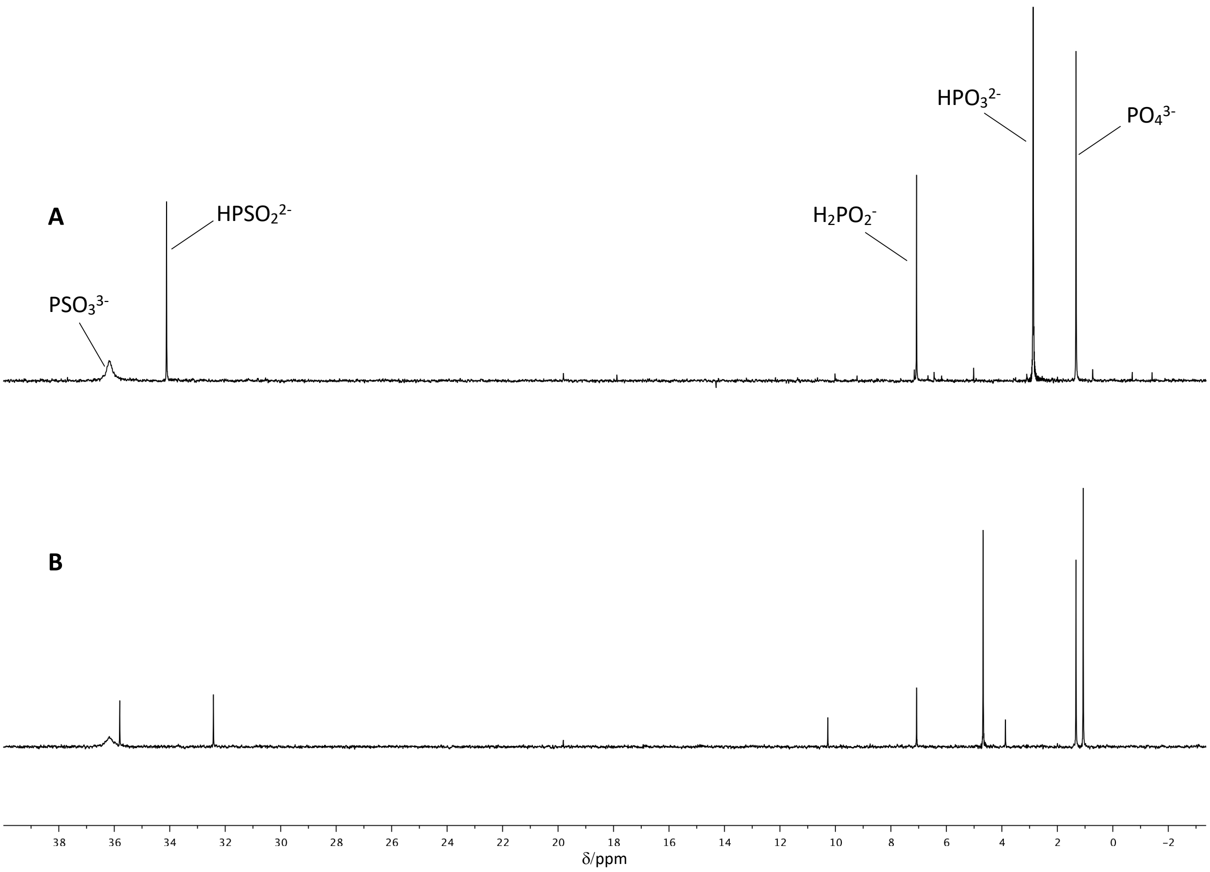

Supplementary Fig. 62 The photochemical reduction of nitriles by HS^- in the presence of hypophosphite, phosphite and phosphate. A – ^{31}P NMR Spectrum of the same sample as Supplementary Fig. 61, spectrum B; B – As spectrum A, but the NMR spectrum was acquired with ^{31}P-^{1}H coupling. The presence of thiophosphate (PSO_3^{3-}) and thiophosphite ($HPSO_2^{2-}$) is clear, and as these compounds cannot be formed in the above experiments where the rate of reduction of glycolonitrile is slow (Supplementary Fig. 61, spectra A and D) it is presumably their rapid formation and photolysis which provides the observed rate enhancement.

Synthetic procedures to make standards

α–Hydroxyisobutyraldehyde 38[54]

The synthesis follows that outlined in Ref. 54. Methyl glyoxal 1,1-dimethyl acetal (3.50 g, 3.6 mL, 25.4 mmol) was dissolved in anhydrous Et_2O (30 mL) under Ar and cooled to 0 °C. MeMgBr (3.0 M in Et_2O, 10.6 mL, 31.8 mmol) was added dropwise over 10 min then the reaction was warmed to room temperature and stirred 3 h. A saturated solution to of NH_4Cl (18 mL) was carefully added and the layers separated. The aqueous layer was extracted with Et_2O (2 x 25 mL), the organic layers combined, dried ($MgSO_4$), filtered and carefully concentrated (38 °C, 220 mbar). The residue was purified by flash chromatography (pentane/Et_2O, 3:2 – 1:1) and concentrated carefully *in vacuo* to give 2-hydroxy-2-methylpropanal 1,1-dimethyl acetal as a pale-yellow oil (1.34 g, 9.99 mmol, 39% (further extraction may have been necessary as product was still in the aqueous layer as observed by ^{1}H NMR spectroscopy)). R_F 0.18 (hexane/Et_2O, 1:1); ^{1}H NMR (D_2O) δ 4.10 (s, 1H), 3.51 (s, 6H), 1.10 (s, 6H); ^{13}C NMR (D_2O) δ 111.4, 72.3, 58.3, 22.8. See Supplementary Figs. 63 and 64.

2-Hydroxy-2-methylpropanal 1,1-dimethyl acetal (100 mg, 0.746 mmol) was dissolved in H_2O (7.5 mL) and Dowex-50W X8(H^+) was added. The reaction was stirred for 4 days and filtered under vacuum to give a ~ 100 mM aqueous solution of α–hydroxyisobutyraldehyde **38**. ^{1}H NMR (D_2O) δ 1.12 (s, 6H); ^{13}C NMR (D_2O) δ 94.3, 73.0, 22.7. See Supplementary Figs. 65 and 66.

2-Hydroxy-2-methylpropanal cyanohydrin 43 and 2-hydroxy-3,3-hydroxymethylbutanethioamide 44

2-Hydroxy-2-methylpropanal 1,1-dimethyl acetal (as prepared above, 186 mg, 1.39 mmol) was dissolved in H_2O (10 mL) and Dowex-50W X8(H^+) was added. The reaction was stirred for 3 d then filtered under vacuum washing with H_2O (2 mL) and KCN (104 mg, 1.60 mmol) and $Na_3PSO_3.xH_2O$ (purity and quantity of H_2O predetermined and accounted for, 5.56 mmol) were added. The pH was adjusted to 6.5 and ^{1}H and ^{13}C NMR spectra of cyanohydrin **43** were collected. ^{1}H NMR (D_2O) δ 4.39 (s, 1H), 1.26 (s, 3H), 1.25 (s, 3H); ^{13}C NMR (D_2O) δ 119.7, 71.9, 68.9, 24.2, 23.4. See Supplementary Figs. 67 and 68.

The reaction was sealed and heated to 50 °C with stirring for 24 h. A second portion of $Na_3PSO_3.xH_2O$ was added (2.80 mmol) then the pH was re-adjusted to 6.5 and heating continued for a further 24 h. The product was extracted with EtOAc (4 x 15 mL) and the organics combined and dried with $MgSO_4$. The solution was filtered, concentrated *in vacuo* and the residue purified by flash chromatography (Et_2O/hexane, 4:1) to give thioamide **44** as colourless solid (98 mg, 0.657 mmol, 47%, 3 steps). R_F 0.21 (Et_2O/hexane, 4:1); ^{1}H NMR (D_2O) δ 4.23 (s, 1H), 1.24 (s, 3H), 1.21 (s, 3H); ^{13}C NMR (D_2O) δ 206.7, 82.3, 72.5, 25.3, 24.2; ESI-LCMS (+ve, m/z): 150 $[M+H]^+$. Supplementary Figs. 69 and 70.

Dimethylallyl phosphate 41

Dimethylallyl alcohol **46** (0.100 mmol, 10.1 μL) was dissolved in formamide (1 mL) then urea (1.00 mmol, 60 mg) and $(NH_4)H_2PO_4$ (0.100 mmol, 12 mg) were added. The Eppendorf tube was sealed and the reaction heated to 110 °C with stirring for 18 h. The reaction was then concentrated *in vacuo* at 88 °C to the point of dryness. ^{1}H NMR (D_2O) δ 5.37 (t, J = 7.5, 1H), 4.32 (t, J = 7.2, 2H), 1.71 (s, 3H), 1.66 (s, 3H); ^{31}P NMR (D_2O) δ 0.96. See Supplementary Figs. 71 and 72.

Isopentenyl phosphate 42

Isopentenyl alcohol **45** (0.100 mmol, 10.1 μL) was dissolved in formamide (1 mL) and urea (1.00 mmol, 60 mg) and $(NH_4)H_2PO_4$ (0.100 mmol, 12 mg) were added. The Eppendorf tube was sealed and the reaction heated to 110 °C with stirring for 18 h. The reaction was then concentrated *in vacuo* at 88 °C to the point of dryness. ^{1}H NMR (D_2O) δ 3.80 (m, 2H), 2.25 (t, J = 6.9, 2H), 1.68 (s, 3H); ^{31}P NMR (D_2O) δ 4.37. Two alkene signals obscured by the HOD peak. See Supplementary Figs. 73 and 74.

NMR Spectra of standards

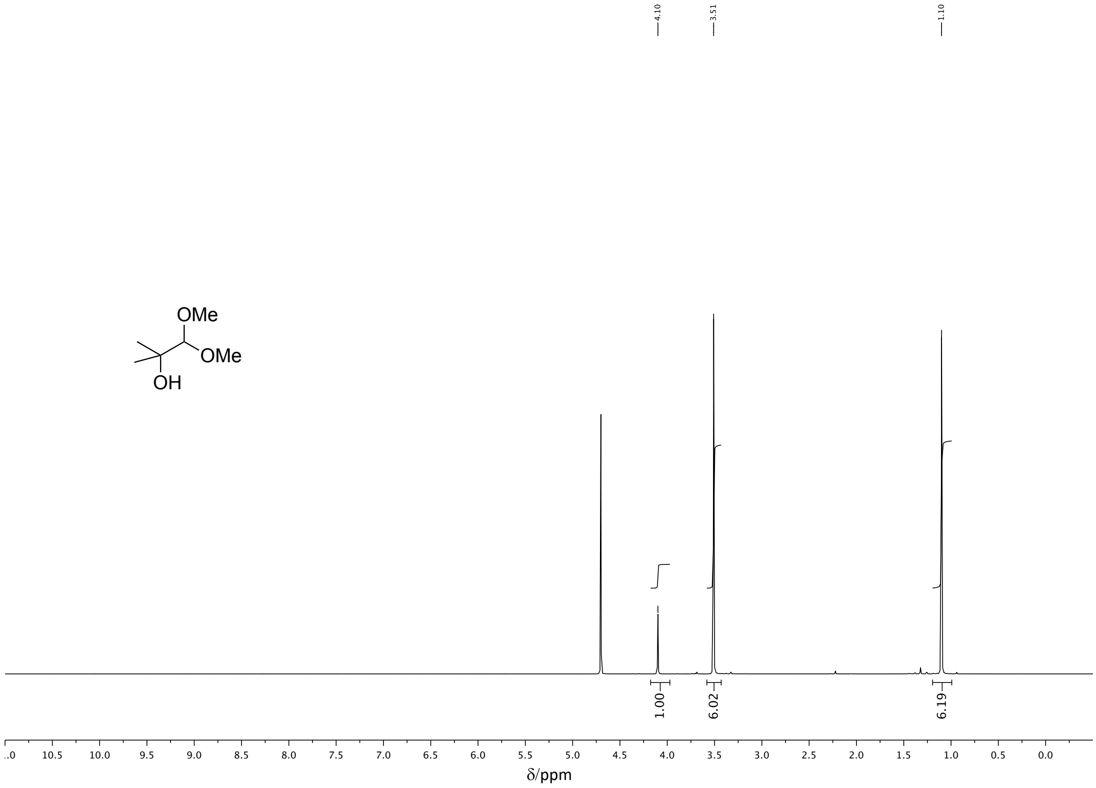

Supplementary Fig. 63

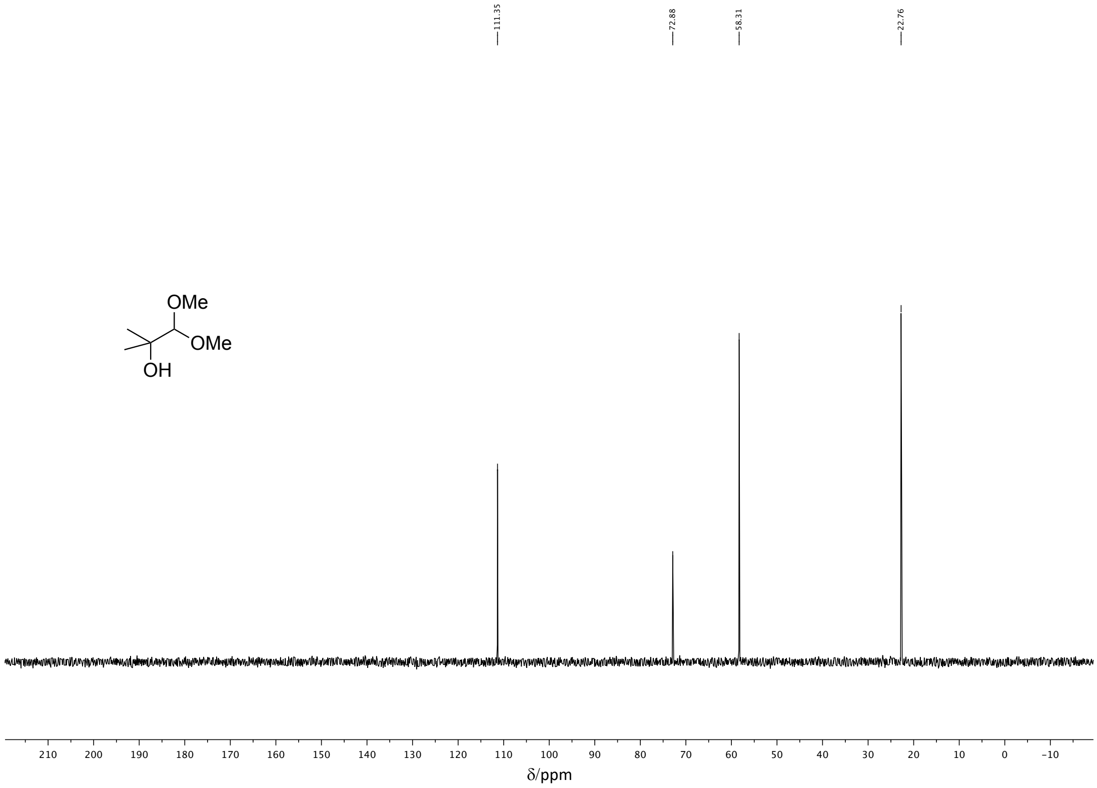

Supplementary Fig. 64

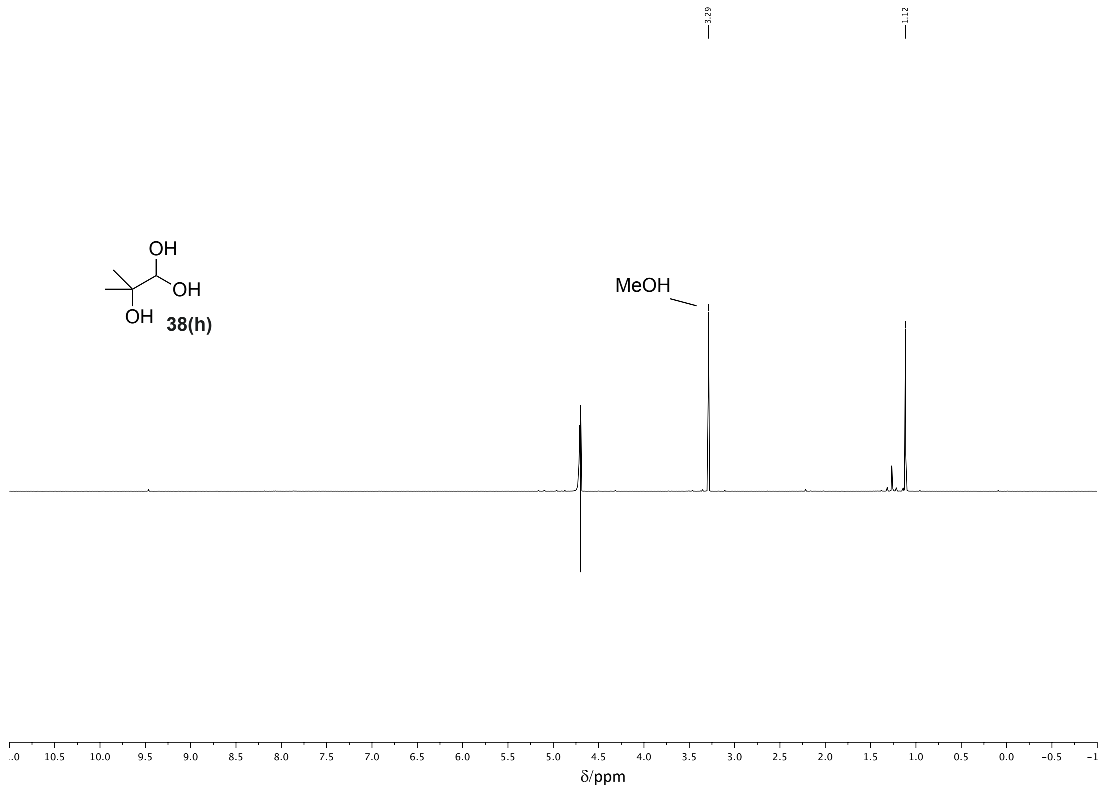

Supplementary Fig. 65 Signal at 3.29 ppm due to MeOH liberated in the acetal hydrolysis.

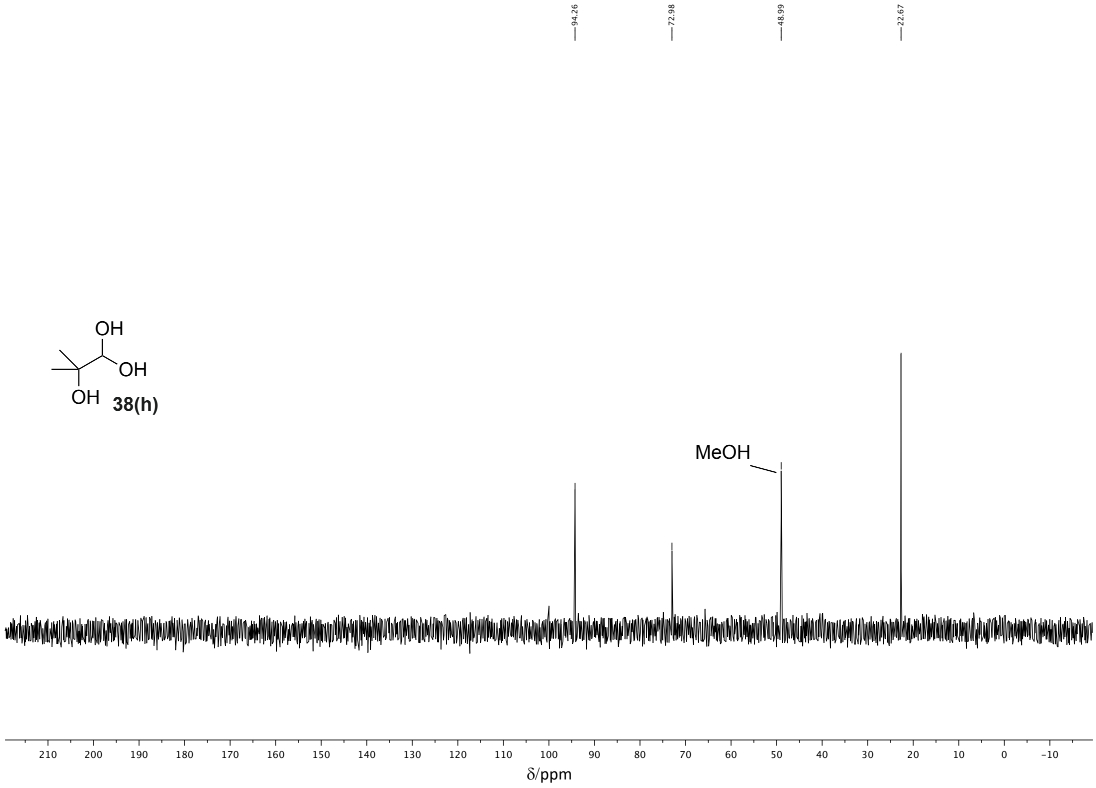

Supplementary Fig. 66 Signal at 49.0 ppm due to MeOH liberated in the acetal hydrolysis.

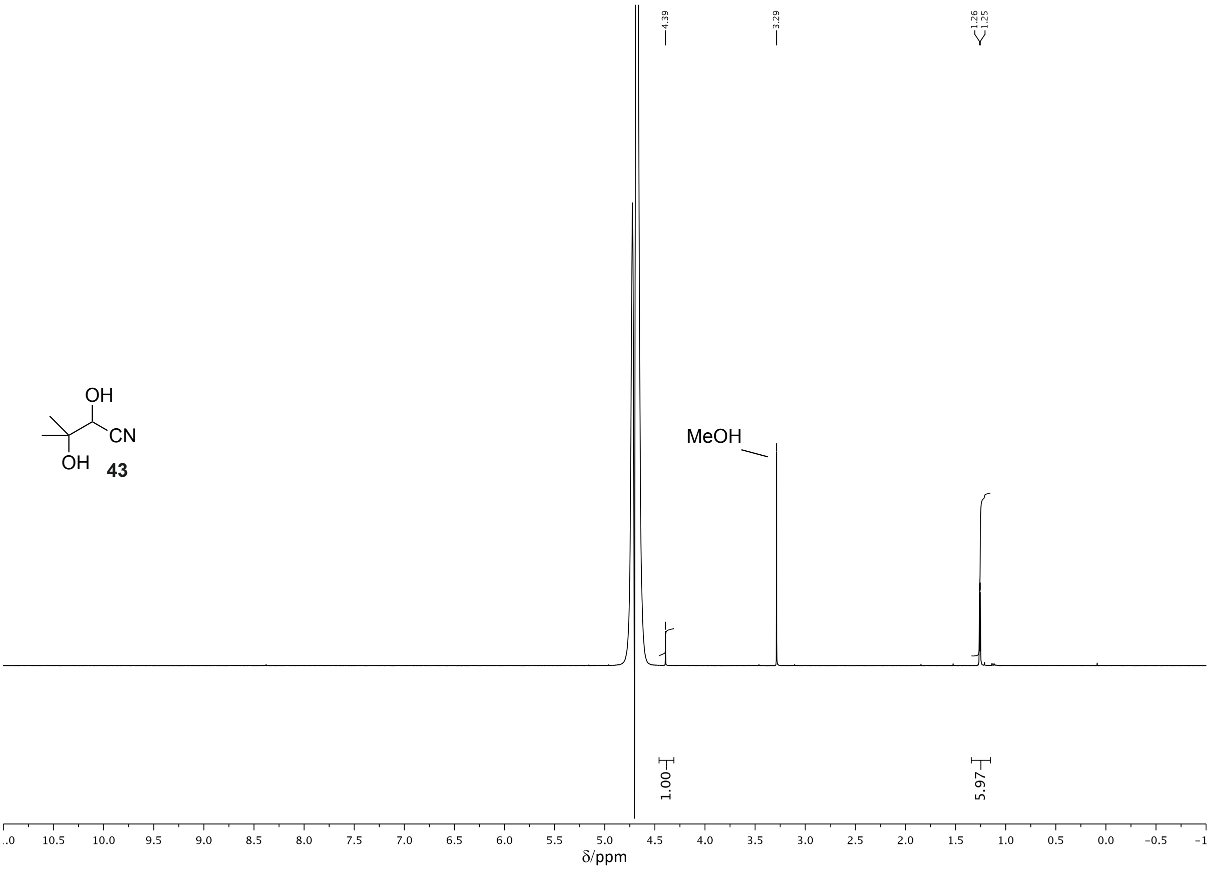

Supplementary Fig. 67 Signal at 3.29 ppm due to MeOH liberated in the acetal hydrolysis, see procedure.

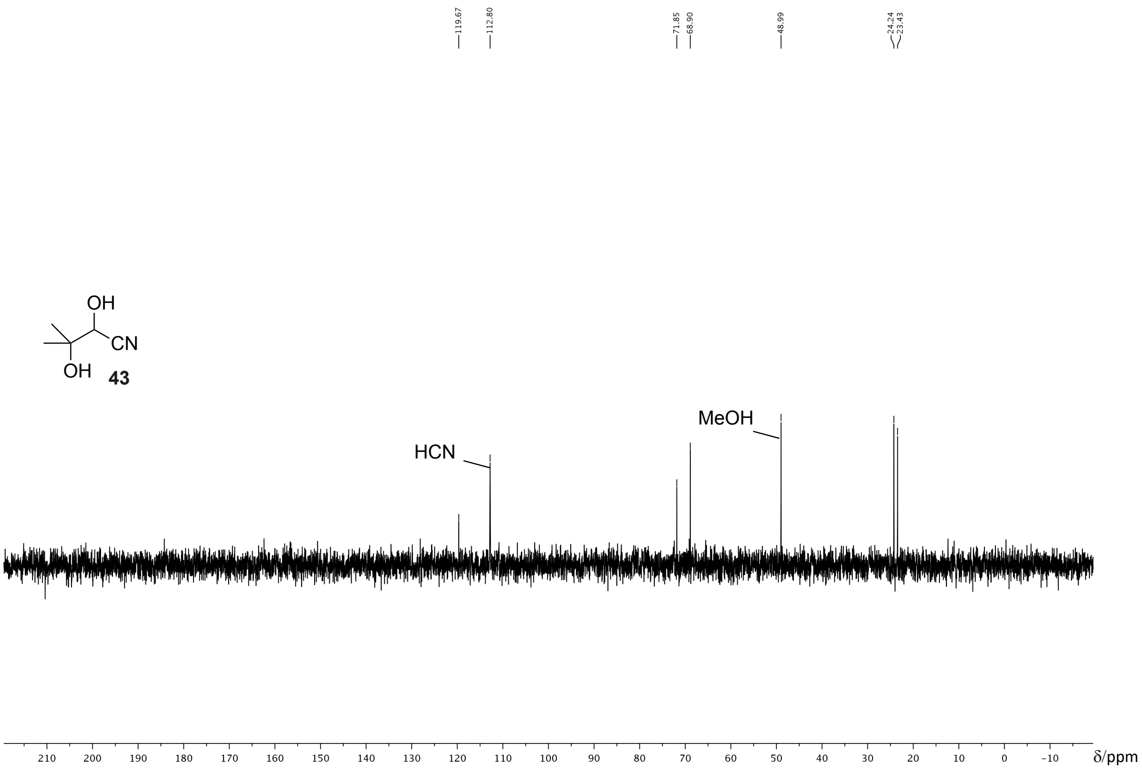

Supplementary Fig. 68 Signal at 49.0 ppm due to MeOH from acetal hydrolysis (see procedure), signal at 112.8 due to excess HCN.

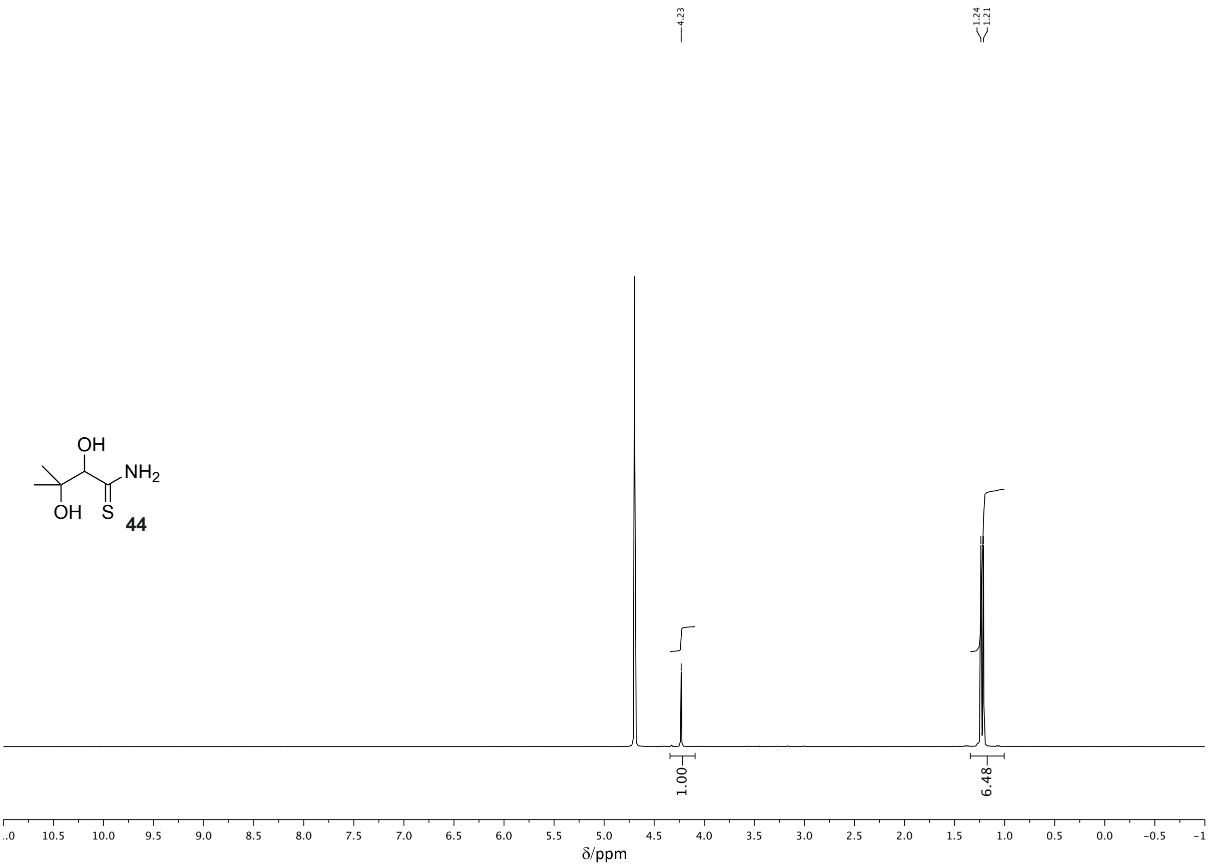

Supplementary Fig. 69

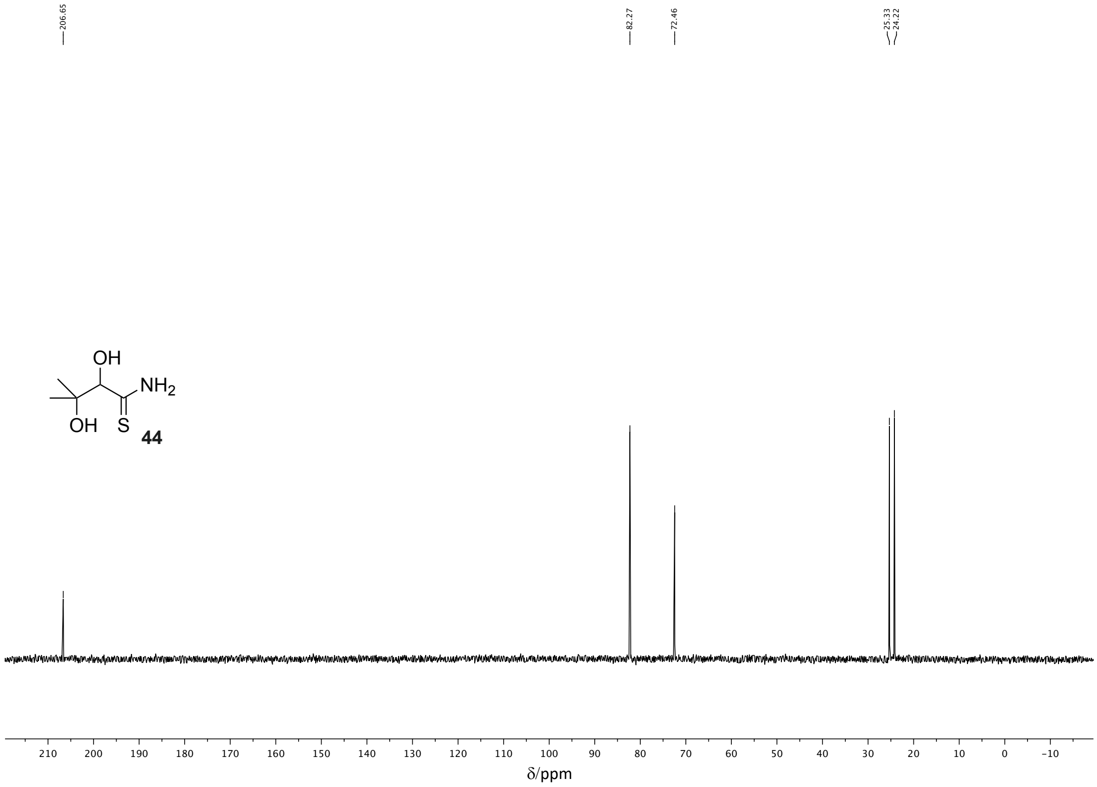

Supplementary Fig. 70

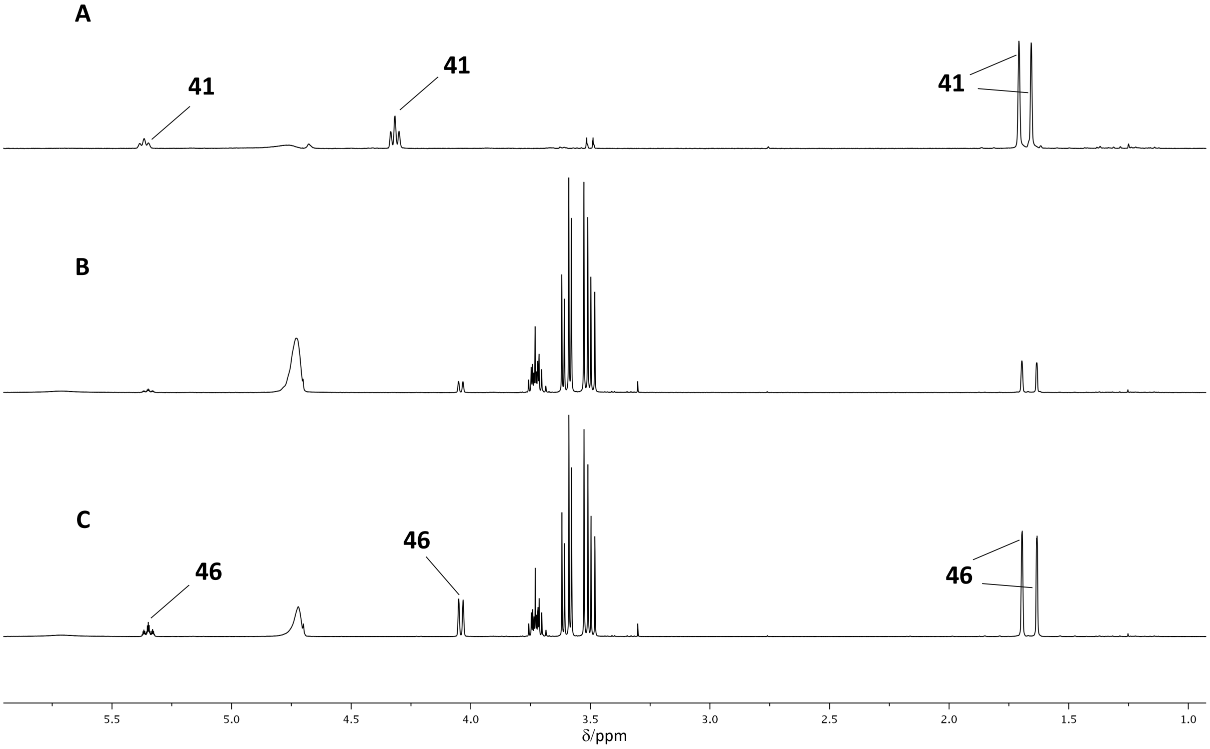

Supplementary Fig. 71. A – ^{1}H NMR Spectrum of the product from the procedure used to prepare dimethylallyl phosphate **41** (see Synthetic procedures to make standards, p. 76); B – As spectrum A, after the addition of $MgCl_2$ (1.5 mg) and NaCl (6 mg), pH adjusted to 8.2 and alkaline phosphatase solution added (1000 units, 3 µL). Incubated for 18 h at 37 °C. Alkaline phosphatase cleaves any monosubstituted phosphate group *i.e.* $ROPO_3^{2-}$, yielding orthophosphate and the hydroxy R-group, ROH. The complex set of signals 3.47 – 3.76 ppm are due to glycerol present in the commercial phosphatase solution; C – As spectrum B, after spiking with commercial dimethylallyl alcohol **46**. As dimethylallyl pyrophosphate is absent from the ^{31}P NMR spectrum (see Supplementary Fig. 72), we assigned the compound in spectrum A to dimethylallyl phosphate **41**. The doublet which is seen at 3.4 ppm in spectrum A is due to methyl phosphate and resulted from the phosphorylation of MeOH which was present in commercial formamide that was used for the phosphorylation reaction. After phosphatase treatment MeOH is regenerated, see spectrum B, singlet at 3.30 ppm.

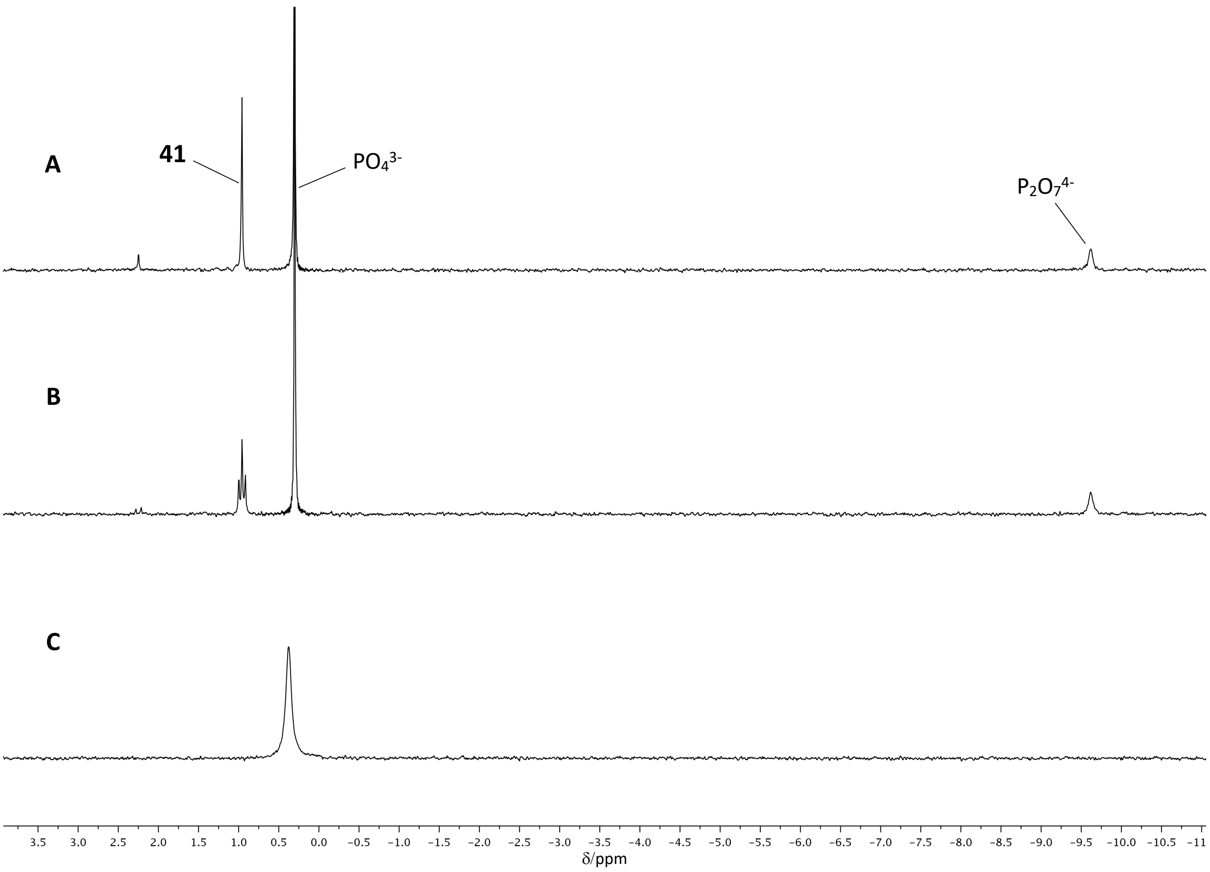

Supplementary Fig. 72 A – ^{31}P NMR Spectrum of the same sample as in Supplementary Fig. 71, spectrum A; B – Same sample as spectrum A, but run with ^{1}H-^{31}P coupling; C – ^{31}P NMR Spectrum of the same sample as in Supplementary Fig. 71, spectrum B. In spectrum A, the singlets at 0.30 and -9.60 ppm are due to orthophosphate and pyrophosphate, respectively (also confirmed by sample spiking). The singlet at 2.25 ppm is due to methyl phosphate (see Supplementary Fig. 71 legend) and in spectrum B is split into a quartet (see Supplementary Fig. 74 for more clarity). We assign the singlet at 0.96 ppm to dimethylallyl phosphate **41**.

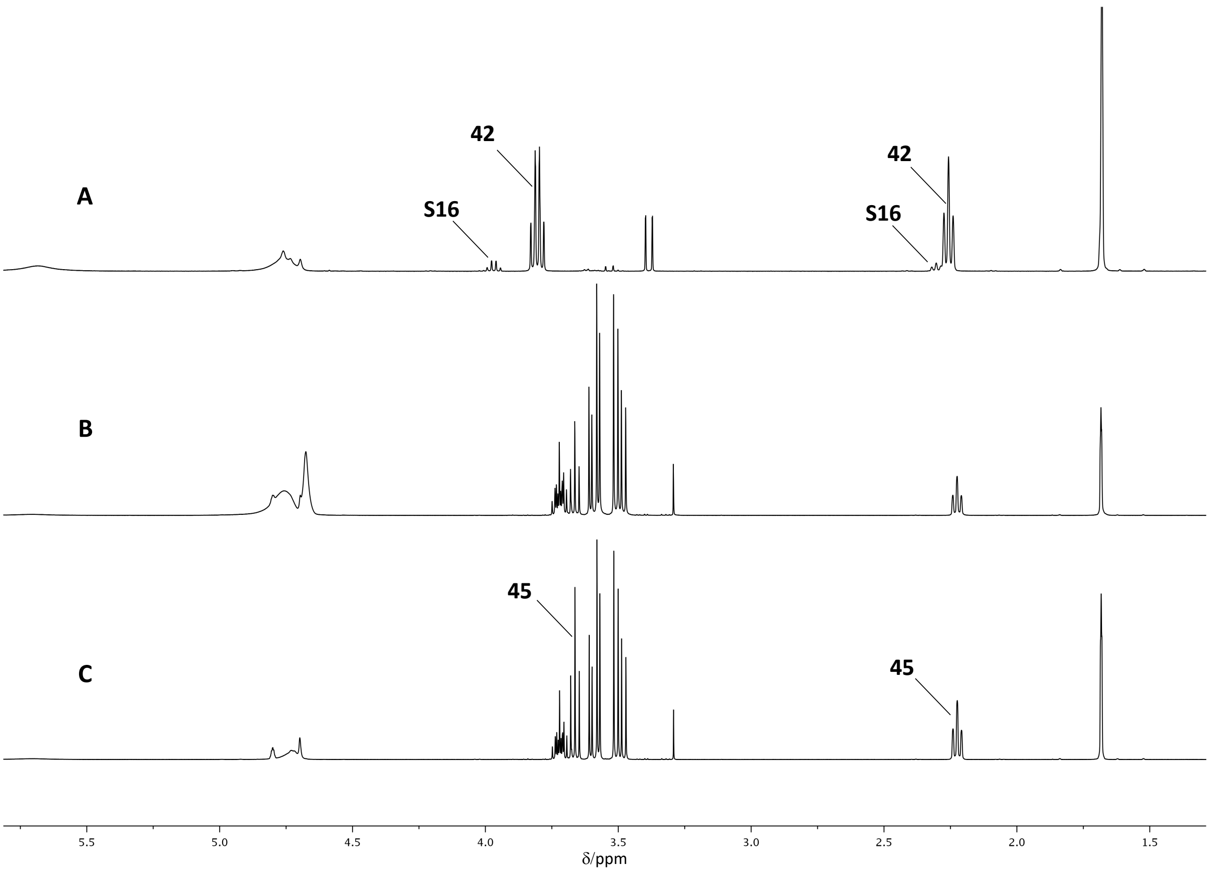

Supplementary Fig. 73 A – ^{1}H NMR Spectrum of the product of the procedure used to prepare isopentenyl phosphate **42** (see Synthetic procedures to make standards, p. 76); B – As spectrum A, after the addition of $MgCl_2$ (1.5 mg) and NaCl (6 mg), pH adjusted to 8.2 and alkaline phosphatase solution added (1000 units, 3 µL). Incubated for 18 h at 37 °C. Alkaline phosphatase cleaves any monosubstituted phosphate group *i.e.* $ROPO_3^{2-}$, yielding orthophosphate and the hydroxy R-group, ROH. The complex set of signals 3.47 – 3.76 ppm are due to glycerol present in the commercial phosphatase solution; C – As spectrum B, after spiking with commercial isopentenyl alcohol **45**. In spectrum A, we assign the major product as isopentenyl phosphate **42** [alkene signals obscured by HOD, 3.80 (m, 2H), 2.25 (t, *J* = 6.9, 2H), 1.68 (s, 3H)] and the minor compound as isopentenyl pyrophosphate **S16** [alkene signals obscured by HOD, 3.97 (q, *J* = 6.7, 2H), 2.30 (t, *J* = 6.5, 2H), 1.68 (s, 3H. The formation of **S16** was indicated by the conversion of the signals into isopentenyl alcohol **45** after phosphatase treatment and by ^{31}P NMR data (see Supplementary Fig. 74). The doublet which is seen at 3.38 ppm in spectrum A is due to methyl phosphate and resulted from the phosphorylation of MeOH which was present in commercial formamide that was used for the phosphorylation reaction. After phosphatase treatment MeOH is regenerated, see spectrum B, singlet at 3.30 ppm.

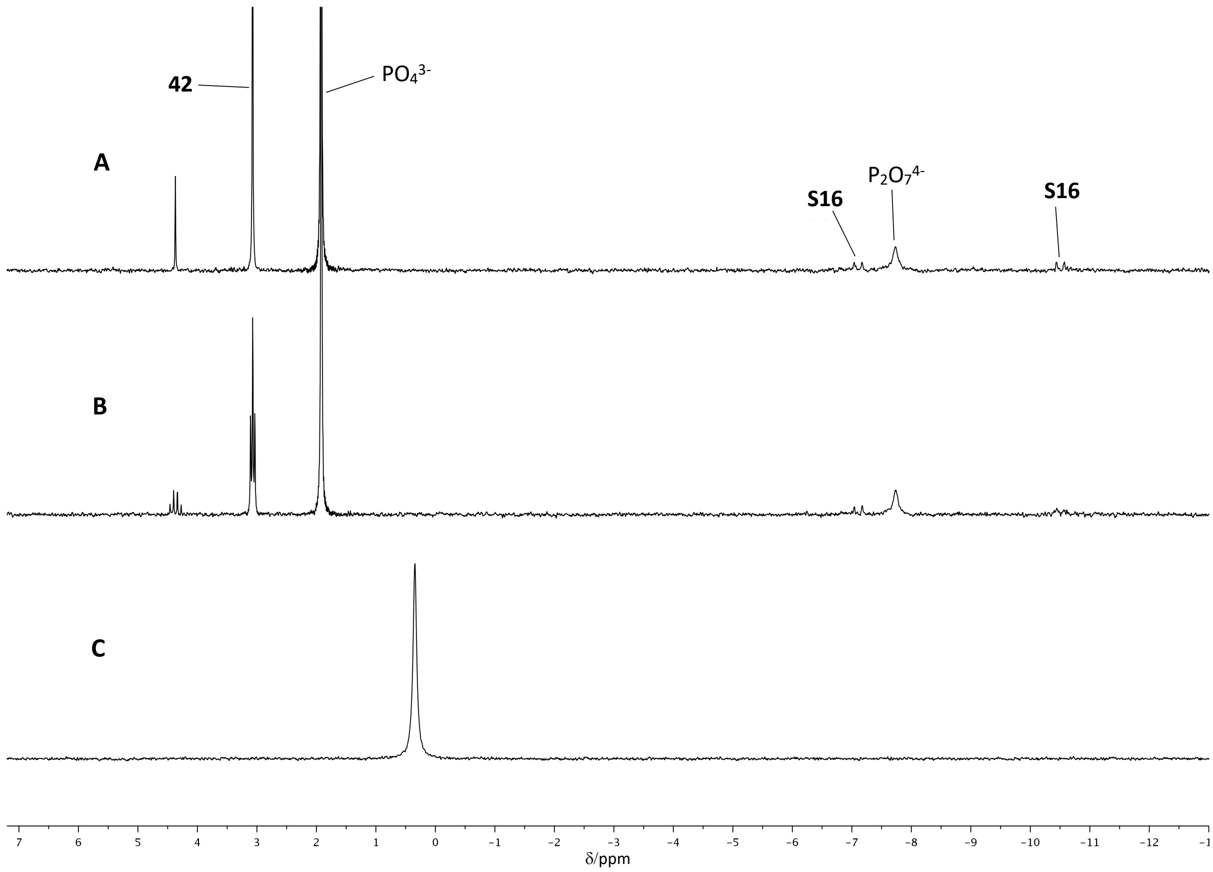

Supplementary Fig. 74 A – ^{31}P NMR Spectrum of the same sample as in Supplementary Fig. 73, spectrum A; B – Same sample as spectrum A, but run with ^{1}H-^{31}P coupling; C – ^{31}P NMR Spectrum of the same sample as in Supplementary Fig. 73, spectrum B. In spectrum A, the singlets at 1.9 and -7.70 ppm are due to orthophosphate and pyrophosphate, respectively (also confirmed by sample spiking, not shown). The singlet at 4.37 ppm is due to methyl phosphate (see Supplementary Fig. 73 legend) and in spectrum B is split into a quartet. We assign the singlet at 3.10 ppm to isopentenyl phosphate **42**. The two doublets that are coupling to each other [-7.10 ppm (*J* = 21.1) and -10.51 ppm (*J* = 21.1)] we ascribe to isopentenyl pyrophosphate **S16**, as the up-field signal is split in the ^{1}H-^{31}P coupling NMR experiment (spectrum B) but the down-field signal is not and the compound is digested by phosphatase (spectrum C) returning isopentenyl alcohol **45** (Supplementary Fig. 73).

Control experiments in 'oxygenated' solvents

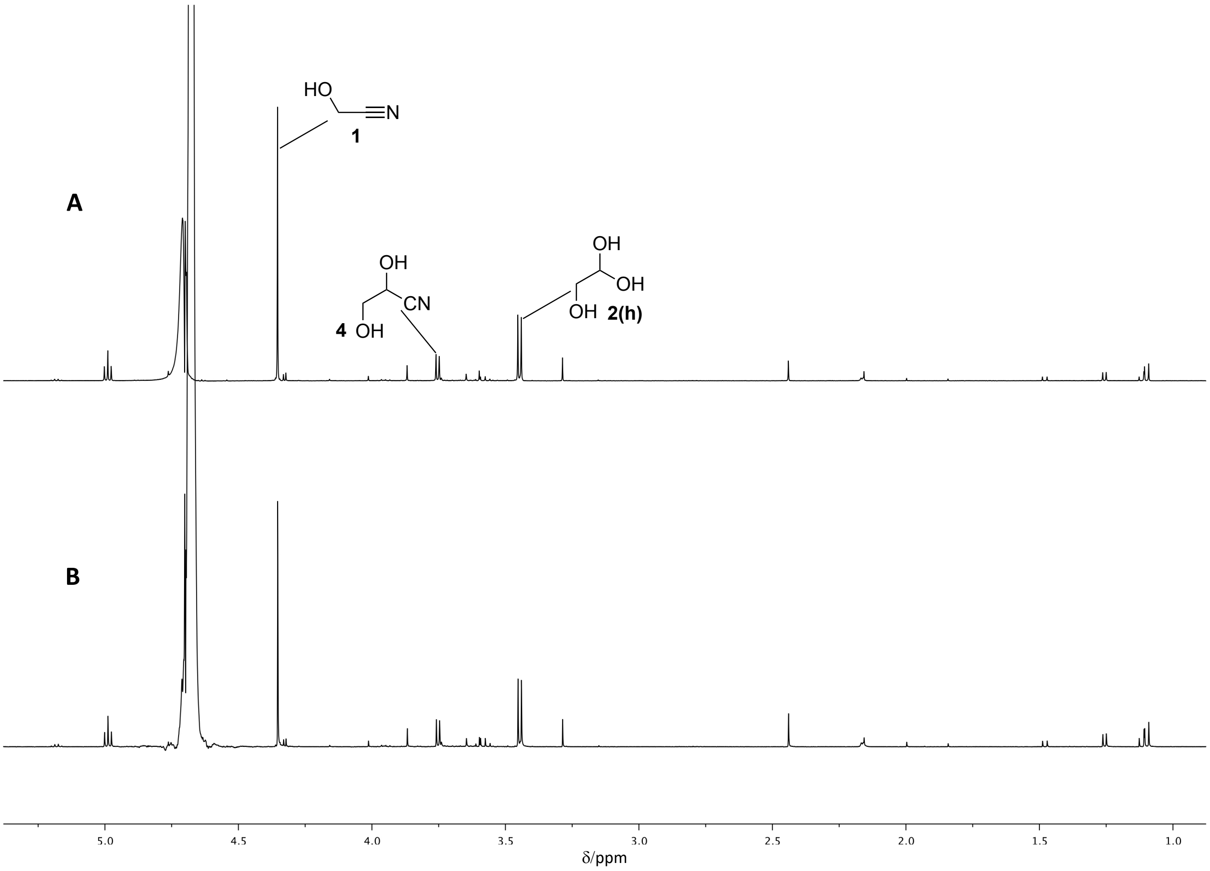

Supplementary Fig. 75 Photochemical reduction of glycolonitrile **1** using Na_3PSO_3 in H_2O/D_2O and degassed H_2O/D_2O. A – ^1H NMR Spectrum of the reaction according to Procedure 1 (1 equiv. Na_3PSO_3) after 1 h irradiation in 10% D_2O in H_2O which was degassed; B – ^1H NMR Spectrum of the reaction according to Procedure 1 (1 equiv. Na_3PSO_3) after 1 h irradiation in 10% D_2O in H_2O which was not degassed.

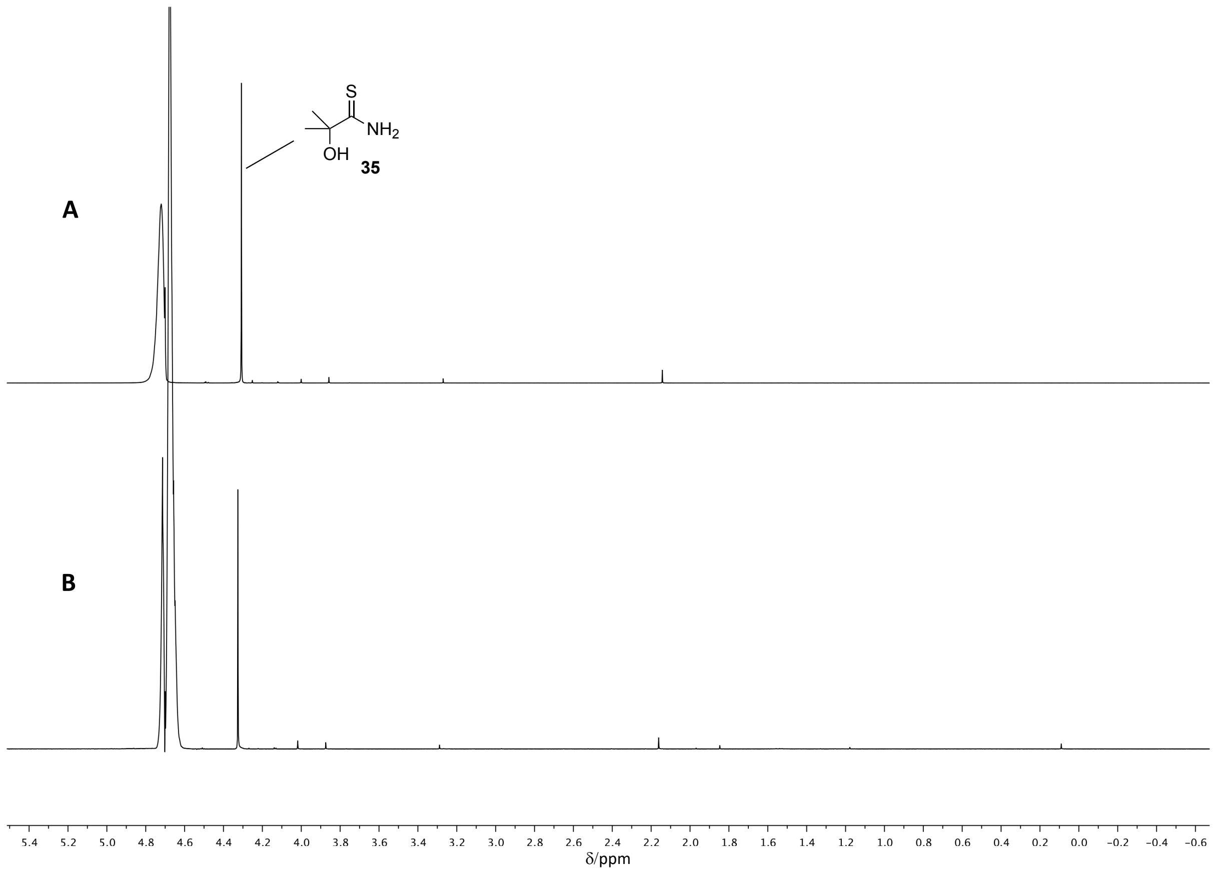

Supplementary Fig. 76 Thiolysis of glycolonitrile **1** using Na_3PSO_3 in H_2O/D_2O and degassed H_2O/D_2O. Glycolonitrile (15 mM) and $Na_3PSO_3.xH_2O$ (60 mM) incubated at 60 °C and pH 6.5 in 10% D_2O in H_2O for 24 h. A – ^1H NMR Spectrum of the reaction carried out in degassed solvent; B – ^1H NMR Spectrum of the reaction carried out in solvent which was not degassed.

Supplementary Discussion 1

The formation of multiple useful products from a common starting material, intermediate or reagent would have assisted the localisation of life's potential building blocks. An illustrative example of how this can occur from our prebiotic scheme (Supplementary Fig. 1)[1] follows: the photochemical reduction of glycolonitrile **1** gives glycolaldehyde **2**, but if further reducing equivalents are present in the system and little HCN remains, **2** can undergo α-deoxygenation to give acetaldehyde **3** (Supplementary Fig. 77).[1,16] Under Strecker conditions (NH_4CN and ensuing hydrolysis), this group of compounds leads to the amino acids glycine, serine and alanine (Supplementary Fig. 77, top). However, if NH_3 is absent from the same mixture, HCN will add to **2** and **3** forming glyceronitrile **4** and lactonitrile **5**, and these cyanohydrins can be further reduced to glyceraldehyde **7** and lactaldehyde **S17**.[1,16] Strecker reaction of **S17** leads to threonine, but under the same conditions the terminal hydroxy group of **S18** immediately cyclises onto the nitrile giving the dead end iminolactone **S19**, barring synthesis of the non-proteogenic amino acid 2-amino-3,4-dihydroxybutanoate. If no reducing reagent remains, **4** and **5** slowly hydrolyse to glycerate **S20** and lactate **S21**, respectively, and phosphorylated variants of **S20** are central to glycolysis whereas **S21** can enter the citric acid cycle after oxidation *via* pyruvate. Furthermore, glycolaldehyde **2** and glyceraldehyde **7** are intermediates in prebiotic nucleotide synthesis.[5,26,27]

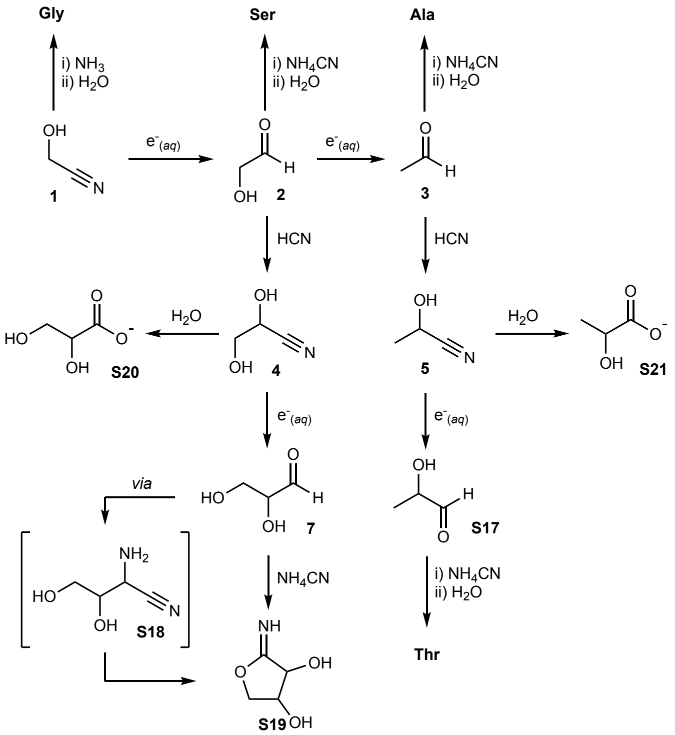

Supplementary Fig. 77 An example of the systems chemistry inherent to the reductive cyanosulfidic network previously reported.[1,16] Depending upon conditions the intermediates can undergo various transformations, many of which result in biomolecules or precursors of biomolecules. The photochemical one-electron reduction steps may proceed by a solvated electron ($e^-_{(aq)}$) or a hydrogen atom ($H^\bullet$), or both. As $H^\bullet$ is produced from the initial photoproduct, $e^-_{(aq)}$, and several reducing systems have been identified, we generalise this reduction step as '$e^-_{(aq)}$' for simplicity in Supplementary Fig. 77. After reduction of a nitrile, the corresponding aldehydes are formed upon hydrolysis of the intermediate imine. For convenience, unidirectional reaction arrows are used and aldehydes are depicted as carbonyl compounds, although they would also exist as hydrates in water.

Supplementary Discussion 2

The discovery that thiophosphate is produced during the oxidation of reduced phosphorus species by H_2S/HS^- and UV light,[14] or by aqueous alteration of schreibersite by HS^-/H_2S containing water (*vide infra*), is indicative that PSO_3^{3-} should have been accessible in some locations on Hadean Earth, most likely in the vicinity of meteorite impacts/debris. We previously pointed out that thiophosphate undergoes hydrolysis with a half-life of ~ 17 days at neutral pH and ambient temperature,[15] contrary to reported hydrolysis data,[49] which we have now confirmed using quantitative ^{31}P NMR (Supplementary Fig. 56). Dittmer *et al.* also reported that PSO_3^{3-} is more stable at alkaline pH, which we also found, observing little degradation after 6 weeks at pH 12 (Supplementary Fig. 57). When Fe_3P (a schreibersite surrogate)[51] was allowed to react with a solution of NaSH, PSO_3^{3-} could be observed by ^{31}P NMR spectroscopy within 3 days and accumulated further over the remainder of the experiment (Supplementary Figs. 58 and 59). Furthermore, when a solution of NaSH was adjusted to pH ~ 7 and allowed to react with Fe_3P, the pH of the solution rose during the corrosion process, and after 4 weeks had reached pH ~ 11.4, PSO_3^{3-} again being present by ^{31}P NMR spectroscopy and accounting for ~ 10% of the soluble phosphorus species (Supplementary Fig. 60).

We note that oldhamite (and niningerite) is prevalent in enstatite chondrites[55] and is frequently associated with schreibersite and perryite,[56,57] often in similar abundance.[58] Therefore, the rapid weathering of oldhamite,[59] forming $Ca(OH)_2$ and H_2S, opens the possibility for the leaching of thiophosphate (and/or thiophosphite) directly from meteoritic debris at alkaline pH. Additionally, the photochemical oxidation of hypophosphite by HS^-/H_2S at neutral pH has been estimated to be > 50% complete within 1 – 10 days on primitive Earth,[60] and the oxidation has been shown to be unperturbed at alkaline pH, again forming PSO_3^{3-} in the process.[14] This data, coupled with the fact that PSO_3^{3-} is a stable solid, suggests a window of opportunity for the accumulation of PSO_3^{3-}, in some locations if not globally, on primitive Earth was viable. Importantly, it must be noted that PSO_3^{3-} can be made (from a mixture of $H_2PO_2^-$, HPO_3^{2-}, PO_4^{3-} and HS^-) and used *in situ*, and consequently the accumulation of PSO_3^{3-} *per se* for photochemical reductions is not required, and its rate of hydrolysis becomes a moot point (Supplementary Figs. 61 and 62, *cf.* Supplementary Fig. 8). Although crude, an estimate for the rate of reduction on early Earth can be made from the previous

study by Rimmer *et al.*[61] where the Rayonet photochemical reactor was estimated to be five orders of magnitude more intense than the young Sun, hence the photochemical reductions performed here would have taken place on the timescale of months to years rather than tens of minutes. However, we take this as an absolute lower limit as only wavelengths 252-256 nm are scaled to those reaching primitive Earth from the young Sun, and in reality, broadband emission from the young Sun would be expected to irradiate Earth's surface for all wavelengths > ~ 200 nm.[61,62]

References

44. D. Herschlag, Ribonuclease revisited: Catalysis via the classical general acid-base mechanism or a triester-like mechanism? *J. Am. Chem. Soc.* **116**, 11631-11635 (1994).
45. D. I. Loewus & F. Eckstein, Symmetrical monothiophosphate. *J. Am. Chem. Soc.* **105**, 3287-3292 (1983).
46. R. M. Phan & C. D. Poulter, Synthesis of (*S*)-isoprenoid thiodiphosphates as substrates and inhibitors. *J. Org. Chem.* **66**, 6705-6710 (2001).
47. M. Shirom & G. Stein, Excited state chemistry of the ferrocyanide ion in aqueous solution. I. Formation of the hydrated electron. *J. Chem. Phys.* **55**, 3372-3378 (1971).
48. A. C. Fahrenbach *et al.* Common and potentially prebiotic origin for precursors of nucleotide synthesis and activation. *J. Am. Chem. Soc.* **139**, 8780-8783 (2017).
49. D. C. Dittmer & O. B. Ramsay, Reactivity of thiophosphates. I. Hydrolysis of phosphorothioic acid. *J. Org. Chem.* **28**, 1268-1272 (1963).
50. A. E. Nagul *et al.* The molybdenum blue reaction for the determination of orthophosphate revisited: Opening the black box. *Anal. Chim. Acta* **890**, 60-82 (2015).
51. C. Pirim *et al.* Investigation of schreibersite and intrinsic oxidation products from Sikhote-Alin, Seymchan, and Odessa meteorites and Fe_3P and Fe_2NiP synthetic surrogates. *Geochim. Cosmochim. Acta* **140**, 259-274 (2014).
52. N. Yoza *et al.* Oxidative conversion of diphosphonate to diphosphate and isohypophosphate by hydrogen peroxide. *Chem. Lett.* **19**, 1487-1490 (1990).
53. H. Maki, Y. Ueda & H. Nariai, Protonation equilibria and stepwise hydrolysis behavior of a series of thiomonophosphate anions. *J. Phys. Chem. B* **115**, 3571-3577 (2011).
54. W-D. Fessner, C. Goße, G. Jaeschke & O. Eyrisch, Enzymes in organic synthesis, 15[≠] short enzymatic synthesis of L-fucose analogs. *Eur. J. Org. Chem.* 125-132 (2000).
55. L. Piani, Y. Marrocchi, G. Libourel & L. Tissander, Magmatic sulfides in the porphyritic chrondules of EH enstatite chondrites. *Geochim. Cosomochim. Acta* **195**, 84-99 (2016).
56. S. W. Lehner, P. B. Buseck & W. F. McDonough, Origin of kamacite, schreibersite, and perryite in metal-sulfide nodules of the enstatite chondrite Sahara 97072 (EH3). *Meteorit. Planet. Sci.* **45**, 289-303 (2010).
57. W. Hsu, Geochemical and petrographical studies of oldhamite, diopside and roedderite in enstatite meteorites. *Meteorit. Planet. Sci.* **33**, 291-301 (1998).

58. R. A. Fogel, On the significance of diopside and oldhamite in enstatite chondrites and aubrites. *Meteorit. Planet. Sci.* **32**, 577-591 (1997).

59. P. Jenniskens *et al.* Radar-enabledrecovery of the Stutter's Mill meteorite, a carbonaceous chondrite regolith breccia. *Science* **338**, 1583-1587 (2012).

60. P. B. Rimmer *et al.* Timescales for prebiotic photochemistry under realistic surface ultraviolet conditions. *Astrobiol.* **21**, doi.org/10.1089/ast.2020.2335 (2021).

61. P. B. Rimmer *et al.* The origin of RNA precursors on exoplanets. *Sci. Adv.* **4**, Article no. eaar3302 (2018)

62. S. Ranjan & D. D. Sasselov, Influence of the UV environment on the synthesis of prebiotic molecules. *Astrobiology* **16**, 68–88 (2016).
